# Supplementary material for: Systematic review to investigate the safety of induction and augmentation of labour among pregnant women with iron-deficiency anaemia
Source: BMJ Open. 2018 Dec 19;8(12):e021793. doi: 10.1136/bmjopen-2018-021793 (PMC6303660; doi:10.1136/bmjopen-2018-021793)
Supplement: Supplementary file 1 [file bmjopen-2018-021793supp001.pdf]

S1 Text

Search strategies

Medline

# ▲	Searches
1	anemia/ or anemia, hypochromic/ or anemia, iron-deficiency/
2	(anaemia or anemia).ti.
3	((iron deficien* or hypochromic or microcytic) adj5 (anaemia or anemia)).ti,ab.
4	((pregnan* or gestation* or maternal) and (anaemia or anemia)).ti,ab.
5	(Pregnancy/ or Pregnant woman/) and (anaemia or anemia).mp.
6	1 or 2 or 3 or 4 or 5
7	exp Labor, Obstetric/
8	exp Parturition/
9	exp Delivery, Obstetric/
10	(labor or labour or birth? or childbirth? or delivery or obstetric*).ti.
11	((induction? or induced or inducing) adj5 (labor or labour or birth? or childbirth? or delivery)).ti,ab.
12	(augment* adj5 (labor or labour or birth? or childbirth? or delivery)).ti,ab.
13	(manage* adj5 (labor or labour or birth? or childbirth? or delivery)).ti,ab.
14	(initiat* adj5 (labor or labour or birth? or childbirth? or delivery)).ti,ab.
15	Oxytocin/
16	exp Prostaglandins E, Synthetic/
17	(uterotonic? or oxytocin or prostaglandin? or misoprostol).ti,ab.
18	7 or 8 or 9 or 10 or 11 or 12 or 13 or 14 or 15 or 16 or 17
19	Developing Countries/
20	(Africa or Caribbean or West Indies or South America or Latin America or Central America).hw,kf,ti,ab,cp.

21	(Afghanistan or Albania or Algeria or Angola or American Samoa or Armenia or Armenian or Azerbaijan or Bangladesh or Benin or Byelarus or Byelorussian or Belarus or Belorussian or Belorussia or Belize or Bhutan or Bolivia or Bosnia or Herzegovina or Hercegovina or Botswana or Brazil or Brasil or Bulgaria or Burkina Faso or Burkina Fasso or Upper Volta or Burundi or Urundi or Cambodia or Khmer Republic or Kampuchea or Cameroon or Cameroons or Cameron or Camerons or Cape Verde or Central African Republic or Chad or China or Colombia or Comoros or Comoro Islands or Comores or Mayotte or Congo or Zaire or Costa Rica or Cote d'Ivoire or Ivory Coast or Cuba or Djibouti or French Somaliland or Dominica or Dominican Republic or East Timor or East Timur or Timor Leste or Ecuador or Egypt or United Arab Republic or El Salvador or Eritrea or Ethiopia or Fiji or Gabon or Gabonese Republic or Gambia or Gaza or Georgia Republic or Georgian Republic or Ghana or Gold Coast or Grenada or Guatemala or Guinea or Guinea-Bisau or Guam or Guiana or Guyana or Haiti or Honduras or India or Maldives or Indonesia or Iran or Iraq or Jamaica or Jordan or Kazakhstan or Kazakh or Kenya or Kiribati or Korea or Kosovo or Kyrgyzstan or Kirghizia or Kyrgyz Republic or Kirghiz or Kirgizstan or Lao PDR or Laos or Lebanon or Lesotho or Basutoland or Liberia or Libya or Macedonia or Madagascar or Malagasy Republic or Malaysia or Malaya or Malay or Sabah or Sarawak or Malawi or Nyasaland or Mali or Marshall Islands or Mauritania or Mauritius or Agalega Islands or Mexico or Micronesia or Middle East or Moldova or Moldovia or Moldovian or Mongolia or Montenegro or Morocco or Ifni or Mozambique or Myanmar or Myanma or Burma or Namibia or Nepal or Netherlands Antilles or Nicaragua or Niger or Nigeria or Pakistan or Palau or Palestine or Panama or Papua New Guinea or Paraguay or Peru or Philippines or Philipines or Phillipines or Phillippines or Romania or Rumania or Roumania or Rwanda or Ruanda or Saint Lucia or St Lucia or Saint Vincent or St Vincent or Grenadines or Samoa or Samoan Islands or Navigator Island or Navigator Islands or Sao Tome or Senegal or Serbia or Sierra Leone or Sri Lanka or Ceylon or Solomon Islands or Somalia or Sudan or Suriname or Surinam or Swaziland or Syria or Principe or South Sudan or Tajikistan or Tadzhikistan or Tadjikistan or Tadzhik or Tanzania or Thailand or Timor-Leste or Togo or Togolese Republic or Tonga or Tunisia or Turkey or Turkmenistan or Turkmen or Tuvalu or Uganda or Ukraine or Uzbekistan or Uzbek or Vanuatu or New Hebrides or Vietnam or Viet Nam or West Bank or Yemen or Zambia or Zimbabwe or Rhodesia).hw,kf,ti,ab,cp.
22	((developing or less* developed or under developed or underdeveloped or middle income or low* income or underserved or under served or deprived or poor*) adj (countr* or nation? or state? or population? or world)).ti,ab.
23	((developing or less* developed or under developed or underdeveloped or middle income or low* income) adj (economy or economies)).ti,ab.
24	(low* adj (gdp or gnp or gross domestic or gross national)).ti,ab.
25	(low adj3 middle adj3 countr*).ti,ab.
26	(lmic or lmics or third world or lami countr*).ti,ab.
27	transitional countr*.ti,ab.
28	19 or 20 or 21 or 22 or 23 or 24 or 25 or 26 or 27
29	6 and 18 and 28

Embase

# ▲	Searches
1	*anemia/ or iron deficiency anemia/ or microcytic anemia/
2	(anaemia or anemia).ti.
3	((iron deficien* or hypochromic or microcytic) adj5 (anaemia or anemia)).ti,ab.
4	((pregnan* or gestation* or maternal) and (anaemia or anemia)).ti,ab.
5	(Pregnancy/ or Pregnant woman/) and (anaemia or anemia).mp.
6	1 or 2 or 3 or 4 or 5
7	exp labor/
8	childbirth/
9	exp delivery/
10	(labor or labour or birth? or childbirth? or delivery or obstetric*).ti.
11	((induction? or induced or inducing) adj5 (labor or labour or birth? or childbirth? or delivery)).ti,ab.
12	(augment* adj5 (labor or labour or birth? or childbirth? or delivery)).ti,ab.
13	(manage* adj5 (labor or labour or birth? or childbirth? or delivery)).ti,ab.
14	(initiat* adj5 (labor or labour or birth? or childbirth? or delivery)).ti,ab.
15	exp uterotonic agent/
16	(uterotonic? or oxytocin or prostaglandin? or misoprostol).ti,ab.
17	7 or 8 or 9 or 10 or 11 or 12 or 13 or 14 or 15 or 16
18	Developing Country.sh.
19	(Africa or Caribbean or West Indies or South America or Latin America or Central America).hw,ti,ab,cp.

20	(Afghanistan or Albania or Algeria or Angola or American Samoa or Armenia or Armenian or Azerbaijan or Bangladesh or Benin or Byelarus or Byelorussian or Belarus or Belorussian or Belorussia or Belize or Bhutan or Bolivia or Bosnia or Herzegovina or Hercegovina or Botswana or Brazil or Brasil or Bulgaria or Burkina Faso or Burkina Fasso or Upper Volta or Burundi or Urundi or Cambodia or Khmer Republic or Kampuchea or Cameroon or Cameroons or Cameron or Camerons or Cape Verde or Central African Republic or Chad or China or Colombia or Comoros or Comoro Islands or Comores or Mayotte or Congo or Zaire or Costa Rica or Cote d'Ivoire or Ivory Coast or Cuba or Djibouti or French Somaliland or Dominica or Dominican Republic or East Timor or East Timur or Timor Leste or Ecuador or Egypt or United Arab Republic or El Salvador or Eritrea or Ethiopia or Fiji or Gabon or Gabonese Republic or Gambia or Gaza or Georgia Republic or Georgian Republic or Ghana or Gold Coast or Grenada or Guatemala or Guinea or Guinea-Bisau or Guam or Guiana or Guyana or Haiti or Honduras or India or Maldives or Indonesia or Iran or Iraq or Jamaica or Jordan or Kazakhstan or Kazakh or Kenya or Kiribati or Korea or Kosovo or Kyrgyzstan or Kirghizia or Kyrgyz Republic or Kirghiz or Kirgizstan or Lao PDR or Laos or Lebanon or Lesotho or Basutoland or Liberia or Libya or Macedonia or Madagascar or Malagasy Republic or Malaysia or Malaya or Malay or Sabah or Sarawak or Malawi or Nyasaland or Mali or Marshall Islands or Mauritania or Mauritius or Agalega Islands or Mexico or Micronesia or Middle East or Moldova or Moldovia or Moldovian or Mongolia or Montenegro or Morocco or Ifni or Mozambique or Myanmar or Myanma or Burma or Namibia or Nepal or Netherlands Antilles or Nicaragua or Niger or Nigeria or Pakistan or Palau or Palestine or Panama or Papua New Guinea or Paraguay or Peru or Philippines or Philipines or Phillipines or Phillippines or Romania or Rumania or Roumania or Rwanda or Ruanda or Saint Lucia or St Lucia or Saint Vincent or St Vincent or Grenadines or Samoa or Samoan Islands or Navigator Island or Navigator Islands or Sao Tome or Senegal or Serbia or Sierra Leone or Sri Lanka or Ceylon or Solomon Islands or Somalia or Sudan or Suriname or Surinam or Swaziland or Syria or Principe or South Sudan or Tajikistan or Tadzhikistan or Tadjikistan or Tadzhik or Tanzania or Thailand or Timor-Leste or Togo or Togolese Republic or Tonga or Tunisia or Turkey or Turkmenistan or Turkmen or Tuvalu or Uganda or Ukraine or Uzbekistan or Uzbek or Vanuatu or New Hebrides or Vietnam or Viet Nam or West Bank or Yemen or Zambia or Zimbabwe or Rhodesia).hw,ti,ab,cp.
21	((developing or less* developed or under developed or underdeveloped or middle income or low* income or underserved or under served or deprived or poor*) adj (countr* or nation? or state? or population? or world)).ti,ab.
22	((developing or less* developed or under developed or underdeveloped or middle income or low* income) adj (economy or economies)).ti,ab.
23	(low* adj (gdp or gnp or gross domestic or gross national)).ti,ab.
24	(low adj3 middle adj3 countr*).ti,ab.
25	(lmic or lmics or third world or lami countr*).ti,ab.
26	transitional countr*.ti,ab.
27	18 or 19 or 20 or 21 or 22 or 23 or 24 or 25 or 26
28	6 and 17 and 27

Global Health

# ▲	Searches
1	anaemia/ or hypochromic anaemia/ or iron deficiency anaemia/ or microcytic anaemia/
2	(anaemia or anemia).ti.
3	((iron deficien* or hypochrmomic or microcytic) adj5 (anaemia or anemia)).ti,ab.
4	((pregnan* or gestation* or maternal) and (anaemia or anemia)).ti,ab.
5	1 or 2 or 3 or 4
6	birth/ or childbirth/ or parturition/
7	(labor or labour or birth? or childbirth? or delivery or obstetric*).ti.
8	((induction? or induced or inducing) adj5 (labor or labour or birth? or childbirth? or delivery)).ti,ab.
9	(augment* adj5 (labor or labour or birth? or childbirth? or delivery)).ti,ab.
10	(manage* adj5 (labor or labour or birth? or childbirth? or delivery)).ti,ab.
11	oxytocin/
12	exp synthetic prostaglandins/
13	(uterotonic? or oxytocin or prostaglandin? or misoprostol).ti,ab.
14	6 or 7 or 8 or 9 or 10 or 11 or 12 or 13
15	exp developing countries/ or exp africa/
16	(Africa or Caribbean or West Indies or South America or Latin America or Central America).hw,ti,ab,cp.
17	(Afghanistan or Albania or Algeria or Angola or American Samoa or Armenia or Armenian or Azerbaijan or Bangladesh or Benin or Byelarus or Byelorussian or Belarus or Belorussian or Belorussia or Belize or Bhutan or Bolivia or Bosnia or Herzegovina or Hercegovina or Botswana or Brazil or Brasil or Bulgaria or Burkina Faso or Burkina Fasso or Upper Volta or Burundi or Urundi or Cambodia or Khmer Republic or Kampuchea or Cameroon or Cameroons or Cameron or Camerons or Cape Verde or Central African Republic or Chad or China or Colombia or Comoros or Comoro Islands or Comores or Mayotte or Congo or Zaire or Costa Rica or Cote d'Ivoire or Ivory Coast or Cuba or Djibouti or French Somaliland or Dominica or Dominican Republic or East Timor or East Timur or Timor Leste or Ecuador or Egypt or United Arab Republic or El Salvador or Eritrea or Ethiopia or Fiji or Gabon or Gabonese Republic or Gambia or Gaza or Georgia Republic or Georgian Republic or Ghana or Gold Coast or Grenada or Guatemala or Guinea or Guinea-Bisau or Guam or Guiana or Guyana or Haiti or Honduras or India or Maldives or Indonesia or Iran or Iraq or Jamaica or Jordan or Kazakhstan or Kazakh or Kenya or Kiribati or Korea or Kosovo or Kyrgyzstan or Kirghizia or Kyrgyz Republic or Kirghiz or Kirgizstan or Lao PDR or Laos or Lebanon or Lesotho or Basutoland or Liberia or Libya or Macedonia or Madagascar or Malagasy Republic or Malaysia or Malaya or Malay or Sabah or Sarawak or Malawi or Nyasaland or Mali or Marshall Islands or Mauritania or Mauritius or Agalega Islands or Mexico or Micronesia or Middle East or Moldova or Moldovia or Moldovian or Mongolia or Montenegro or Morocco or Ifni or Mozambique or Myanmar or Myanma or Burma or Namibia or Nepal or Netherlands Antilles or Nicaragua or Niger or Nigeria or Pakistan or Palau or Palestine or Panama or Papua New Guinea or Paraguay or Peru or Philippines or Philipines or Phillipines or Phillippines or Romania or Rumania or Roumania or Rwanda or Ruanda or Saint Lucia or St Lucia or Saint Vincent or St Vincent or Grenadines or Samoa or Samoan Islands or Navigator Island or Navigator Islands or Sao Tome or Senegal or Serbia or Sierra Leone or Sri Lanka or Ceylon or Solomon Islands or Somalia or Sudan or Suriname or Surinam or Swaziland or Syria or Principe or South Sudan or Tajikistan or Tadzhikistan or Tadjikistan or Tadzhik or Tanzania or Thailand or Timor-Leste or Togo or

	Togolese Republic or Tonga or Tunisia or Turkey or Turkmenistan or Turkmen or Tuvalu or Uganda or Ukraine or Uzbekistan or Uzbek or Vanuatu or New Hebrides or Vietnam or Viet Nam or West Bank or Yemen or Zambia or Zimbabwe or Rhodesia).hw,ti,ab,cp.
18	((developing or less* developed or under developed or underdeveloped or middle income or low* income or underserved or underserved or deprived or poor*) adj (countr* or nation? or state? or population? or world)).ti,ab.
19	((developing or less* developed or under developed or underdeveloped or middle income or low* income) adj (economy or economies)).ti,ab.
20	(low* adj (gdp or gnp or gross domestic or gross national)).ti,ab.
21	(low adj3 middle adj3 countr*).ti,ab.
22	(lmic or lmics or third world or lami countr*).ti,ab.
23	transitional countr*.ti,ab.
24	15 or 16 or 17 or 18 or 19 or 20 or 21 or 22 or 23
25	5 and 14 and 24

CINAHL

#	Query
1	(MH "Anemia") OR (MH "Anemia, Hypochromic") OR (MH "Anemia, Iron Deficiency")
2	TI (anaemia or anemia) OR AB (((iron deficien* or hypochromic or microcytic) N5 (anaemia or anemia))) OR AB (((pregnan* or gestation* or maternal) and (anaemia or anemia)))
3	((MH "Pregnancy") OR (MH "Expectant Mothers")) AND (anaemia or anemia)
4	S1 OR S2 OR S3
5	(MH "Childbirth+") OR (MH "Labor+")
6	(MH "Delivery, Obstetric+")
7	TI (labor or labour or birth? or childbirth? or delivery or obstetric*) OR AB (((induction? or induced or inducing) N5 (labor or labour or birth? or childbirth? or delivery))) OR AB ((augment* N5 (labor or labour or birth? or childbirth? or delivery))) OR AB ((manage* N5 (labor or labour or birth? or childbirth? or delivery))) OR AB ((initiat* N5 (labor or labour or birth? or childbirth? or delivery)))
8	(MH "Oxytocics+")
9	(MH "Prostaglandins, Synthetic+")
10	TI (uterotonic? or oxytocin or prostaglandin? or misoprostol) OR AB (uterotonic? or oxytocin or prostaglandin? or misoprostol)
11	S5 OR S6 OR S7 OR S8 OR S9 OR S10
12	(MH "Developing Countries")
13	TI (Afghanistan or Albania or Algeria or Angola or American Samoa or Armenia or Armenian or Azerbaijan or Bangladesh or Benin or Byelarus or Byelorussian or Belarus or Belorussian or Belorussia or Belize or Bhutan or Bolivia or Bosnia or Herzegovina or Hercegovina or Botswana or Brazil or Brasil or Bulgaria or Burkina Faso or Burkina Fasso or Upper Volta or Burundi or Urundi or Cambodia or Khmer Republic or Kampuchea or Cameroon or Cameroons or Cameron or Camerons or Cape Verde or Central African Republic or Chad or China or Colombia or Comoros or Comoro Islands or Comores or Mayotte or Congo or Zaire or Costa Rica or Cote d'Ivoire or Ivory Coast or Cuba or Djibouti or French Somaliland or Dominica or Dominican Republic or East Timor or East Timur or Timor Leste or Ecuador or Egypt or United Arab Republic or El Salvador or Eritrea or Ethiopia or Fiji or Gabon or Gabonese Republic or Gambia or Gaza or Georgia Republic or Georgian Republic or Ghana or Gold Coast or Grenada or Guatemala or Guinea or Guinea-Bisau or Guam or Guiana or Guyana or Haiti or Honduras or India or Maldives or Indonesia or Iran or Iraq or Jamaica or Jordan or Kazakhstan or Kazakh or Kenya or Kiribati or Korea or Kosovo or Kyrgyzstan or Kirghizia or Kyrgyz Republic or Kirghiz or Kirgizstan or Lao PDR or Laos or Lebanon or Lesotho or Basutoland or Liberia or Libya or Macedonia or Madagascar or Malagasy Republic or Malaysia or Malaya or Malay or Sabah or Sarawak or Malawi or Nyasaland or Mali or Marshall Islands or Mauritania or Mauritius or Agalega Islands or Mexico or Micronesia or Middle East or Moldova or Moldovia or Moldovian or Mongolia or Montenegro or Morocco or Ifni or Mozambique or Myanmar or Myanma or Burma or Namibia or Nepal or Netherlands Antilles or Nicaragua or Niger or Nigeria or Pakistan or Palau or Palestine or Panama or Papua New Guinea or Paraguay or Peru or Philippines or Philipines or Phillipines or Phillippines or Romania or Rumania or Roumania or Rwanda or Ruanda or Saint Lucia or St Lucia or Saint Vincent or St Vincent or Grenadines or Samoa or Samoan Islands or Navigator Island or Navigator Islands or Sao Tome or Senegal or Serbia or Sierra Leone or Sri Lanka or Ceylon or Solomon Islands or Somalia or Sudan or Suriname or Surinam or Swaziland or Syria or Principe or South

	Sudan or Tajikistan or Tadzhikistan or Tadjikistan or Tadzhik or Tanzania or Thailand or Timor-Leste or Togo or Togolese Republic or Tonga or Tunisia or Turkey or Turkmenistan or Turkmen or Tuvalu or Uganda or Ukraine or Uzbekistan or Uzbek or Vanuatu or New Hebrides or Vietnam or Viet Nam or West Bank or Yemen or Zambia or Zimbabwe or Rhodesia) OR AB (Afghanistan or Albania or Algeria or Angola or American Samoa or Armenia or Armenian or Azerbaijan or Bangladesh or Benin or Byelarus or Byelorussian or Belarus or Belorussian or Belorussia or Belize or Bhutan or Bolivia or Bosnia or Herzegovina or Hercegovina or Botswana or Brazil or Brasil or Bulgaria or Burkina Faso or Burkina Fasso or Upper Volta or Burundi or Urundi or Cambodia or Khmer Republic or Kampuchea or Cameroon or Cameroons or Cameron or Camerons or Cape Verde or Central African Republic or Chad or China or Colombia or Comoros or Comoro Islands or Comores or Mayotte or Congo or Zaire or Costa Rica or Cote d'Ivoire or Ivory Coast or Cuba or Djibouti or French Somaliland or Dominica or Dominican Republic or East Timor or East Timur or Timor Leste or Ecuador or Egypt or United Arab Republic or El Salvador or Eritrea or Ethiopia or Fiji or Gabon or Gabonese Republic or Gambia or Gaza or Georgia Republic or Georgian Republic or Ghana or Gold Coast or Grenada or Guatemala or Guinea or Guinea-Bisau or Guam or Guiana or Guyana or Haiti or Honduras or India or Maldives or Indonesia or Iran or Iraq or Jamaica or Jordan or Kazakhstan or Kazakh or Kenya or Kiribati or Korea or Kosovo or Kyrgyzstan or Kirghizia or Kyrgyz Republic or Kirghiz or Kirgizstan or Lao PDR or Laos or Lebanon or Lesotho or Basutoland or Liberia or Libya or Macedonia or Madagascar or Malagasy Republic or Malaysia or Malaya or Malay or Sabah or Sarawak or Malawi or Nyasaland or Mali or Marshall Islands or Mauritania or Mauritius or Agalega Islands or Mexico or Micronesia or Middle East or Moldova or Moldovia or Moldovian or Mongolia or Montenegro or Morocco or Ifni or Mozambique or Myanmar or Myanma or Burma or Namibia or Nepal or Netherlands Antilles or Nicaragua or Niger or Nigeria or Pakistan or Palau or Palestine or Panama or Papua New Guinea or Paraguay or Peru or Philippines or Philipines or Phillipines or Phillippines or Romania or Rumania or Roumania or Rwanda or Ruanda or Saint Lucia or St Lucia or Saint Vincent or St Vincent or Grenadines or Samoa or Samoan Islands or Navigator Island or Navigator Islands or Sao Tome or Senegal or Serbia or Sierra Leone or Sri Lanka or Ceylon or Solomon Islands or Somalia or Sudan or Suriname or Surinam or Swaziland or Syria or Principe or South Sudan or Tajikistan or Tadzhikistan or Tadjikistan or Tadzhik or Tanzania or Thailand or Timor-Leste or Togo or Togolese Republic or Tonga or Tunisia or Turkey or Turkmenistan or Turkmen or Tuvalu or Uganda or Ukraine or Uzbekistan or Uzbek or Vanuatu or New Hebrides or Vietnam or Viet Nam or West Bank or Yemen or Zambia or Zimbabwe or Rhodesia) OR MW (Afghanistan or Albania or Algeria or Angola or American Samoa or Armenia or Armenian or Azerbaijan or Bangladesh or Benin or Byelarus or Byelorussian or Belarus or Belorussian or Belorussia or Belize or Bhutan or Bolivia or Bosnia or Herzegovina or Hercegovina or Botswana or Brazil or Brasil or Bulgaria or Burkina Faso or Burkina Fasso or Upper Volta or Burundi or Urundi or Cambodia or Khmer Republic or Kampuchea or Cameroon or Cameroons or Cameron or Camerons or Cape Verde or Central African Republic or Chad or China or Colombia or Comoros or Comoro Islands or Comores or Mayotte or Congo or Zaire or Costa Rica or Cote d'Ivoire or Ivory Coast or Cuba or Djibouti or French Somaliland or Dominica or Dominican Republic or East Timor or East Timur or Timor Leste or Ecuador or Egypt or United Arab Republic or El Salvador or Eritrea or Ethiopia or Fiji or Gabon or Gabonese Republic or Gambia or Gaza or Georgia Republic or Georgian Republic or Ghana or Gold Coast or Grenada or Guatemala or Guinea or Guinea-Bisau or Guam or Guiana or Guyana or Haiti or Honduras or India or Maldives or Indonesia or Iran or Iraq or Jamaica or Jordan or Kazakhstan or Kazakh or Kenya or Kiribati or Korea or Kosovo or Kyrgyzstan or Kirghizia or Kyrgyz Republic or Kirghiz or Kirgizstan or Lao PDR or Laos or Lebanon or Lesotho or Basutoland

	or Liberia or Libya or Macedonia or Madagascar or Malagasy Republic or Malaysia or Malaya or Malay or Sabah or Sarawak or Malawi or Nyasaland or Mali or Marshall Islands or Mauritania or Mauritius or Agalega Islands or Mexico or Micronesia or Middle East or Moldova or Moldovia or Moldovian or Mongolia or Montenegro or Morocco or Ifni or Mozambique or Myanmar or Myanma or Burma or Namibia or Nepal or Netherlands Antilles or Nicaragua or Niger or Nigeria or Pakistan or Palau or Palestine or Panama or Papua New Guinea or Paraguay or Peru or Philippines or Philipines or Phillipines or Phillippines or Romania or Rumania or Roumania or Rwanda or Ruanda or Saint Lucia or St Lucia or Saint Vincent or St Vincent or Grenadines or Samoa or Samoan Islands or Navigator Island or Navigator Islands or Sao Tome or Senegal or Serbia or Sierra Leone or Sri Lanka or Ceylon or Solomon Islands or Somalia or Sudan or Suriname or Surinam or Swaziland or Syria or Principe or South Sudan or Tajikistan or Tadzhikistan or Tadjikistan or Tadzhik or Tanzania or Thailand or Timor-Leste or Togo or Togolese Republic or Tonga or Tunisia or Turkey or Turkmenistan or Turkmen or Tuvalu or Uganda or Ukraine or Uzbekistan or Uzbek or Vanuatu or New Hebrides or Vietnam or Viet Nam or West Bank or Yemen or Zambia or Zimbabwe or Rhodesia) OR AF (Afghanistan or Albania or Algeria or Angola or American Samoa or Armenia or Armenian or Azerbaijan or Bangladesh or Benin or Byelarus or Byelorussian or Belarus or Belorussian or Belorussia or Belize or Bhutan or Bolivia or Bosnia or Herzegovina or Hercegovina or Botswana or Brazil or Brasil or Bulgaria or Burkina Faso or Burkina Fasso or Upper Volta or Burundi or Urundi or Cambodia or Khmer Republic or Kampuchea or Cameroon or Cameroons or Cameron or Camerons or Cape Verde or Central African Republic or Chad or China or Colombia or Comoros or Comoro Islands or Comores or Mayotte or Congo or Zaire or Costa Rica or Cote d'Ivoire or Ivory Coast or Cuba or Djibouti or French Somaliland or Dominica or Dominican Republic or East Timor or East Timur or Timor Leste or Ecuador or Egypt or United Arab Republic or El Salvador or Eritrea or Ethiopia or Fiji or Gabon or Gabonese Republic or Gambia or Gaza or Georgia Republic or Georgian Republic or Ghana or Gold Coast or Grenada or Guatemala or Guinea or Guinea-Bisau or Guam or Guiana or Guyana or Haiti or Honduras or India or Maldives or Indonesia or Iran or Iraq or Jamaica or Jordan or Kazakhstan or Kazakh or Kenya or Kiribati or Korea or Kosovo or Kyrgyzstan or Kirghizia or Kyrgyz Republic or Kirghiz or Kirgizstan or Lao PDR or Laos or Lebanon or Lesotho or Basutoland or Liberia or Libya or Macedonia or Madagascar or Malagasy Republic or Malaysia or Malaya or Malay or Sabah or Sarawak or Malawi or Nyasaland or Mali or Marshall Islands or Mauritania or Mauritius or Agalega Islands or Mexico or Micronesia or Middle East or Moldova or Moldovia or Moldovian or Mongolia or Montenegro or Morocco or Ifni or Mozambique or Myanmar or Myanma or Burma or Namibia or Nepal or Netherlands Antilles or Nicaragua or Niger or Nigeria or Pakistan or Palau or Palestine or Panama or Papua New Guinea or Paraguay or Peru or Philippines or Philipines or Phillipines or Phillippines or Romania or Rumania or Roumania or Rwanda or Ruanda or Saint Lucia or St Lucia or Saint Vincent or St Vincent or Grenadines or Samoa or Samoan Islands or Navigator Island or Navigator Islands or Sao Tome or Senegal or Serbia or Sierra Leone or Sri Lanka or Ceylon or Solomon Islands or Somalia or Sudan or Suriname or Surinam or Swaziland or Syria or Principe or South Sudan or Tajikistan or Tadzhikistan or Tadjikistan or Tadzhik or Tanzania or Thailand or Timor-Leste or Togo or Togolese Republic or Tonga or Tunisia or Turkey or Turkmenistan or Turkmen or Tuvalu or Uganda or Ukraine or Uzbekistan or Uzbek or Vanuatu or New Hebrides or Vietnam or Viet Nam or West Bank or Yemen or Zambia or Zimbabwe or Rhodesia)

14	(((developing or less* developed or under developed or underdeveloped or middle income or low* income or underserved or under served or deprived or poor*) N1 (countr* or nation? or state? or population? or world))) OR (((developing or less* developed or under developed or underdeveloped or middle income or low* income) N1 (economy or economies))) OR ((low* N1 (gdp or gnp or gross domestic or gross national))) OR (low N3 middle N3 countr*) OR (lmic or lmics or third world or lami countr*) OR transitional countr*
15	S12 OR S13 OR S14
16	S4 AND S11 AND S15

Cochrane Central Register of Controlled Trials

ID	Search
#1	MeSH descriptor: [Anemia, Iron-Deficiency] explode all trees
#2	MeSH descriptor: [Anemia] this term only
#3	MeSH descriptor: [Anemia, Hypochromic] this term only
#4	anaemia or anemia:ti (Word variations have been searched)
#5	((iron deficien* or hypochromic or microcytic) near (anaemia or anemia)):ti,ab,kw (Word variations have been searched)
#6	((pregnan* or gestation* or maternal) and (anaemia or anemia)):ti,ab,kw (Word variations have been searched)
#7	#1 or #2 or #3 or #4 or #5 or #6
#8	MeSH descriptor: [Labor, Obstetric] explode all trees
#9	MeSH descriptor: [Parturition] explode all trees
#10	MeSH descriptor: [Delivery, Obstetric] explode all trees
#11	labor or labour or birth? or childbirth? or delivery or obstetric*:ti (Word variations have been searched)
#12	((induction? or induced or inducing) near (labor or labour or birth? or childbirth? or delivery)):ti,ab,kw (Word variations have been searched)
#13	(augment* near (labor or labour or birth? or childbirth? or delivery)):ti,ab,kw (Word variations have been searched)
#14	(manage* near (labor or labour or birth? or childbirth? or delivery)):ti,ab,kw (Word variations have been searched)
#15	(initiat* near (labor or labour or birth? or childbirth? or delivery)):ti,ab,kw (Word variations have been searched)
#16	MeSH descriptor: [Oxytocin] explode all trees
#17	MeSH descriptor: [Prostaglandins E, Synthetic] explode all trees
#18	(uterotonic? or oxytocin or prostaglandin? or misoprostol):ti,ab,kw (Word variations have been searched)
#19	#8 or #9 or #10 or #11 or #12 or #13 or #14 or #15 or #16 or #17 or #18
#20	#7 and #19

Global Health Library

(anaemia OR anemia) AND (labour OR labor OR birth OR childbirth OR delivery OR obstetrics OR obstetric OR uterotonic OR uterotonics OR oxytocin OR prostaglandin OR prostaglandins OR misoprostol)
(iron deficiency anaemia OR iron deficiency anemia) AND (labour OR labor OR birth OR childbirth OR delivery OR obstetrics OR obstetric OR uterotonic OR uterotonics OR oxytocin OR prostaglandin OR prostaglandins OR misoprostol)
(hypochromic anaemia OR hypochromic anemia) AND (labour OR labor OR birth OR childbirth OR delivery OR obstetrics OR obstetric OR uterotonic OR uterotonics OR oxytocin OR prostaglandin OR prostaglandins OR misoprostol)
(microcytic anaemia OR microcytic anemia) AND (labour OR labor OR birth OR childbirth OR delivery OR obstetrics OR obstetric OR uterotonic OR uterotonics OR oxytocin OR prostaglandin OR prostaglandins OR misoprostol)

Dissertations & Theses (Global full text plus UK and Ireland abstracts)

Set	Search
S8	S1 AND S6
S6	ti(pregnancy OR pregnant)
S5	S1 AND S4
S4	S2 OR S3
S3	ti(uterotonic? or oxytocin or prostaglandin? or misoprostol)
S2	ti(labor or labour or birth? or childbirth? or delivery or obstetric*)
S1	ti(anaemia OR anemia)

Trials registers: Clinical trials.gov (https://clinicaltrials.gov/) and WHO ICTRP (http://apps.who.int/trialsearch/)

**Clinicaltrials.gov**
Condition=anaemia OR anemia AND Intervention=labour OR labor OR birth OR childbirth OR delivery OR obstetric OR obstetrics OR oxytocin OR prostaglandin OR prostaglandins OR misoprostol
Condition=anaemia OR anemia AND Title=labour OR labor OR birth OR childbirth OR delivery OR obstetric OR obstetrics OR oxytocin OR prostaglandin OR prostaglandins OR misoprostol
Condition=anaemia OR anemia AND Title=labour OR labor OR birth OR childbirth OR delivery OR obstetric OR obstetrics OR oxytocin OR prostaglandin OR prostaglandins OR misoprostol

**WHO Search:**
Condition=anaemia OR anemia AND Intervention=labour OR labor OR birth OR childbirth OR delivery OR obstetric OR obstetrics OR oxytocin OR prostaglandin OR prostaglandins OR misoprostol
Title=(anaemia OR anemia) AND Intervention=(labour OR labor OR birth OR childbirth OR delivery OR obstetric OR obstetrics OR oxytocin OR prostaglandin OR prostaglandins OR misoprostol)
Condition=anaemia OR anemia AND Title=labour OR labor OR birth OR childbirth OR delivery OR obstetric OR obstetrics OR oxytocin OR prostaglandin OR prostaglandins OR misoprostol
