# Supplementary material for: Systematic review to investigate the safety of induction and augmentation of labour among pregnant women with iron-deficiency anaemia
Source: BMJ Open. 2018 Dec 19;8(12):e021793. doi: 10.1136/bmjopen-2018-021793 (PMC6303660; doi:10.1136/bmjopen-2018-021793)
Supplement: Author's manuscript [file bmjopen-2018-021793.draft_revisions.pdf]

BMJ Open is committed to open peer review. As part of this commitment we make the peer review history of every article we publish publicly available.

When an article is published we post the peer reviewers' comments and the authors' responses online. We also post the versions of the paper that were used during peer review. These are the versions that the peer review comments apply to.

The versions of the paper that follow are the versions that were submitted during the peer review process. They are not the versions of record or the final published versions. They should not be cited or distributed as the published version of this manuscript.

BMJ Open is an open access journal and the full, final, typeset and author-corrected version of record of the manuscript is available on our site with no access controls, subscription charges or pay-per-view fees (http://bmjopen.bmj.com).

If you have any questions on BMJ Open's open peer review process please email info.bmjopen@bmj.com

BMJ Open

A systematic review to investigate management of labour and delivery among pregnant women with iron deficiency anaemia

Journal:	*BMJ Open*
Manuscript ID	bmjopen-2018-021793
Article Type:	Research
Date Submitted by the Author:	17-Jan-2018
Complete List of Authors:	Bunch, Kathryn; University of Oxford, National Perinatal Epidemiology Unit, Nuffield Department of Population Health<br>Roberts, Nia; University of Oxford, UK, Bodleian Health Care Libraries,<br>Knight, Marian; National Perinatal Epidemiology Unit<br>Nair, Manisha; University of Oxford, NPEU, Nuffield Department of Population Health
Keywords:	Pregnant women, Anaemia < HAEMATOLOGY, induction of labour, augmentation of labour, post-partum haemorrhage

SCHOLARONE™
Manuscripts

For peer review only

TITLE PAGE

A systematic review to investigate management of labour and delivery among pregnant women with iron deficiency anaemia

AUTHORS

1. Kathryn Bunch
   National Perinatal Epidemiology Unit, Nuffield Department of Population Health, University of Oxford, Richard Doll Building, Old Road Campus, Headington, Oxford, OX3 7LF, UK. Email: kathryn.bunch@npeu.ox.ac.uk
2. Nia Roberts
   Bodleian Libraries, University of Oxford, Knowledge Centre, Old Road Research Building, off Roosevelt Drive, Headington, Oxford, OX3 7DQ, UK.
   Email: nia.roberts@bodleian.ox.ac.uk
3. Marian Knight
   National Perinatal Epidemiology Unit, Nuffield Department of Population Health, University of Oxford, Richard Doll Building, Old Road Campus, Headington, Oxford, OX3 7LF, UK. Email: marian.knight@npeu.ox.ac.uk
4. Manisha Nair
   National Perinatal Epidemiology Unit, Nuffield Department of Population Health, University of Oxford, Richard Doll Building, Old Road Campus, Headington, Oxford, OX3 7LF, UK. Email: manisha.nair@npeu.ox.ac.uk

Corresponding author

Manisha Nair
National Perinatal Epidemiology Unit, Nuffield Department of Population Health,
University of Oxford, Richard Doll Building, Old Road Campus, Headington, Oxford, OX3 7LF, UK.
Email: manisha.nair@npeu.ox.ac.uk
Phone: +44 (0)1865617820

For peer review only - http://bmjopen.bmj.com/site/about/guidelines.xhtml

ABSTRACT

Objective: Our objective was to conduct a systematic review to investigate the management of labour and delivery among pregnant women with iron-deficiency anaemia.

Design: Systematic review.

Setting: Studies from all countries, worldwide.

Population: Pregnant women with iron-deficiency anaemia at labour and delivery.

Intervention: Any intervention related to induction and/ or augmentation of labour.

Outcome measures: Primary: Postpartum haemorrhage (PPH), heart failure and maternal death. Secondary: Emergency caesarean section, hysterectomy, admission to intensive care unit.

Method: We searched 10 databases, including Medline and Embase, from database inception to 07 November 2017. We included all study designs except cross-sectional studies without a comparator group, case reports, case series, ecological studies, and expert opinion. The searches were conducted by a healthcare librarian and two authors independently screened and reviewed the studies. We used the GRADE approach to ascertain risk of bias and conducted a narrative synthesis.

Results: We identified 2733 journal articles, 223 conference papers, 45 dissertations, and 218 registered trials. Ten articles were included for full-text review and only one was found to fulfil the eligibility criteria. This was a retrospective cohort study from India which showed that pregnant women with moderate and severe anaemia could have an increased risk of PPH if they underwent induction and/or augmentation of labour, but the evidence was weak (graded as 'High risk of bias').

Conclusion: The best approach is to prevent anaemia, but a large number of women in low-to-middle-income countries present with severe anaemia during labour. In such women, appropriate peripartum management could prevent complications and death. Our review showed that at present we do not know if induction and augmentation of labour is safe in pregnant women with iron deficiency anaemia and further research is required.

ARTICLE SUMMARY

Strengths and limitations of this study

- This is the first systematic review conducted to examine the evidence of the safety of induction and augmentation of labour in pregnant women with iron-deficiency anaemia.
- A rigorous search method was used to identify published and unpublished studies in any language from all countries, globally, using 10 databases from their inception till date.
- Despite a rigorous search over a period of two years, only one study was found.

For peer review only - http://bmjopen.bmj.com/site/about/guidelines.xhtml

For peer review only

Key words: Pregnant women; anaemia; iron-deficiency; induction of labour; augmentation of labour; post-partum haemorrhage.

Word count: 2706

For peer review only

INTRODUCTION

Iron-deficiency anaemia during pregnancy is defined as a haemoglobin concentration less than 11g/dl (110g/l) and is a major cause of maternal morbidity and mortality in low and middle income countries (LMICs)[1]. An estimated 42% of pregnant women have anaemia, globally[1], but the prevalence is reported to be as high as 70-80% in countries such as India[2]. Pregnant women with iron deficiency anaemia are at a higher risk of postpartum haemorrhage (PPH)[3] and heart failure[4] during pregnancy and childbirth. Clearly the ideal approach would be to prevent anaemia or treat it during the antenatal period, but a large number of pregnant women in LMICs present with severe anaemia either at the point of delivery or in the late third trimester of pregnancy[5 6] when there is little scope for prevention or iron treatment.

A systematic review showed that active management of the third stage of labour reduces the risk of PPH among pregnant women with haemoglobin concentrations <9g/dl, although the quality of evidence was low[7]. There is no clear evidence about the risks and benefits of induction and augmentation of labour during the first and second stages of labour among pregnant women with iron deficiency anaemia. A recent study showed that pregnant women with moderate-severe anaemia (haemoglobin <10g/dl) who underwent induction of labour were at an increased risk of PPH[8]. A few small studies have suggested that women with severe anaemia are more likely to have uterine atony due to impaired transport of oxygen rich haemoglobin to the uterus[3 9], but there is no strong evidence. This impaired transport of oxygen due to low haemoglobin could also result in early fatigue of the uterus following use of uterotonics during induction[10 11] and augmentation of labour[12] resulting in uterine atony and subsequent PPH. The aim of this systematic review was to investigate how pregnant women with iron deficiency anaemia are clinically managed during labour and delivery in order to assess the safety of induction and augmentation of labour in this specific group of pregnant women.

For peer review only - http://bmjopen.bmj.com/site/about/guidelines.xhtml

METHOD

Study design: Systematic review of published and unpublished literature. The study was registered in PROSPERO (International prospective register of systematic reviews): CRD42015032421.

Eligibility criteria

Types of studies: We included all comparative study designs. Cross-sectional studies without a comparator group, case reports, case series, ecological studies, and expert opinion pieces were excluded.

Types of participant: Our search was restricted to pregnant women with iron deficiency anaemia >20 weeks gestation at labour and delivery. We excluded pregnant women with anaemia due to haemoglobinopathies.

Setting: We included studies from all countries.

We searched for published and unpublished literature in any language from the inception of the databases until 07 November 2017.

Information sources

The following electronic databases were searched for published literature: Medline, Embase, CINAHL (Cumulative Index to Nursing and Allied Health Literature), Global Health (OvidSP), Global Health Library, and Cochrane Central Register of Controlled Trials. Unpublished or grey literature searches were restricted to dissertations, reports and conference proceedings using two recognized databases: 'Index to theses' and 'Proquest Dissertations & Theses'. In addition, we looked for ongoing unpublished trials by searching trial registers such as www.ClinicalTrials.gov and the World Health Organisation (WHO) International Clinical Trials Registry Platform (www.who.int/trialsearch/). We also planned to hand search the reference lists of included studies.

For peer review only - http://bmjopen.bmj.com/site/about/guidelines.xhtml

Search strategy

NR carried out the electronic database searches. An EndNote bibliographic database was used to manage references, identify duplicates and share references among the reviewers. The screening was carried out independently by two reviewers (KB and MN) in two stages – (i) screening of titles and abstracts based on the pre-specified inclusion and exclusion criteria; (ii) screening of full-texts of the papers included during stage-1. Any disagreements on inclusion of studies were discussed and, where possible, resolved by consensus after referring to the review protocol or a third reviewer (MK). A record of decisions made for each article was maintained. The search strategy was piloted by applying the inclusion and exclusion criteria to a sample of papers to check its reliability in correctly classifying the studies. The process helped to refine the selection criteria and the search strategies (provided in the supplementary document: S1 Text). The PRISMA flow chart was used to summarise the selection process.

Types of interventions

We searched for studies that included any intervention related to clinical management of first and second stages of labour - induction of labour, augmentation of labour, and combined induction and augmentation of labour; with and without active management of the third stage in women with iron deficiency anaemia. Key definitions of interventions and outcomes are provided in Box-1.

Comparisons

Women who did not have any intervention during the first and second stages of labour.

Types of outcome measures

Adverse maternal outcomes during labour, delivery and within 24 hours of childbirth.

Primary outcomes:

For peer review only

For peer review only - http://bmjopen.bmj.com/site/about/guidelines.xhtml

- Primary postpartum haemorrhage (defined in Box-1) or requiring therapeutic uterotonics or transfusion of blood or blood products during labour or delivery up to 24 hours after delivery.
- Heart failure during labour or delivery or up to 24 hours after delivery.
- Maternal death.

Secondary outcomes:

- Emergency caesarean section.
- Hysterectomy.
- Admission to intensive care unit.

Box-1: Key definitions

- Iron deficiency anaemia among pregnant women is defined as a haemoglobin concentration of less than 11g/dl and can be categorised as mild anaemia (haemoglobin 10-10.9g/dl), moderate anaemia (haemoglobin 7-9·9 g/dl) and severe anaemia (haemoglobin <7 g/dl)[13].
- Induction of labour is defined as 'the initiation of labour by artificial means prior to its spontaneous onset at a viable gestational age, with the aim of achieving vaginal delivery in a pregnant woman with intact membranes'[14].
- Augmentation of labour is the process of stimulating the uterus to increase the frequency, duration and intensity of contractions after the onset of labour through intravenous oxytocin infusion and/ or artificial rupture of membranes[15].
- Active management of the third stage of labour is 'a prophylactic intervention composed of a package of three components or steps: 1) administration of a uterotonic, preferably oxytocin, immediately after birth of the baby; 2) controlled cord traction to deliver the placenta; and 3) massage of the uterine fundus after the placenta is delivered.' New recommendations clarify that administration of a uterotonic is the key component, the other two being optional[16].
- A maternal death is the death of a woman while pregnant or within 42 days of the end of pregnancy, irrespective of the duration and the site of the pregnancy, from any cause related to or aggravated by the pregnancy or its management, but not from accidental or incidental causes[17].
- Primary postpartum haemorrhage is defined as a pregnant women with a blood loss of 500 ml or more from the genital tract within 24 hours of giving birth[16].

For peer review only - http://bmjopen.bmj.com/site/about/guidelines.xhtml

Data collection and analysis

The data extraction forms and coding sheets were developed after reviewing a subset of sample papers followed by testing the tools in the remaining sub-set to ensure that the tools captured all relevant information[18]. Data extraction from included studies was performed independently by two reviewers (MN and KB) and we planned to measure the level of inter-reviewer agreement using a Kappa statistic (a measure of chance-corrected agreement)[19]. As with selection of studies, we planned to resolve any disagreement through consensus or through arbitration by a third reviewer (MK). We planned to note the corrections/ amendments in the data extraction forms.

Risk of bias (quality) assessment

We used the GRADE approach to ascertain risk of bias and categorise studies into 'low risk', 'high risk' and 'unclear risk', and judgement was substantiated by a text description. We planned to assess publication bias through visual inspection and by conducting tests for asymmetry of funnel plots if at least five studies were included in the meta-analysis.

Strategy for data synthesis

We proposed to use both quantitative and narrative synthesis. Depending on the presence or absence of statistical, clinical and methodological heterogeneity of the included studies, a random-effects model or a fixed-effects model would be used to generate pooled estimates of the outcome. If clinical and methodological heterogeneity are observed, sensitivity analyses would be conducted to understand their effects on the pooled estimates. We also planned to conduct subgroup analysis to ascertain if the maternal outcomes of induction of labour and/ or augmentation of labour vary according to the severity of iron deficiency anaemia among pregnant women.

RESULTS

The search of published and unpublished studies identified 1830 journal articles from LMICs and 903 articles from high income countries (HICs), 223 conference papers, 45

For peer review only - http://bmjopen.bmj.com/site/about/guidelines.xhtml

dissertations, and 218 registered trials after removing duplicates. After screening the title and abstracts of all articles, conference proceedings and dissertations against inclusion and exclusion criteria, seven journal articles were included for full-text review from LMICs and three from HICs.

Five out of the seven studies from LMICs did not meet the inclusion criteria. The studies either did not include pregnant women with anaemia or did not include any information about the interventions of interest. One study from a hospital in India examined separately the proportion of women who underwent induction of labour and the proportion who had a postpartum haemorrhage among a total of 447 pregnant women with varying degrees of anaemia [9]. The study showed that a higher proportion of women who had severe anaemia underwent induction of labour compared with women who did not have anaemia[9]. The proportion of women who had a postpartum haemorrhage was also higher in the severe anaemia group[9]. We contacted the authors for data on postpartum haemorrhage sub-divided by induction of labour versus spontaneous vaginal delivery for each group of women with varying degrees of anaemia, but were unable to get any response despite three attempts.

The only study found to fulfil the inclusion and exclusion criteria was our previous study that led to the hypothesis for this systematic review. This was a retrospective cohort study among 1,007 pregnant women from five teaching hospitals in India. It showed that compared with women who have a normal haemoglobin concentration, women who have moderate-severe anaemia have a significantly higher odds of postpartum haemorrhage and notably, the odds increased 17-fold among women with moderate and severe anaemia who underwent induction of labour[8]. However, the evidence from the study was not strong due to a ‘high risk’ of bias assessed using the GRADE classification. Among the three studies from HICs, one was published in Bulgarian and two in Polish, but none were found to meet the inclusion criteria. The reasons for exclusion are shown in Table-1 and a PRISMA flow-chart showing the selection process is presented in Figure-1.

For peer review only - http://bmjopen.bmj.com/site/about/guidelines.xhtml

Table-1: Reasons for exclusion of studies after full-text review

Study reference	Reasons for exclusion
**Studies from LMICs**	
Tee ES, Kandiah M, Ali J, et al. (1984)[20]	This was a descriptive study of the prevalence and outcomes of nutritional anaemia among 309 pregnant women in a maternity hospital in Malaysia. Thus it did not fit the inclusion criteria for study design, interventions and outcomes.
Tsu VD. (1993)[21]	Pregnant women who were induced with oxytocin before delivery were excluded, hence no intervention or comparator.
Phillip H, Fletcher H, Reid M. (2004)[22]	Pregnant women with anaemia were excluded from the study.
Kavle JA, Stoltzfus RJ, Witter F, et al.(2008)[3]	Did not include any information on the intervention of interest – induction and/ or augmentation of labour compared with spontaneous vaginal delivery. The study showed that ergometrine and/ or oxytocins in the third stage of labour were significantly associated with increased odds of postpartum haemorrhage.
Wang X, Tan H, Zhou S, et al (2014)[23]	This was a case-control study that examined the risk factors for PPH. Anaemia was found to be an independent risk factors for PPH, but management of labour and delivery was not examined.
**Studies from HICs**	
Doncev V (1974)[24]	This study compared the proportions of maternal and fetal complications between 138 pregnant women with anaemia and 300 pregnant women without anaemia. Higher proportions of women with anaemia had preterm delivery, prolonged labour, uterine inertia, and birth asphyxia compared with women with no anaemia. The study did not fit the inclusion criterion for interventions.
Salwa M (1975)[25]	This study examined complications during labour and delivery in 309 pregnant women and reported a higher prevalence of PPH in women with anaemia (11-75%) compared with women with no anaemia (8.2%). The study did not fit the inclusion criterion for interventions.
Daraz B (1978)[26]	This cross-sectional study of 2128 pregnant women during labour showed that the average duration of the first and second stages of labour increased with a decrease in the concentration of haemoglobin, but the association was statistically significant for multiparous women only. The study did not fit the inclusion criteria for study design, interventions and outcomes.

LMICs–Low and middle income countries; HICs–High income countries; PPH–Postpartum haemorrhage

For peer review only - http://bmjopen.bmj.com/site/about/guidelines.xhtml

DISCUSSION

This systematic search of published and unpublished studies in 10 databases using key words and search criteria found only one study which showed that pregnant women with moderate and severe anaemia had an increased risk of postpartum haemorrhage (PPH) if they underwent induction and/or augmentation of labour, but the evidence was not strong. We did not find any other study that examined the clinical management of anaemic pregnant women during labour and delivery (induction and/or augmentation of labour) and its association with adverse maternal outcomes (PPH, cardiac failure, maternal death, emergency caesarean section, hysterectomy, or admission to intensive care unit).

This is the first systematic review conducted to examine the evidence of the safety of induction and augmentation of labour in pregnant women with iron-deficiency anaemia. A broad rigorous search of published and unpublished studies in any language from all countries, globally, using 10 databases from their inception till date, identified only one study that fulfilled the eligibility criteria. The results of this review are therefore inconclusive. Despite several attempts we could not obtain any further information for another study from India.

We found several studies that looked at the prevalence and outcomes of anaemia in pregnant women. The outcomes investigated were mainly PPH[27-30], low birthweight infants[31 32], preterm birth[32-38], and stillbirth[39]. Other studies examined the effects of iron supplementation and treatment of iron deficiency anaemia during the antenatal period on infant outcomes[40]. A number of studies compared the effects of different types of treatment for iron deficiency anaemia in pregnant and postpartum women for example, intravenous iron sucrose compared with oral iron[41-45], and we found a further 10 ongoing registered clinical trials examining different doses of oral iron for prevention of iron deficiency anaemia during pregnancy and different routes of iron administration to treat anaemia in pregnant women.

For peer review only - http://bmjopen.bmj.com/site/about/guidelines.xhtml

For peer review only

While some studies investigating the risk factors for PPH found that induction of labour[46 47], prolonged second stage of labour[47] and anaemia[47] were independently associated with PPH, others suggest that anaemia is associated with prolonged labour which in turn increases the risk of PPH[3 9]. A recent study from Egypt showed that compared with women who have normal haemoglobin or mild anaemia, pregnant women with moderate to severe anaemia have a higher level of nitric oxide which signals smooth muscle relaxation leading to uterine atony in the presence of tissue hypoxia[48], which can be further aggravated by uterotonics used for induction and augmentation of labour[10-12], resulting in PPH.

A prospective cohort study of about 11,000 pregnant women with moderate and severe anaemia is currently being undertaken in India through the Maternal and perinatal Health Research Collaboration, India (MaatHRI) to examine the safety of induction and augmentation of labour in this group[49]. This study will also investigate the effect of different methods of induction (mechanical using catheters and hygroscopic dilators or pharmacological using intravenous oxytocin and/ or intravaginal prostaglandins or misoprostol) and augmentation of labour (intravenous oxytocin infusion and/ or artificial rupture of membranes) on the risk of PPH and if active management of the third stage of labour modifies the risk. If an increased risk is demonstrated, there could be a potential role of the anti-fibrinolytic drug, tranexamic acid (TXA)[50], in preventing PPH in pregnant women with anaemia who undergo induction and/ or augmentation of labour, but we need further studies to find an effective dose of TXA that could prevent haemorrhage in pregnant women with moderate and severe anaemia.

For peer review only

Conclusion

This review gives no clear evidence regarding management of labour in pregnant women with iron deficiency anaemia. Studies suggest that pregnant women with anaemia are more likely to have sluggish uterine contractions[9 48] leading to prolonged second stage of labour and uterine atony[9 23] and consequently PPH. Despite several interventions such as routine

For peer review only - http://bmjopen.bmj.com/site/about/guidelines.xhtml

iron supplementation during the antenatal period, there has been the best a very small decline in the prevalence of anaemia among pregnant women globally since 1995 [51]. A large number of pregnant women present with anaemia late in pregnancy or during labour. In such women appropriate peripartum care could prevent severe complications and death. The WHO provides guidance on induction and augmentation of labour and delivery[15 52], but it does not include any information about the safety of induction and/or augmentation of labour in pregnant women with anaemia. At present, the decision to induce and/or augment labour in these women is not evidence-based and depends on the clinician's judgement. Thus, there is an urgent need to conduct further research to investigate the safety of induction and augmentation of labour in pregnant women with anaemia.

REFERENCES

1. de Benoist B, McLean E, Egli I, et al., editors. *Worldwide prevalence of anaemia 1993–2005 : WHO global database on anaemia*. Geneva: World Health Organisation, 2008.
2. Toteja GS, Singh P, Dhillon BS, et al. Prevalence of anemia among pregnant women and adolescent girls in 16 districts of India. Food and nutrition bulletin 2006;**27**(4):311-5.
3. Kavle JA, Stoltzfus RJ, Witter F, et al. Association between Anaemia during Pregnancy and Blood Loss at and after Delivery among Women with Vaginal Births in Pemba Island, Zanzibar, Tanzania. Journal of Health, Population, and Nutrition 2008;**26**(2):232-40.
4. Grewal A. Anaemia and pregnancy: Anaesthetic implications. Indian Journal of Anaesthesia 2010;**54**(5):380-86.
5. Chhabra S, Sirohi R. Trends in maternal mortality due to haemorrhage: two decades of Indian rural observations. Journal of Obstetrics & Gynecology 2004;**24**(1):40-43.
6. Rohilla M, Raveendran A, Dhaliwal L, et al. Severe anaemia in pregnancy: a tertiary hospital experience from northern India. Journal of Obstetrics & Gynaecology 2010;**30**(7):694-96.
7. Begley Cecily M, Gyte Gillian ML, Devane D, et al. Active versus expectant management for women in the third stage of labour. Cochrane Database of Systematic Reviews 2015; (3). http://onlinelibrary.wiley.com/doi/10.1002/14651858.CD007412.pub4/abstract

http://onlinelibrary.wiley.com/store/10.1002/14651858.CD007412.pub4/asset/CD007412.pdf?v=1&t=ihrpfx40&s=7958f2208cd4269e8081aed33c7a9bf798ef1221.
8. Nair M, Choudhury MK, Choudhury SS, et al. The association between maternal anaemia and pregnancy outcomes: a cohort study in Assam, India. BMJ Global Health 2016;**1**:e000026.
9. Malhotra M, Sharma JB, Batra S, et al. Maternal and perinatal outcome in varying degrees of anemia. International Journal of Gynecology & Obstetrics 2002;**79**(2):93-100.
10. Li H, Gudmundsson S, Olofsson P. Uterine artery blood flow velocity waveforms during uterine contractions. Ultrasound in obstetrics & gynecology : the official journal of the International Society of Ultrasound in Obstetrics and Gynecology 2003;**22**(6):578-85.
11. Brotanek V, Hendricks CH, Yoshida T. Changes in uterine blood flow during uterine contractions. American journal of obstetrics and gynecology 1969;**103**(8):1108-16.
12. Stanton CK, Deepak NN, Mallapur AA, et al. Direct observation of uterotonic drug use at public health facility-based deliveries in four districts in India. International journal of gynaecology

For peer review only - http://bmjopen.bmj.com/site/about/guidelines.xhtml

and obstetrics: the official organ of the International Federation of Gynaecology and Obstetrics 2014;**127**(1):25-30.

13. WHO, UNICEF, UNU. Iron deficiency anaemia: assessment, prevention and control, a guide for programme managers. 2001. http://www.who.int/nutrition/publications/micronutrients/anaemia_iron_deficiency/WHO_NHD_01.3/en/index.html (accessed 11 April 2014).
14. Eke AC, Okigbo C. Mechanical methods for induction of labour: RHL commentary (last revised: 1 August 2012) Geneva: World Health Organization: The WHO Reproductive Health Library; [cited 2015 02 December]. Available from: http://apps.who.int/rhl/pregnancy_childbirth/induction/cd001233_ekea_com/en/index.html.
15. World Health Organisation. *WHO recommendations for augmentation of labour*. Geneva: World Health Organisation, 2014.
16. World Health Organisation. *WHO recommendations for the prevention and treatment of postpartum haemorrhage*. Geneva, Switzerland World Health Organisation, 2012.
17. World Health Organisation. *The WHO application of ICD-10 to deaths during pregnancy, childbirth and puerperium: ICD MM*. Geneva World Health Organisation.
18. Brown SA, Upchurch SL, Acton GJ. A framework for developing a coding scheme for meta-analysis. Western journal of nursing research 2003;**25**(2):205-22.
19. Altman DG. Measuring agreement. In: Altman DG, ed. Practical statistics for medical research. London: Chapman and Hall, 1991.
20. Tee ES, Kandiah M, Ali J, et al. Nutritional anemia in pregnancy: a study at the maternity hospital, Kuala Lumpur. Malays J Reprod Health 1984;**2**(1):32-50.
21. Tsu VD. Postpartum haemorrhage in Zimbabwe: a risk factor analysis. Br J Obstet Gynaecol 1993;**100**(4):327-33.
22. Phillip H, Fletcher H, Reid M. The impact of induced labour on postpartum blood loss. J Obstet Gynaecol 2004;**24**(1):12-5.
23. Wang X, Tan H, Zhou S, et al. [Incidence and risk factors for postpartum hemorrhage in Liuyang, China]. Zhong Nan Da Xue Xue Bao Yi Xue Ban 2014;**Yi Xue Ban = Journal of Central South University. Medical Sciences. 39**(2):151-6.
24. Donchev V. [The effects of anemia during pregnancy on the course of labor]. Akush Ginekol (Mosk) 1974;**13**(6):421-30.
25. Salwa M. [Effect of anemia in pregnancy on labor complications]. Ginekol Pol 1975;**46**(5):513-7.
26. Daraz B. [Clinical study of labor management in women with anemia]. Ginekol Pol 1978;**49**(9):829-30.
27. Luis J, Fadel MG, Lau GY, et al. The effects of severe iron-deficiency anaemia on maternal and neonatal outcomes: A case-control study in an inner-city London hospital. Journal of Obstetrics and Gynaecology 2016;**36**(4):473-75.
28. Nagenthiran S, Ghosh M, Srinivasan M. Anaemia-a prevalent risk factor for postpartum haemorrhage. BJOG: An International Journal of Obstetrics and Gynaecology 2016;**123**:89.
29. Nyflot LT, Sandven I, Stray-Pedersen B, et al. Risk factors for severe postpartum hemorrhage: A case-control study. BMC Pregnancy and Childbirth 2017;**17 (1) (no pagination)**(17).
30. Maswime TS, Buchmann E. Near-miss maternal morbidity from severe haemorrhage at caesarean section: A process and structure audit of system deficiencies in South Africa. South African Medical Journal 2017;**107**(11):1005-09.
31. Lee S, Guillet R, Cooper EM, et al. Prevalence of anemia and associations between neonatal iron status, hepcidin, and maternal iron status among neonates born to pregnant adolescents. Pediatric Research 2016;**79(1-1)**:42-8.
32. Patabendige M, Dias T, Gunasena J, et al. Retrospective study on anaemia in pregnancy: Effect on fetal and labour outcomes. BJOG: An International Journal of Obstetrics and Gynaecology 2017;**124**:149.

For peer review only - http://bmjopen.bmj.com/site/about/guidelines.xhtml

33. Bodiazhina VI, Kravkova EV. [Some data on the course of labor and the state of newborns in anemias of pregnancy]. Sovetskaia Meditsina 1958;**22(2)**:104-9.
34. Li Z, Li H, Jin L, et al. Maternal haemoglobin concentration and risk of preterm birth in a Chinese population. Journal of Obstetrics and Gynaecology 2017:1-6.
35. Pakniat H, Soofizadeh N, Dabbaghi Ghale T. The relationship between hemoglobin level in the first trimester of pregnancy and preterm delivery. [Persian]. Scientific Journal of Kurdistan University of Medical Sciences 2016;**21**(5):60-68.
36. Radon-Pokracka M, Huras H, Spaczynska J, et al. Influence of antenatal anemia on the route of delivery and neonatal outcomes Wplyw anemii w okresie okoloporodowym na przebieg porodu i wyniki neonatologiczne. Ginekologia i Poloznictwo 2016;**41**(3):48-51, 9-12.
37. Radon-Pokracka M, Huras H, Spaczynska J, et al. Relationship between preterm birth, neonatal outcomes and low maternal hemoglobin level. Journal of Maternal-Fetal and Neonatal Medicine 2016;**29**:66.
38. Rao CR, Bhat P, Ke V, et al. Assessment of risk factors and predictors for spontaneous pre-term birth in a South Indian antenatal cohort. Clinical Epidemiology and Global Health 2017.
39. Nair M, Churchill D, Robinson S, et al. Association between maternal haemoglobin concentration and stillbirth: A cohort study among a multi-ethnic population in England. BJOG: An International Journal of Obstetrics and Gynaecology 2017;**124**:143.
40. Pinho-Pompeu M, Surita FG, Pastore DA, et al. Anemia in pregnant adolescents: impact of treatment on perinatal outcomes. Journal of Maternal-Fetal and Neonatal Medicine 2017;**30**(10):1158-62.
41. Colley C, Chaudhry U, Eissa A. The role of Iron infusion in postpartum anaemia. BJOG: An International Journal of Obstetrics and Gynaecology 2017;**124**:118-19.
42. Fan T, Blitz J. Screening for Iron Deficiency Anemia and Iron Supplementation in Pregnant Women to Improve Maternal Health and Birth Outcomes. American Family Physician 2016;**93(2)**:137-8.
43. Nash CM, Allen VM. The Use of Parenteral Iron Therapy for the Treatment of Postpartum Anemia. Journal of Obstetrics and Gynaecology Canada 2015;**37**(5):439-42.
44. Ogunbode O, Damole IO. Treatment of anaemia in obstetric patients with sustained-release (Ferrograd Folic 500 Plus) and conventional fersolate and folic acid as separate drugs. Current Therapeutic Research - Clinical and Experimental 1984;**35**(6):1038-42.
45. Qassim A, Gergis R, Jeffries B, et al. Use of intravenous iron polymaltose in the management of iron deficiency in pregnancy: A retrospective cohort study. Journal of Paediatrics and Child Health 2017;**53**:40.
46. Sheldon WR, Blum J, Vogel JP, et al. Postpartum haemorrhage management, risks, and maternal outcomes: findings from the World Health Organization Multicountry Survey on Maternal and Newborn Health. Bjog 2014;**121 Suppl 1**:5-13.
47. Wetta LA, Szychowski JM, Seals S, et al. Risk factors for uterine atony/postpartum hemorrhage requiring treatment after vaginal delivery. Am J Obstet Gynecol 2013;**209**(1):51.e1-6.
48. Soltan MH, Ibrahim EM, Tawfek M, et al. Raised nitric oxide levels may cause atonic postpartum hemorrhage in women with anemia during pregnancy. International journal of gynaecology and obstetrics: the official organ of the International Federation of Gynaecology and Obstetrics 2012;**116**(2):143-7.
49. National Perinatal Epidemiology Unit. MaatHRI: Maternal and perinatal Health Research collaboration, India Oxford: National Perinatal Epidemiology Unit, University of Oxford; 2017 [cited 2017 14 December]. Available from: https://www.npeu.ox.ac.uk/maathri.
50. Shakur H, Roberts I, Fawole B, et al. Effect of early tranexamic acid administration on mortality, hysterectomy, and other morbidities in women with post-partum haemorrhage (WOMAN): an international, randomised, double-blind, placebo-controlled trial. The Lancet 2017;**389**(10084):2105-16.

For peer review only - http://bmjopen.bmj.com/site/about/guidelines.xhtml

51. Stevens GA, Finucane MM, De-Regil LM, et al. Global, regional, and national trends in haemoglobin concentration and prevalence of total and severe anaemia in children and pregnant and non-pregnant women for 1995-2011: a systematic analysis of population-representative data. The Lancet Global Health 2014;**1**(1):e16-e25.
52. WHO. *WHO recommendations for induction of labour*. Geneva: WHO, 2011.

Supplementary file S1: Search strategies

Acknowledgement: We would like to acknowledge the help from Anna Balchan, UKOSS/BAPS-CASS Programme Administrator at the NPEU, in reading and translating the papers published in Polish.

Disclosure of interests: The authors declare that they have no competing interests.

Contribution to authorship: KB reviewed the literature, extracted information from studies and edited the paper; NR conducted the searches and edited the paper; MK was the third reviewer and edited the paper; MN developed the concept for the systematic review, reviewed the literature, extracted information from studies and wrote the first draft of the paper.

Details of ethics approval: Not applicable since this is a systematic review.

Funding: The work was funded by a Medical Research Council (MRC) Career Development Award (https://www.mrc.ac.uk/) for Manisha Nair (Grant Ref: MR/P022030/1). The funder had no role in study design, data collection, data analysis, data interpretation, or writing of the report. The corresponding author had full access to all the data in the study and had final responsibility for the decision to submit for publication.

Data sharing statement: Data sharing is not applicable to this article as no datasets were generated or analysed during the review.

Figure legend

Figure-1: PRISMA flow-chart of the selection process for the systematic review

For peer review only - http://bmjopen.bmj.com/site/about/guidelines.xhtml

Figure-1: PRISMA flow-chart of the selection process for the systematic review

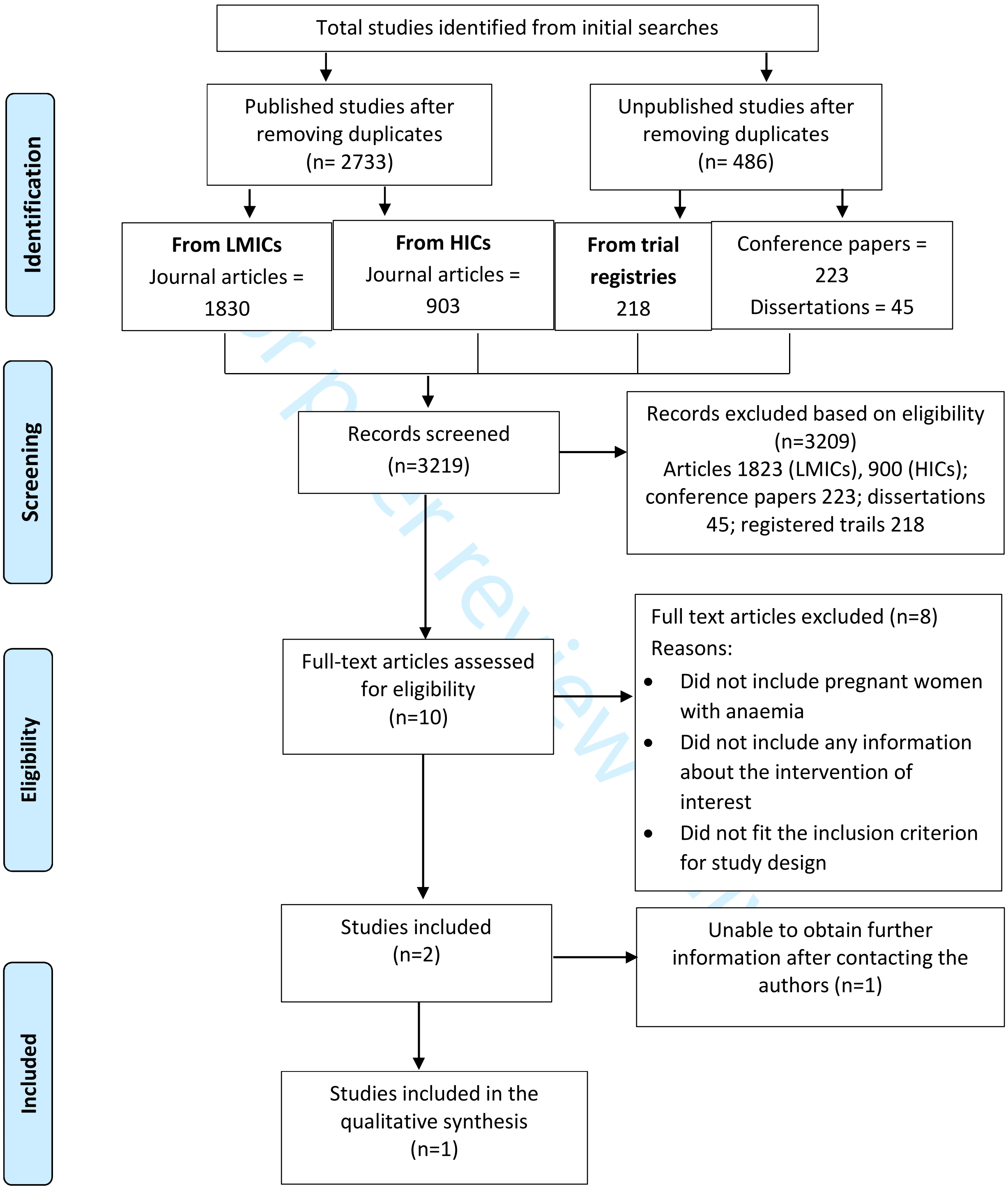

LMICs: Low-to-middle income countries; HICs: High income countries

S1 Text

Search strategies

Medline

# ▲	Searches
1	anemia/ or anemia, hypochromic/ or anemia, iron-deficiency/
2	(anaemia or anemia).ti.
3	((iron deficien* or hypochromic or microcytic) adj5 (anaemia or anemia)).ti,ab.
4	((pregnan* or gestation* or maternal) and (anaemia or anemia)).ti,ab.
5	(Pregnancy/ or Pregnant woman/) and (anaemia or anemia).mp.
6	1 or 2 or 3 or 4 or 5
7	exp Labor, Obstetric/
8	exp Parturition/
9	exp Delivery, Obstetric/
10	(labor or labour or birth? or childbirth? or delivery or obstetric*).ti.
11	((induction? or induced or inducing) adj5 (labor or labour or birth? or childbirth? or delivery)).ti,ab.
12	(augment* adj5 (labor or labour or birth? or childbirth? or delivery)).ti,ab.
13	(manage* adj5 (labor or labour or birth? or childbirth? or delivery)).ti,ab.
14	(initiat* adj5 (labor or labour or birth? or childbirth? or delivery)).ti,ab.
15	Oxytocin/
16	exp Prostaglandins E, Synthetic/
17	(uterotonic? or oxytocin or prostaglandin? or misoprostol).ti,ab.
18	7 or 8 or 9 or 10 or 11 or 12 or 13 or 14 or 15 or 16 or 17
19	Developing Countries/
20	(Africa or Caribbean or West Indies or South America or Latin America or Central America).hw,kf,ti,ab,cp.

For peer review only

For peer review only - http://bmjopen.bmj.com/site/about/guidelines.xhtml

21	(Afghanistan or Albania or Algeria or Angola or American Samoa or Armenia or Armenian or Azerbaijan or Bangladesh or Benin or Byelarus or Byelorussian or Belarus or Belorussian or Belorussia or Belize or Bhutan or Bolivia or Bosnia or Herzegovina or Hercegovina or Botswana or Brazil or Brasil or Bulgaria or Burkina Faso or Burkina Fasso or Upper Volta or Burundi or Urundi or Cambodia or Khmer Republic or Kampuchea or Cameroon or Cameroons or Cameron or Camerons or Cape Verde or Central African Republic or Chad or China or Colombia or Comoros or Comoro Islands or Comores or Mayotte or Congo or Zaire or Costa Rica or Cote d'Ivoire or Ivory Coast or Cuba or Djibouti or French Somaliland or Dominica or Dominican Republic or East Timor or East Timur or Timor Leste or Ecuador or Egypt or United Arab Republic or El Salvador or Eritrea or Ethiopia or Fiji or Gabon or Gabonese Republic or Gambia or Gaza or Georgia Republic or Georgian Republic or Ghana or Gold Coast or Grenada or Guatemala or Guinea or Guinea-Bisau or Guam or Guiana or Guyana or Haiti or Honduras or India or Maldives or Indonesia or Iran or Iraq or Jamaica or Jordan or Kazakhstan or Kazakh or Kenya or Kiribati or Korea or Kosovo or Kyrgyzstan or Kirghizia or Kyrgyz Republic or Kirghiz or Kirgizstan or Lao PDR or Laos or Lebanon or Lesotho or Basutoland or Liberia or Libya or Macedonia or Madagascar or Malagasy Republic or Malaysia or Malaya or Malay or Sabah or Sarawak or Malawi or Nyasaland or Mali or Marshall Islands or Mauritania or Mauritius or Agalega Islands or Mexico or Micronesia or Middle East or Moldova or Moldovia or Moldovian or Mongolia or Montenegro or Morocco or Ifni or Mozambique or Myanmar or Myanma or Burma or Namibia or Nepal or Netherlands Antilles or Nicaragua or Niger or Nigeria or Pakistan or Palau or Palestine or Panama or Papua New Guinea or Paraguay or Peru or Philippines or Philipines or Phillipines or Phillippines or Romania or Rumania or Roumania or Rwanda or Ruanda or Saint Lucia or St Lucia or Saint Vincent or St Vincent or Grenadines or Samoa or Samoan Islands or Navigator Island or Navigator Islands or Sao Tome or Senegal or Serbia or Sierra Leone or Sri Lanka or Ceylon or Solomon Islands or Somalia or Sudan or Suriname or Surinam or Swaziland or Syria or Principe or South Sudan or Tajikistan or Tadzhikistan or Tadjikistan or Tadzhik or Tanzania or Thailand or Timor-Leste or Togo or Togolese Republic or Tonga or Tunisia or Turkey or Turkmenistan or Turkmen or Tuvalu or Uganda or Ukraine or Uzbekistan or Uzbek or Vanuatu or New Hebrides or Vietnam or Viet Nam or West Bank or Yemen or Zambia or Zimbabwe or Rhodesia).hw,kf,ti,ab,cp.
22	((developing or less* developed or under developed or underdeveloped or middle income or low* income or underserved or under served or deprived or poor*) adj (countr* or nation? or state? or population? or world)).ti,ab.
23	((developing or less* developed or under developed or underdeveloped or middle income or low* income) adj (economy or economies)).ti,ab.
24	(low* adj (gdp or gnp or gross domestic or gross national)).ti,ab.
25	(low adj3 middle adj3 countr*).ti,ab.
26	(lmic or lmics or third world or lami countr*).ti,ab.
27	transitional countr*.ti,ab.
28	19 or 20 or 21 or 22 or 23 or 24 or 25 or 26 or 27
29	6 and 18 and 28

For peer review only - http://bmjopen.bmj.com/site/about/guidelines.xhtml

Embase

# ▲	Searches
1	*anemia/ or iron deficiency anemia/ or microcytic anemia/
2	(anaemia or anemia).ti.
3	((iron deficien* or hypochromic or microcytic) adj5 (anaemia or anemia)).ti,ab.
4	((pregnan* or gestation* or maternal) and (anaemia or anemia)).ti,ab.
5	(Pregnancy/ or Pregnant woman/) and (anaemia or anemia).mp.
6	1 or 2 or 3 or 4 or 5
7	exp labor/
8	childbirth/
9	exp delivery/
10	(labor or labour or birth? or childbirth? or delivery or obstetric*).ti.
11	((induction? or induced or inducing) adj5 (labor or labour or birth? or childbirth? or delivery)).ti,ab.
12	(augment* adj5 (labor or labour or birth? or childbirth? or delivery)).ti,ab.
13	(manage* adj5 (labor or labour or birth? or childbirth? or delivery)).ti,ab.
14	(initiat* adj5 (labor or labour or birth? or childbirth? or delivery)).ti,ab.
15	exp uterotonic agent/
16	(uterotonic? or oxytocin or prostaglandin? or misoprostol).ti,ab.
17	7 or 8 or 9 or 10 or 11 or 12 or 13 or 14 or 15 or 16
18	Developing Country.sh.
19	(Africa or Caribbean or West Indies or South America or Latin America or Central America).hw,ti,ab,cp.

For peer review only

20	(Afghanistan or Albania or Algeria or Angola or American Samoa or Armenia or Armenian or Azerbaijan or Bangladesh or Benin or Byelarus or Byelorussian or Belarus or Belorussian or Belorussia or Belize or Bhutan or Bolivia or Bosnia or Herzegovina or Hercegovina or Botswana or Brazil or Brasil or Bulgaria or Burkina Faso or Burkina Fasso or Upper Volta or Burundi or Urundi or Cambodia or Khmer Republic or Kampuchea or Cameroon or Cameroons or Cameron or Camerons or Cape Verde or Central African Republic or Chad or China or Colombia or Comoros or Comoro Islands or Comores or Mayotte or Congo or Zaire or Costa Rica or Cote d'Ivoire or Ivory Coast or Cuba or Djibouti or French Somaliland or Dominica or Dominican Republic or East Timor or East Timur or Timor Leste or Ecuador or Egypt or United Arab Republic or El Salvador or Eritrea or Ethiopia or Fiji or Gabon or Gabonese Republic or Gambia or Gaza or Georgia Republic or Georgian Republic or Ghana or Gold Coast or Grenada or Guatemala or Guinea or Guinea-Bisau or Guam or Guiana or Guyana or Haiti or Honduras or India or Maldives or Indonesia or Iran or Iraq or Jamaica or Jordan or Kazakhstan or Kazakh or Kenya or Kiribati or Korea or Kosovo or Kyrgyzstan or Kirghizia or Kyrgyz Republic or Kirghiz or Kirgizstan or Lao PDR or Laos or Lebanon or Lesotho or Basutoland or Liberia or Libya or Macedonia or Madagascar or Malagasy Republic or Malaysia or Malaya or Malay or Sabah or Sarawak or Malawi or Nyasaland or Mali or Marshall Islands or Mauritania or Mauritius or Agalega Islands or Mexico or Micronesia or Middle East or Moldova or Moldovia or Moldovian or Mongolia or Montenegro or Morocco or Ifni or Mozambique or Myanmar or Myanma or Burma or Namibia or Nepal or Netherlands Antilles or Nicaragua or Niger or Nigeria or Pakistan or Palau or Palestine or Panama or Papua New Guinea or Paraguay or Peru or Philippines or Philipines or Phillipines or Phillippines or Romania or Rumania or Roumania or Rwanda or Ruanda or Saint Lucia or St Lucia or Saint Vincent or St Vincent or Grenadines or Samoa or Samoan Islands or Navigator Island or Navigator Islands or Sao Tome or Senegal or Serbia or Sierra Leone or Sri Lanka or Ceylon or Solomon Islands or Somalia or Sudan or Suriname or Surinam or Swaziland or Syria or Principe or South Sudan or Tajikistan or Tadzhikistan or Tadjikistan or Tadzhik or Tanzania or Thailand or Timor-Leste or Togo or Togolese Republic or Tonga or Tunisia or Turkey or Turkmenistan or Turkmen or Tuvalu or Uganda or Ukraine or Uzbekistan or Uzbek or Vanuatu or New Hebrides or Vietnam or Viet Nam or West Bank or Yemen or Zambia or Zimbabwe or Rhodesia).hw,ti,ab,cp.
21	((developing or less* developed or under developed or underdeveloped or middle income or low* income or underserved or under served or deprived or poor*) adj (countr* or nation? or state? or population? or world)).ti,ab.
22	((developing or less* developed or under developed or underdeveloped or middle income or low* income) adj (economy or economies)).ti,ab.
23	(low* adj (gdp or gnp or gross domestic or gross national)).ti,ab.
24	(low adj3 middle adj3 countr*).ti,ab.
25	(lmic or lmics or third world or lami countr*).ti,ab.
26	transitional countr*.ti,ab.
27	18 or 19 or 20 or 21 or 22 or 23 or 24 or 25 or 26
28	6 and 17 and 27

For peer review only

For peer review only - http://bmjopen.bmj.com/site/about/guidelines.xhtml

Global Health

# ▲	Searches
1	anaemia/ or hypochromic anaemia/ or iron deficiency anaemia/ or microcytic anaemia/
2	(anaemia or anemia).ti.
3	((iron deficien* or hypochrmomic or microcytic) adj5 (anaemia or anemia)).ti,ab.
4	((pregnan* or gestation* or maternal) and (anaemia or anemia)).ti,ab.
5	1 or 2 or 3 or 4
6	birth/ or childbirth/ or parturition/
7	(labor or labour or birth? or childbirth? or delivery or obstetric*).ti.
8	((induction? or induced or inducing) adj5 (labor or labour or birth? or childbirth? or delivery)).ti,ab.
9	(augment* adj5 (labor or labour or birth? or childbirth? or delivery)).ti,ab.
10	(manage* adj5 (labor or labour or birth? or childbirth? or delivery)).ti,ab.
11	oxytocin/
12	exp synthetic prostaglandins/
13	(uterotonic? or oxytocin or prostaglandin? or misoprostol).ti,ab.
14	6 or 7 or 8 or 9 or 10 or 11 or 12 or 13
15	exp developing countries/ or exp africa/
16	(Africa or Caribbean or West Indies or South America or Latin America or Central America).hw,ti,ab,cp.
17	(Afghanistan or Albania or Algeria or Angola or American Samoa or Armenia or Armenian or Azerbaijan or Bangladesh or Benin or Byelarus or Byelorussian or Belarus or Belorussian or Belorussia or Belize or Bhutan or Bolivia or Bosnia or Herzegovina or Hercegovina or Botswana or Brazil or Brasil or Bulgaria or Burkina Faso or Burkina Fasso or Upper Volta or Burundi or Urundi or Cambodia or Khmer Republic or Kampuchea or Cameroon or Cameroons or Cameron or Camerons or Cape Verde or Central African Republic or Chad or China or Colombia or Comoros or Comoro Islands or Comores or Mayotte or Congo or Zaire or Costa Rica or Cote d'Ivoire or Ivory Coast or Cuba or Djibouti or French Somaliland or Dominica or Dominican Republic or East Timor or East Timur or Timor Leste or Ecuador or Egypt or United Arab Republic or El Salvador or Eritrea or Ethiopia or Fiji or Gabon or Gabonese Republic or Gambia or Gaza or Georgia Republic or Georgian Republic or Ghana or Gold Coast or Grenada or Guatemala or Guinea or Guinea-Bisau or Guam or Guiana or Guyana or Haiti or Honduras or India or Maldives or Indonesia or Iran or Iraq or Jamaica or Jordan or Kazakhstan or Kazakh or Kenya or Kiribati or Korea or Kosovo or Kyrgyzstan or Kirghizia or Kyrgyz Republic or Kirghiz or Kirgizstan or Lao PDR or Laos or Lebanon or Lesotho or Basutoland or Liberia or Libya or Macedonia or Madagascar or Malagasy Republic or Malaysia or Malaya or Malay or Sabah or Sarawak or Malawi or Nyasaland or Mali or Marshall Islands or Mauritania or Mauritius or Agalega Islands or Mexico or Micronesia or Middle East or Moldova or Moldovia or Moldovian or Mongolia or Montenegro or Morocco or Ifni or Mozambique or Myanmar or Myanma or Burma or Namibia or Nepal or Netherlands Antilles or Nicaragua or Niger or Nigeria or Pakistan or Palau or Palestine or Panama or Papua New Guinea or Paraguay or Peru or Philippines or Philipines or Phillipines or Phillippines or Romania or Rumania or Roumania or Rwanda or Ruanda or Saint Lucia or St Lucia or Saint Vincent or St Vincent or Grenadines or Samoa or Samoan Islands or Navigator Island or Navigator Islands or Sao Tome or Senegal or Serbia or Sierra Leone or Sri Lanka or Ceylon or Solomon Islands or Somalia or Sudan or Suriname or Surinam or Swaziland or Syria or Principe or South Sudan or Tajikistan or Tadzhikistan or Tadjikistan or Tadzhik or Tanzania or Thailand or Timor-Leste or Togo or

For peer review only - http://bmjopen.bmj.com/site/about/guidelines.xhtml

	Togolese Republic or Tonga or Tunisia or Turkey or Turkmenistan or Turkmen or Tuvalu or Uganda or Ukraine or Uzbekistan or Uzbek or Vanuatu or New Hebrides or Vietnam or Viet Nam or West Bank or Yemen or Zambia or Zimbabwe or Rhodesia).hw,ti,ab,cp.
18	((developing or less* developed or under developed or underdeveloped or middle income or low* income or underserved or underserved or deprived or poor*) adj (countr* or nation? or state? or population? or world)).ti,ab.
19	((developing or less* developed or under developed or underdeveloped or middle income or low* income) adj (economy or economies)).ti,ab.
20	(low* adj (gdp or gnp or gross domestic or gross national)).ti,ab.
21	(low adj3 middle adj3 countr*).ti,ab.
22	(lmic or lmics or third world or lami countr*).ti,ab.
23	transitional countr*.ti,ab.
24	15 or 16 or 17 or 18 or 19 or 20 or 21 or 22 or 23
25	5 and 14 and 24

For peer review only

CINAHL

#	Query
1	(MH "Anemia") OR (MH "Anemia, Hypochromic") OR (MH "Anemia, Iron Deficiency")
2	TI (anaemia or anemia) OR AB (((iron deficien* or hypochromic or microcytic) N5 (anaemia or anemia))) OR AB (((pregnan* or gestation* or maternal) and (anaemia or anemia)))
3	((MH "Pregnancy") OR (MH "Expectant Mothers")) AND (anaemia or anemia)
4	S1 OR S2 OR S3
5	(MH "Childbirth+") OR (MH "Labor+")
6	(MH "Delivery, Obstetric+")
7	TI (labor or labour or birth? or childbirth? or delivery or obstetric*) OR AB (((induction? or induced or inducing) N5 (labor or labour or birth? or childbirth? or delivery))) OR AB ((augment* N5 (labor or labour or birth? or childbirth? or delivery))) OR AB ((manage* N5 (labor or labour or birth? or childbirth? or delivery))) OR AB ((initiat* N5 (labor or labour or birth? or childbirth? or delivery)))
8	(MH "Oxytocics+")
9	(MH "Prostaglandins, Synthetic+")
10	TI (uterotonic? or oxytocin or prostaglandin? or misoprostol) OR AB (uterotonic? or oxytocin or prostaglandin? or misoprostol)
11	S5 OR S6 OR S7 OR S8 OR S9 OR S10
12	(MH "Developing Countries")
13	TI (Afghanistan or Albania or Algeria or Angola or American Samoa or Armenia or Armenian or Azerbaijan or Bangladesh or Benin or Byelarus or Byelorussian or Belarus or Belorussian or Belorussia or Belize or Bhutan or Bolivia or Bosnia or Herzegovina or Hercegovina or Botswana or Brazil or Brasil or Bulgaria or Burkina Faso or Burkina Fasso or Upper Volta or Burundi or Urundi or Cambodia or Khmer Republic or Kampuchea or Cameroon or Cameroons or Cameron or Camerons or Cape Verde or Central African Republic or Chad or China or Colombia or Comoros or Comoro Islands or Comores or Mayotte or Congo or Zaire or Costa Rica or Cote d'Ivoire or Ivory Coast or Cuba or Djibouti or French Somaliland or Dominica or Dominican Republic or East Timor or East Timur or Timor Leste or Ecuador or Egypt or United Arab Republic or El Salvador or Eritrea or Ethiopia or Fiji or Gabon or Gabonese Republic or Gambia or Gaza or Georgia Republic or Georgian Republic or Ghana or Gold Coast or Grenada or Guatemala or Guinea or Guinea-Bisau or Guam or Guiana or Guyana or Haiti or Honduras or India or Maldives or Indonesia or Iran or Iraq or Jamaica or Jordan or Kazakhstan or Kazakh or Kenya or Kiribati or Korea or Kosovo or Kyrgyzstan or Kirghizia or Kyrgyz Republic or Kirghiz or Kirgizstan or Lao PDR or Laos or Lebanon or Lesotho or Basutoland or Liberia or Libya or Macedonia or Madagascar or Malagasy Republic or Malaysia or Malaya or Malay or Sabah or Sarawak or Malawi or Nyasaland or Mali or Marshall Islands or Mauritania or Mauritius or Agalega Islands or Mexico or Micronesia or Middle East or Moldova or Moldovia or Moldovian or Mongolia or Montenegro or Morocco or Ifni or Mozambique or Myanmar or Myanma or Burma or Namibia or Nepal or Netherlands Antilles or Nicaragua or Niger or Nigeria or Pakistan or Palau or Palestine or Panama or Papua New Guinea or Paraguay or Peru or Philippines or Philipines or Phillipines or Phillippines or Romania or Rumania or Roumania or Rwanda or Ruanda or Saint Lucia or St Lucia or Saint Vincent or St Vincent or Grenadines or Samoa or Samoan Islands or Navigator Island or Navigator Islands or Sao Tome or Senegal or Serbia or Sierra Leone or Sri Lanka or Ceylon or Solomon Islands or Somalia or Sudan or Suriname or Surinam or Swaziland or Syria or Principe or South

For peer review only - http://bmjopen.bmj.com/site/about/guidelines.xhtml

	Sudan or Tajikistan or Tadzhikistan or Tadjikistan or Tadzhik or Tanzania or Thailand or Timor-Leste or Togo or Togolese Republic or Tonga or Tunisia or Turkey or Turkmenistan or Turkmen or Tuvalu or Uganda or Ukraine or Uzbekistan or Uzbek or Vanuatu or New Hebrides or Vietnam or Viet Nam or West Bank or Yemen or Zambia or Zimbabwe or Rhodesia) OR AB (Afghanistan or Albania or Algeria or Angola or American Samoa or Armenia or Armenian or Azerbaijan or Bangladesh or Benin or Byelarus or Byelorussian or Belarus or Belorussian or Belorussia or Belize or Bhutan or Bolivia or Bosnia or Herzegovina or Hercegovina or Botswana or Brazil or Brasil or Bulgaria or Burkina Faso or Burkina Fasso or Upper Volta or Burundi or Urundi or Cambodia or Khmer Republic or Kampuchea or Cameroon or Cameroons or Cameron or Camerons or Cape Verde or Central African Republic or Chad or China or Colombia or Comoros or Comoro Islands or Comores or Mayotte or Congo or Zaire or Costa Rica or Cote d'Ivoire or Ivory Coast or Cuba or Djibouti or French Somaliland or Dominica or Dominican Republic or East Timor or East Timur or Timor Leste or Ecuador or Egypt or United Arab Republic or El Salvador or Eritrea or Ethiopia or Fiji or Gabon or Gabonese Republic or Gambia or Gaza or Georgia Republic or Georgian Republic or Ghana or Gold Coast or Grenada or Guatemala or Guinea or Guinea-Bisau or Guam or Guiana or Guyana or Haiti or Honduras or India or Maldives or Indonesia or Iran or Iraq or Jamaica or Jordan or Kazakhstan or Kazakh or Kenya or Kiribati or Korea or Kosovo or Kyrgyzstan or Kirghizia or Kyrgyz Republic or Kirghiz or Kirgizstan or Lao PDR or Laos or Lebanon or Lesotho or Basutoland or Liberia or Libya or Macedonia or Madagascar or Malagasy Republic or Malaysia or Malaya or Malay or Sabah or Sarawak or Malawi or Nyasaland or Mali or Marshall Islands or Mauritania or Mauritius or Agalega Islands or Mexico or Micronesia or Middle East or Moldova or Moldovia or Moldovian or Mongolia or Montenegro or Morocco or Ifni or Mozambique or Myanmar or Myanma or Burma or Namibia or Nepal or Netherlands Antilles or Nicaragua or Niger or Nigeria or Pakistan or Palau or Palestine or Panama or Papua New Guinea or Paraguay or Peru or Philippines or Philipines or Phillipines or Phillippines or Romania or Rumania or Roumania or Rwanda or Ruanda or Saint Lucia or St Lucia or Saint Vincent or St Vincent or Grenadines or Samoa or Samoan Islands or Navigator Island or Navigator Islands or Sao Tome or Senegal or Serbia or Sierra Leone or Sri Lanka or Ceylon or Solomon Islands or Somalia or Sudan or Suriname or Surinam or Swaziland or Syria or Principe or South Sudan or Tajikistan or Tadzhikistan or Tadjikistan or Tadzhik or Tanzania or Thailand or Timor-Leste or Togo or Togolese Republic or Tonga or Tunisia or Turkey or Turkmenistan or Turkmen or Tuvalu or Uganda or Ukraine or Uzbekistan or Uzbek or Vanuatu or New Hebrides or Vietnam or Viet Nam or West Bank or Yemen or Zambia or Zimbabwe or Rhodesia) OR MW (Afghanistan or Albania or Algeria or Angola or American Samoa or Armenia or Armenian or Azerbaijan or Bangladesh or Benin or Byelarus or Byelorussian or Belarus or Belorussian or Belorussia or Belize or Bhutan or Bolivia or Bosnia or Herzegovina or Hercegovina or Botswana or Brazil or Brasil or Bulgaria or Burkina Faso or Burkina Fasso or Upper Volta or Burundi or Urundi or Cambodia or Khmer Republic or Kampuchea or Cameroon or Cameroons or Cameron or Camerons or Cape Verde or Central African Republic or Chad or China or Colombia or Comoros or Comoro Islands or Comores or Mayotte or Congo or Zaire or Costa Rica or Cote d'Ivoire or Ivory Coast or Cuba or Djibouti or French Somaliland or Dominica or Dominican Republic or East Timor or East Timur or Timor Leste or Ecuador or Egypt or United Arab Republic or El Salvador or Eritrea or Ethiopia or Fiji or Gabon or Gabonese Republic or Gambia or Gaza or Georgia Republic or Georgian Republic or Ghana or Gold Coast or Grenada or Guatemala or Guinea or Guinea-Bisau or Guam or Guiana or Guyana or Haiti or Honduras or India or Maldives or Indonesia or Iran or Iraq or Jamaica or Jordan or Kazakhstan or Kazakh or Kenya or Kiribati or Korea or Kosovo or Kyrgyzstan or Kirghizia or Kyrgyz Republic or Kirghiz or Kirgizstan or Lao PDR or Laos or Lebanon or Lesotho or Basutoland

For peer review only - http://bmjopen.bmj.com/site/about/guidelines.xhtml

	or Liberia or Libya or Macedonia or Madagascar or Malagasy Republic or Malaysia or Malaya or Malay or Sabah or Sarawak or Malawi or Nyasaland or Mali or Marshall Islands or Mauritania or Mauritius or Agalega Islands or Mexico or Micronesia or Middle East or Moldova or Moldovia or Moldovian or Mongolia or Montenegro or Morocco or Ifni or Mozambique or Myanmar or Myanma or Burma or Namibia or Nepal or Netherlands Antilles or Nicaragua or Niger or Nigeria or Pakistan or Palau or Palestine or Panama or Papua New Guinea or Paraguay or Peru or Philippines or Philipines or Phillipines or Phillippines or Romania or Rumania or Roumania or Rwanda or Ruanda or Saint Lucia or St Lucia or Saint Vincent or St Vincent or Grenadines or Samoa or Samoan Islands or Navigator Island or Navigator Islands or Sao Tome or Senegal or Serbia or Sierra Leone or Sri Lanka or Ceylon or Solomon Islands or Somalia or Sudan or Suriname or Surinam or Swaziland or Syria or Principe or South Sudan or Tajikistan or Tadzhikistan or Tadjikistan or Tadzhik or Tanzania or Thailand or Timor-Leste or Togo or Togolese Republic or Tonga or Tunisia or Turkey or Turkmenistan or Turkmen or Tuvalu or Uganda or Ukraine or Uzbekistan or Uzbek or Vanuatu or New Hebrides or Vietnam or Viet Nam or West Bank or Yemen or Zambia or Zimbabwe or Rhodesia) OR AF (Afghanistan or Albania or Algeria or Angola or American Samoa or Armenia or Armenian or Azerbaijan or Bangladesh or Benin or Byelarus or Byelorussian or Belarus or Belorussian or Belorussia or Belize or Bhutan or Bolivia or Bosnia or Herzegovina or Hercegovina or Botswana or Brazil or Brasil or Bulgaria or Burkina Faso or Burkina Fasso or Upper Volta or Burundi or Urundi or Cambodia or Khmer Republic or Kampuchea or Cameroon or Cameroons or Cameron or Camerons or Cape Verde or Central African Republic or Chad or China or Colombia or Comoros or Comoro Islands or Comores or Mayotte or Congo or Zaire or Costa Rica or Cote d'Ivoire or Ivory Coast or Cuba or Djibouti or French Somaliland or Dominica or Dominican Republic or East Timor or East Timur or Timor Leste or Ecuador or Egypt or United Arab Republic or El Salvador or Eritrea or Ethiopia or Fiji or Gabon or Gabonese Republic or Gambia or Gaza or Georgia Republic or Georgian Republic or Ghana or Gold Coast or Grenada or Guatemala or Guinea or Guinea-Bisau or Guam or Guiana or Guyana or Haiti or Honduras or India or Maldives or Indonesia or Iran or Iraq or Jamaica or Jordan or Kazakhstan or Kazakh or Kenya or Kiribati or Korea or Kosovo or Kyrgyzstan or Kirghizia or Kyrgyz Republic or Kirghiz or Kirgizstan or Lao PDR or Laos or Lebanon or Lesotho or Basutoland or Liberia or Libya or Macedonia or Madagascar or Malagasy Republic or Malaysia or Malaya or Malay or Sabah or Sarawak or Malawi or Nyasaland or Mali or Marshall Islands or Mauritania or Mauritius or Agalega Islands or Mexico or Micronesia or Middle East or Moldova or Moldovia or Moldovian or Mongolia or Montenegro or Morocco or Ifni or Mozambique or Myanmar or Myanma or Burma or Namibia or Nepal or Netherlands Antilles or Nicaragua or Niger or Nigeria or Pakistan or Palau or Palestine or Panama or Papua New Guinea or Paraguay or Peru or Philippines or Philipines or Phillipines or Phillippines or Romania or Rumania or Roumania or Rwanda or Ruanda or Saint Lucia or St Lucia or Saint Vincent or St Vincent or Grenadines or Samoa or Samoan Islands or Navigator Island or Navigator Islands or Sao Tome or Senegal or Serbia or Sierra Leone or Sri Lanka or Ceylon or Solomon Islands or Somalia or Sudan or Suriname or Surinam or Swaziland or Syria or Principe or South Sudan or Tajikistan or Tadzhikistan or Tadjikistan or Tadzhik or Tanzania or Thailand or Timor-Leste or Togo or Togolese Republic or Tonga or Tunisia or Turkey or Turkmenistan or Turkmen or Tuvalu or Uganda or Ukraine or Uzbekistan or Uzbek or Vanuatu or New Hebrides or Vietnam or Viet Nam or West Bank or Yemen or Zambia or Zimbabwe or Rhodesia)

For peer review only

14	(((developing or less* developed or under developed or underdeveloped or middle income or low* income or underserved or under served or deprived or poor*) N1 (countr* or nation? or state? or population? or world))) OR (((developing or less* developed or under developed or underdeveloped or middle income or low* income) N1 (economy or economies))) OR ((low* N1 (gdp or gnp or gross domestic or gross national))) OR (low N3 middle N3 countr*) OR (lmic or lmics or third world or lami countr*) OR transitional countr*
15	S12 OR S13 OR S14
16	S4 AND S11 AND S15

Cochrane Central Register of Controlled Trials

ID	Search
#1	MeSH descriptor: [Anemia, Iron-Deficiency] explode all trees
#2	MeSH descriptor: [Anemia] this term only
#3	MeSH descriptor: [Anemia, Hypochromic] this term only
#4	anaemia or anemia:ti (Word variations have been searched)
#5	((iron deficien* or hypochromic or microcytic) near (anaemia or anemia)):ti,ab,kw (Word variations have been searched)
#6	((pregnan* or gestation* or maternal) and (anaemia or anemia)):ti,ab,kw (Word variations have been searched)
#7	#1 or #2 or #3 or #4 or #5 or #6
#8	MeSH descriptor: [Labor, Obstetric] explode all trees
#9	MeSH descriptor: [Parturition] explode all trees
#10	MeSH descriptor: [Delivery, Obstetric] explode all trees
#11	labor or labour or birth? or childbirth? or delivery or obstetric*:ti (Word variations have been searched)
#12	((induction? or induced or inducing) near (labor or labour or birth? or childbirth? or delivery)):ti,ab,kw (Word variations have been searched)
#13	(augment* near (labor or labour or birth? or childbirth? or delivery)):ti,ab,kw (Word variations have been searched)
#14	(manage* near (labor or labour or birth? or childbirth? or delivery)):ti,ab,kw (Word variations have been searched)
#15	(initiat* near (labor or labour or birth? or childbirth? or delivery)):ti,ab,kw (Word variations have been searched)
#16	MeSH descriptor: [Oxytocin] explode all trees
#17	MeSH descriptor: [Prostaglandins E, Synthetic] explode all trees
#18	(uterotonic? or oxytocin or prostaglandin? or misoprostol):ti,ab,kw (Word variations have been searched)
#19	#8 or #9 or #10 or #11 or #12 or #13 or #14 or #15 or #16 or #17 or #18
#20	#7 and #19

For peer review only - http://bmjopen.bmj.com/site/about/guidelines.xhtml

Global Health Library

(anaemia OR anemia) AND (labour OR labor OR birth OR childbirth OR delivery OR obstetrics OR obstetric OR uterotonic OR uterotonics OR oxytocin OR prostaglandin OR prostaglandins OR misoprostol)
(iron deficiency anaemia OR iron deficiency anemia) AND (labour OR labor OR birth OR childbirth OR delivery OR obstetrics OR obstetric OR uterotonic OR uterotonics OR oxytocin OR prostaglandin OR prostaglandins OR misoprostol)
(hypochromic anaemia OR hypochromic anemia) AND (labour OR labor OR birth OR childbirth OR delivery OR obstetrics OR obstetric OR uterotonic OR uterotonics OR oxytocin OR prostaglandin OR prostaglandins OR misoprostol)
(microcytic anaemia OR microcytic anemia) AND (labour OR labor OR birth OR childbirth OR delivery OR obstetrics OR obstetric OR uterotonic OR uterotonics OR oxytocin OR prostaglandin OR prostaglandins OR misoprostol)

Dissertations & Theses (Global full text plus UK and Ireland abstracts)

Set	Search
S8	S1 AND S6
S6	ti(pregnancy OR pregnant)
S5	S1 AND S4
S4	S2 OR S3
S3	ti(uterotonic? or oxytocin or prostaglandin? or misoprostol)
S2	ti(labor or labour or birth? or childbirth? or delivery or obstetric*)
S1	ti(anaemia OR anemia)

Trials registers: Clinical trials.gov (https://clinicaltrials.gov/) and WHO ICTRP (http://apps.who.int/trialsearch/)

**Clinicaltrials.gov**
Condition=anaemia OR anemia AND Intervention=labour OR labor OR birth OR childbirth OR delivery OR obstetric OR obstetrics OR oxytocin OR prostaglandin OR prostaglandins OR misoprostol
Condition=anaemia OR anemia AND Title=labour OR labor OR birth OR childbirth OR delivery OR obstetric OR obstetrics OR oxytocin OR prostaglandin OR prostaglandins OR misoprostol
Condition=anaemia OR anemia AND Title=labour OR labor OR birth OR childbirth OR delivery OR obstetric OR obstetrics OR oxytocin OR prostaglandin OR prostaglandins OR misoprostol

**WHO Search:**
Condition=anaemia OR anemia AND Intervention=labour OR labor OR birth OR childbirth OR delivery OR obstetric OR obstetrics OR oxytocin OR prostaglandin OR prostaglandins OR misoprostol
Title=(anaemia OR anemia) AND Intervention=(labour OR labor OR birth OR childbirth OR delivery OR obstetric OR obstetrics OR oxytocin OR prostaglandin OR prostaglandins OR misoprostol)
Condition=anaemia OR anemia AND Title=labour OR labor OR birth OR childbirth OR delivery OR obstetric OR obstetrics OR oxytocin OR prostaglandin OR prostaglandins OR misoprostol

For peer review only - http://bmjopen.bmj.com/site/about/guidelines.xhtml

PRISMA 2009 Checklist

Section/topic	#	Checklist item	Reported on page #
**TITLE**			
Title	1	Identify the report as a systematic review, meta-analysis, or both.	1
**ABSTRACT**			
Structured summary	2	Provide a structured summary including, as applicable: background; objectives; data sources; study eligibility criteria, participants, and interventions; study appraisal and synthesis methods; results; limitations; conclusions and implications of key findings; systematic review registration number.	2
**INTRODUCTION**			
Rationale	3	Describe the rationale for the review in the context of what is already known.	4
Objectives	4	Provide an explicit statement of questions being addressed with reference to participants, interventions, comparisons, outcomes, and study design (PICOS).	4
**METHODS**			
Protocol and registration	5	Indicate if a review protocol exists, if and where it can be accessed (e.g., Web address), and, if available, provide registration information including registration number.	5
Eligibility criteria	6	Specify study characteristics (e.g., PICOS, length of follow-up) and report characteristics (e.g., years considered, language, publication status) used as criteria for eligibility, giving rationale.	5
Information sources	7	Describe all information sources (e.g., databases with dates of coverage, contact with study authors to identify additional studies) in the search and date last searched.	5
Search	8	Present full electronic search strategy for at least one database, including any limits used, such that it could be repeated.	6 and S1
Study selection	9	State the process for selecting studies (i.e., screening, eligibility, included in systematic review, and, if applicable, included in the meta-analysis).	5, 6, 7
Data collection process	10	Describe method of data extraction from reports (e.g., piloted forms, independently, in duplicate) and any processes for obtaining and confirming data from investigators.	7, 8
Data items	11	List and define all variables for which data were sought (e.g., PICOS, funding sources) and any assumptions and simplifications made.	7, 8
Risk of bias in individual studies	12	Describe methods used for assessing risk of bias of individual studies (including specification of whether this was done at the study or outcome level), and how this information is to be used in any data synthesis.	8
Summary measures	13	State the principal summary measures (e.g., risk ratio, difference in means).	8
Synthesis of results	14	Describe the methods of handling data and combining results of studies, if done, including measures of consistency (e.g., I^2) for each meta-analysis.	8

For peer review only - http://bmjopen.bmj.com/site/about/guidelines.xhtml

PRISMA 2009 Checklist

Section/topic	#	Checklist item	Reported on page #
Risk of bias across studies	15	Specify any assessment of risk of bias that may affect the cumulative evidence (e.g., publication bias, selective reporting within studies).	8
Additional analyses	16	Describe methods of additional analyses (e.g., sensitivity or subgroup analyses, meta-regression), if done, indicating which were pre-specified.	8
**RESULTS**			
Study selection	17	Give numbers of studies screened, assessed for eligibility, and included in the review, with reasons for exclusions at each stage, ideally with a flow diagram.	8, 9
Study characteristics	18	For each study, present characteristics for which data were extracted (e.g., study size, PICOS, follow-up period) and provide the citations.	9
Risk of bias within studies	19	Present data on risk of bias of each study and, if available, any outcome level assessment (see item 12).	9
Results of individual studies	20	For all outcomes considered (benefits or harms), present, for each study: (a) simple summary data for each intervention group (b) effect estimates and confidence intervals, ideally with a forest plot.	9 and Table-1 (10)
Synthesis of results	21	Present results of each meta-analysis done, including confidence intervals and measures of consistency.	9
Risk of bias across studies	22	Present results of any assessment of risk of bias across studies (see Item 15).	NA
Additional analysis	23	Give results of additional analyses, if done (e.g., sensitivity or subgroup analyses, meta-regression [see Item 16]).	NA
**DISCUSSION**			
Summary of evidence	24	Summarize the main findings including the strength of evidence for each main outcome; consider their relevance to key groups (e.g., healthcare providers, users, and policy makers).	11
Limitations	25	Discuss limitations at study and outcome level (e.g., risk of bias), and at review-level (e.g., incomplete retrieval of identified research, reporting bias).	11
Conclusions	26	Provide a general interpretation of the results in the context of other evidence, and implications for future research.	12, 13
**FUNDING**			
Funding	27	Describe sources of funding for the systematic review and other support (e.g., supply of data); role of funders for the systematic review.	16

From: Moher D, Liberati A, Tetzlaff J, Altman DG, The PRISMA Group (2009). Preferred Reporting Items for Systematic Reviews and Meta-Analyses: The PRISMA Statement. PLoS Med 6(7): e1000097. doi:10.1371/journal.pmed1000097

For more information, visit: www.prisma-statement.org.

For peer review only - http://bmjopen.bmj.com/site/about/guidelines.xhtml

BMJ Open

A systematic review to investigate the safety of induction and augmentation of labour among pregnant women with iron deficiency anaemia.

Journal:	*BMJ Open*
Manuscript ID	bmjopen-2018-021793.R1
Article Type:	Research
Date Submitted by the Author:	05-Jul-2018
Complete List of Authors:	Bunch, Kathryn; University of Oxford, National Perinatal Epidemiology Unit, Nuffield Department of Population Health<br>Roberts, Nia; University of Oxford, UK, Bodleian Health Care Libraries,<br>Knight, Marian; National Perinatal Epidemiology Unit<br>Nair, Manisha; University of Oxford, NPEU, Nuffield Department of Population Health
**Primary Subject Heading**:	Obstetrics and gynaecology
Secondary Subject Heading:	Public health, Global health
Keywords:	Pregnant women, Anaemia < HAEMATOLOGY, induction of labour, augmentation of labour, post-partum haemorrhage

SCHOLARONE™
Manuscripts

TITLE PAGE

A systematic review to investigate the safety of induction and augmentation of labour among pregnant women with iron deficiency anaemia

AUTHORS

1. Kathryn Bunch
   National Perinatal Epidemiology Unit, Nuffield Department of Population Health, University of Oxford, Richard Doll Building, Old Road Campus, Headington, Oxford, OX3 7LF, UK. Email: kathryn.bunch@npeu.ox.ac.uk
2. Nia Roberts
   Bodleian Libraries, University of Oxford, Knowledge Centre, Old Road Research Building, off Roosevelt Drive, Headington, Oxford, OX3 7DQ, UK.
   Email: nia.roberts@bodleian.ox.ac.uk
3. Marian Knight
   National Perinatal Epidemiology Unit, Nuffield Department of Population Health, University of Oxford, Richard Doll Building, Old Road Campus, Headington, Oxford, OX3 7LF, UK. Email: marian.knight@npeu.ox.ac.uk
4. Manisha Nair
   National Perinatal Epidemiology Unit, Nuffield Department of Population Health, University of Oxford, Richard Doll Building, Old Road Campus, Headington, Oxford, OX3 7LF, UK. Email: manisha.nair@npeu.ox.ac.uk

Corresponding author

Manisha Nair
National Perinatal Epidemiology Unit, Nuffield Department of Population Health,
University of Oxford, Richard Doll Building, Old Road Campus, Headington, Oxford, OX3 7LF, UK.
Email: manisha.nair@npeu.ox.ac.uk
Phone: +44 (0)1865617820

For peer review only

For peer review only - http://bmjopen.bmj.com/site/about/guidelines.xhtml

ABSTRACT

Objective: To conduct a systematic review to investigate the safety of induction and/ or augmentation of labour compared with spontaneous-onset normal labour among pregnant women with iron-deficiency anaemia.

Design: Systematic review.

Setting: Studies from all countries, worldwide.

Population: Pregnant women with iron-deficiency anaemia at labour and delivery.

Intervention: Any intervention related to induction and/ or augmentation of labour.

Outcome measures: Primary: Postpartum haemorrhage (PPH), heart failure and maternal death. Secondary: Emergency caesarean section, hysterectomy, admission to intensive care unit.

Method: We searched 10 databases, including Medline and Embase, from database inception to 07 November 2017. We included all study designs except cross-sectional studies without a comparator group, case reports, case series, ecological studies, and expert opinion. The searches were conducted by a healthcare librarian and two authors independently screened and reviewed the studies. We used the GRADE approach to ascertain risk of bias and conducted a narrative synthesis.

Results: We identified 2733 journal articles, 223 conference papers, 45 dissertations, and 218 registered trials. Ten articles were included for full-text review and only one was found to fulfil the eligibility criteria. This was a retrospective cohort study from India which showed that pregnant women with moderate and severe anaemia could have an increased risk of PPH if they underwent induction and/or augmentation of labour, but the evidence was weak (graded as 'High risk of bias').

Conclusion: The best approach is to prevent anaemia, but a large number of women in low-to-middle-income countries present with severe anaemia during labour. In such women, appropriate peripartum management could prevent complications and death. Our review showed that at present we do not know if induction and augmentation of labour is safe in pregnant women with iron deficiency anaemia and further research is required.

ARTICLE SUMMARY

Strengths and limitations of this study

- This is the first systematic review conducted to examine the evidence of the safety of induction and augmentation of labour in pregnant women with iron-deficiency anaemia.
- A rigorous search method was used to identify published and unpublished studies in any language from all countries, globally, using 10 databases from their inception till date.
- Despite a rigorous search over a period of two years, only one study was found.

For peer review only - http://bmjopen.bmj.com/site/about/guidelines.xhtml

Key words: Pregnant women; anaemia; iron-deficiency; induction of labour; augmentation of labour; post-partum haemorrhage.

Word count: 2262

For peer review only

For peer review only - http://bmjopen.bmj.com/site/about/guidelines.xhtml

INTRODUCTION

Iron-deficiency anaemia during pregnancy is defined as a haemoglobin concentration less than 11g/dl (110g/l) and is a major cause of maternal morbidity and mortality in low and middle income countries (LMICs)[1]. An estimated 42% of pregnant women have anaemia, globally[1], but the prevalence is reported to be as high as 70-80% in countries such as India[2]. Pregnant women with iron deficiency anaemia are at a higher risk of postpartum haemorrhage (PPH)[3] and heart failure[4] during pregnancy and childbirth. Clearly the ideal approach would be to prevent anaemia or treat it during the antenatal period, but a large number of pregnant women in LMICs present with severe anaemia either at the point of delivery or in the late third trimester of pregnancy[5 6] when there is little scope for prevention or iron treatment.

A systematic review showed that active management of the third stage of labour reduces the risk of PPH among pregnant women with haemoglobin concentrations <9g/dl, although the quality of evidence was low[7]. There is no clear evidence about the risks and benefits of induction and augmentation of labour during the first and second stages of labour among pregnant women with iron deficiency anaemia. A recent study showed that pregnant women with moderate-severe anaemia (haemoglobin <10g/dl) who underwent induction of labour were at an increased risk of PPH[8]. A few small studies have suggested that women with severe anaemia are more likely to have uterine atony due to impaired transport of oxygen rich haemoglobin to the uterus[3 9], but there is no strong evidence. This impaired transport of oxygen due to low haemoglobin could also result in early fatigue of the uterus following use of uterotonics [10-12] resulting in uterine atony and subsequent PPH. The aim of this systematic review was to investigate the safety of induction and/ or augmentation of labour compared with spontaneous-onset normal labour in pregnant women with anaemia.

For peer review only - http://bmjopen.bmj.com/site/about/guidelines.xhtml

METHOD

Study design: Systematic review of published and unpublished literature. The study was registered in PROSPERO (International prospective register of systematic reviews): CRD42015032421.

Eligibility criteria

Types of studies: We included all comparative study designs. Cross-sectional studies without a comparator group, case reports, case series, ecological studies, and expert opinion pieces were excluded.

Types of participant: Our search was restricted to pregnant women with iron deficiency anaemia >20 weeks gestation at labour and delivery. We excluded pregnant women with anaemia due to haemoglobinopathies.

Setting: We included studies from all countries.

We searched for published and unpublished literature in any language from the inception of the databases until 07 November 2017.

Information sources

The following electronic databases were searched for published literature: Medline, Embase, CINAHL (Cumulative Index to Nursing and Allied Health Literature), Global Health (OvidSP), Global Health Library, and Cochrane Central Register of Controlled Trials. Unpublished or grey literature searches were restricted to dissertations, reports and conference proceedings using two recognized databases: 'Index to theses' and 'Proquest Dissertations & Theses'. In addition, we looked for ongoing unpublished trials by searching trial registers such as www.ClinicalTrials.gov and the World Health Organisation (WHO) International Clinical Trials Registry Platform (www.who.int/trialsearch/). We also planned to hand search the reference lists of included studies.

For peer review only - http://bmjopen.bmj.com/site/about/guidelines.xhtml

Search strategy

NR carried out the electronic database searches. An EndNote bibliographic database was used to manage references, identify duplicates and share references among the reviewers. The screening was carried out independently by two reviewers (KB and MN) in two stages – (i) screening of titles and abstracts based on the pre-specified inclusion and exclusion criteria; (ii) screening of full-texts of the papers included during stage-1. Any disagreements on inclusion of studies were discussed and, where possible, resolved by consensus after referring to the review protocol or a third reviewer (MK). A record of decisions made for each article was maintained. The search strategy was piloted by applying the inclusion and exclusion criteria to a sample of papers to check its reliability in correctly classifying the studies. The process helped to refine the selection criteria and the search strategies (provided in the supplementary document: S1 Text). Since the focus of this review was on the safety of induction and/ or augmentation of labour compared with spontaneous-onset normal labour in pregnant women with anaemia, the key components of our search were: (i) population – pregnant women with anaemia and (ii) intervention - induction and/ or augmentation of labour. We did not restrict the breadth of the search by specifying any outcome. We hand-searched key papers and additional reference linking was done by the authors. The PRISMA flow chart was used to summarise the selection process.

Types of interventions

We searched for studies that included any intervention related to induction of labour, augmentation of labour, and combined induction and augmentation of labour; with and without active management of the third stage in women with iron deficiency anaemia. Key definitions of interventions and outcomes are provided in Box-1.

Comparisons

Pregnant women with anaemia who had spontaneous-onset normal labour..

For peer review only

For peer review only - http://bmjopen.bmj.com/site/about/guidelines.xhtml

Types of outcome measures

Adverse maternal outcomes during labour, delivery and within 24 hours of childbirth.

Primary outcomes:

- Primary postpartum haemorrhage (defined in Box-1) or requiring therapeutic uterotonics or transfusion of blood or blood products during labour or delivery up to 24 hours after delivery.
- Heart failure during labour or delivery or up to 24 hours after delivery.
- Maternal death.

Secondary outcomes:

- Emergency caesarean section.
- Hysterectomy.
- Admission to intensive care unit.
- Any other adverse maternal outcome

Box-1: Key definitions

- Iron deficiency anaemia among pregnant women is defined as a haemoglobin concentration of less than 11g/dl and can be categorised as mild anaemia (haemoglobin 10-10.9g/dl), moderate anaemia (haemoglobin 7-9·9 g/dl) and severe anaemia (haemoglobin <7 g/dl)[13].
- Induction of labour is defined as 'the initiation of labour by artificial means prior to its spontaneous onset at a viable gestational age, with the aim of achieving vaginal delivery in a pregnant woman with intact membranes'[14].
- Augmentation of labour is the process of stimulating the uterus to increase the frequency, duration and intensity of contractions after the onset of labour through intravenous oxytocin infusion and/ or artificial rupture of membranes[15].
- Active management of the third stage of labour is 'a prophylactic intervention composed of a package of three components or steps: 1) administration of a uterotonic, preferably oxytocin, immediately after birth of the baby; 2) controlled cord traction to deliver the placenta; and 3) massage of the uterine fundus after the placenta is delivered.' New recommendations clarify that administration of a uterotonic is the key component, the other two being optional[16].
- A maternal death is the death of a woman while pregnant or within 42 days of the end of pregnancy, irrespective of the duration and the site of the pregnancy, from any cause related to or aggravated by the pregnancy or its management, but not from accidental or incidental causes[17].
- Primary postpartum haemorrhage is defined as a pregnant women with a blood

For peer review only - http://bmjopen.bmj.com/site/about/guidelines.xhtml

loss of 500 ml or more from the genital tract within 24 hours of giving birth[16].

Data collection and analysis

The data extraction forms and coding sheets were developed after reviewing a subset of sample papers followed by testing the tools in the remaining sub-set to ensure that the tools captured all relevant information[18]. Data extraction from included studies was performed independently by two reviewers (MN and KB) and we planned to measure the level of inter-reviewer agreement using a Kappa statistic (a measure of chance-corrected agreement)[19]. As with selection of studies, we planned to resolve any disagreement through consensus or through arbitration by a third reviewer (MK). We planned to note the corrections/ amendments in the data extraction forms.

Risk of bias (quality) assessment

We used the GRADE approach to ascertain risk of bias and categorise studies into 'low risk', 'high risk' and 'unclear risk', and judgement was substantiated by a text description. We planned to assess publication bias through visual inspection and by conducting tests for asymmetry of funnel plots if at least five studies were included in the meta-analysis.

Strategy for data synthesis

We proposed to use both quantitative and narrative synthesis. Depending on the presence or absence of statistical, clinical and methodological heterogeneity of the included studies, a random-effects model or a fixed-effects model would be used to generate pooled estimates of the outcome. If clinical and methodological heterogeneity are observed, sensitivity analyses would be conducted to understand their effects on the pooled estimates. We also planned to conduct subgroup analysis to ascertain if the maternal outcomes of induction of labour and/ or augmentation of labour vary according to the severity of iron deficiency anaemia among pregnant women.

Patient and Public Involvement: Not applicable since this was a systematic review.

For peer review only - http://bmjopen.bmj.com/site/about/guidelines.xhtml

RESULTS

The search of published and unpublished studies identified 1831 journal articles from LMICs and 902 articles from high income countries (HICs), 223 conference papers, 45 dissertations, and 218 registered trials after removing duplicates. Countries were categorised into income groups (HIC and LMIC) based on the World Bank's classification for 2017-18 (https://datahelpdesk.worldbank.org/knowledgebase/articles/906519-world-bank-country-and-lending-groups). After screening the title and abstracts of all articles, conference proceedings and dissertations against inclusion and exclusion criteria, eight journal articles were included for full-text review from LMICs and two from HICs.

Six out of the eight studies from LMICs did not meet the inclusion criteria. The studies either did not include pregnant women with anaemia or did not include any information about the interventions of interest. One study from a hospital in India examined separately the proportion of women who underwent induction of labour and the proportion who had a postpartum haemorrhage among a total of 447 pregnant women with varying degrees of anaemia [9]. The study showed that a higher proportion of women who had severe anaemia underwent induction of labour compared with women who did not have anaemia[9]. The proportion of women who had a postpartum haemorrhage was also higher in the severe anaemia group[9]. We contacted the authors for data on postpartum haemorrhage sub-divided by induction of labour versus spontaneous vaginal delivery for each group of women with varying degrees of anaemia, but were unable to get any response despite three attempts.

The only study found to fulfil the inclusion and exclusion criteria was our previous study that led to the hypothesis for this systematic review. This was a retrospective cohort study among 1,007 pregnant women from five teaching hospitals in India. It showed that compared with women who have a normal haemoglobin concentration, women who have moderate-severe anaemia have a significantly higher odds of postpartum haemorrhage and notably, the odds increased 17-fold among women with moderate and severe anaemia who underwent induction of labour[8]. However, the evidence from the study was not strong due to a 'high risk'

For peer review only - http://bmjopen.bmj.com/site/about/guidelines.xhtml

of bias assessed using the GRADE classification. The two studies from HICs were published in Polish, but none were found to meet the inclusion criteria. The reasons for exclusion are shown in Table-1 and a PRISMA flow-chart showing the selection process is presented in Figure-1.

Table-1: Reasons for exclusion of studies after full-text review

Table-1: Reasons for exclusion of studies after full-text review

Study reference	Reasons for exclusion
**Studies from LMICs**	
Tee ES, Kandiah M, Ali J, et al. (1984)[20]	This was a descriptive study of the prevalence and outcomes of nutritional anaemia among 309 pregnant women in a maternity hospital in Malaysia. Thus it did not fit the inclusion criteria for study design, interventions and outcomes.
Tsu VD. (1993)[21]	Pregnant women who were induced with oxytocin before delivery were excluded, hence no intervention or comparator.
Phillip H, Fletcher H, Reid M. (2004)[22]	Pregnant women with anaemia were excluded from the study.
Kavle JA, Stoltzfus RJ, Witter F, et al.(2008)[3]	Did not include any information on the intervention of interest – induction and/ or augmentation of labour compared with spontaneous vaginal delivery. The study showed that ergometrine and/ or oxytocins in the third stage of labour were significantly associated with increased odds of postpartum haemorrhage.
Wang X, Tan H, Zhou S, et al (2014)[23]	This was a case-control study that examined the risk factors for PPH. Anaemia was found to be an independent risk factors for PPH, but management of labour and delivery was not examined.
Doncev V (1974)[24]	This study compared the proportions of maternal and fetal complications between 138 pregnant women with anaemia and 300 pregnant women without anaemia. Higher proportions of women with anaemia had preterm delivery, prolonged labour, uterine inertia, and birth asphyxia compared with women with no anaemia. The study did not fit the inclusion criterion for interventions.
**Studies from HICs**	
Salwa M (1975)[25]	This study examined complications during labour and delivery in 309 pregnant women and reported a higher prevalence of PPH in women with anaemia (11-75%) compared with women with no anaemia (8.2%). The study did not fit the inclusion criterion for interventions.
Daraz B (1978)[26]	This cross-sectional study of 2128 pregnant women during labour showed that the average duration of

For peer review only - http://bmjopen.bmj.com/site/about/guidelines.xhtml

	the first and second stages of labour increased with a decrease in the concentration of haemoglobin, but the association was statistically significant for multiparous women only. The study did not fit the inclusion criteria for study design, interventions and outcomes.

LMICs–Low and middle income countries; HICs–High income countries; PPH–Postpartum haemorrhage

DISCUSSION

This systematic search of published and unpublished studies in 10 databases using key words and search criteria found only one study which showed that pregnant women with moderate and severe anaemia had an increased risk of postpartum haemorrhage (PPH) if they underwent induction and/or augmentation of labour, but the evidence was not strong. We did not find any other study that examined the clinical management of anaemic pregnant women during labour and delivery, specifically the association of induction and/or augmentation of labour with adverse maternal outcomes.

This is the first systematic review conducted to examine the evidence of the safety of induction and augmentation of labour compared with spontaneous-onset normal labour in pregnant women with iron-deficiency anaemia. A broad rigorous search of published and unpublished studies in any language from all countries, globally, using 10 databases from their inception till date, identified only one study that fulfilled the eligibility criteria. The results of this review are therefore inconclusive. Despite several attempts we could not obtain any further information for another study from India.

While some studies investigating the risk factors for PPH found that induction of labour[27 28], prolonged second stage of labour[28] and anaemia[28] were independently associated with PPH, others suggest that anaemia is associated with prolonged labour which in turn increases the risk of PPH[3 9]. A recent study from Egypt showed that compared with women who have normal haemoglobin or mild anaemia, pregnant women with moderate to severe anaemia have a higher level of nitric oxide which signals smooth muscle relaxation leading to uterine

For peer review only - http://bmjopen.bmj.com/site/about/guidelines.xhtml

atony in the presence of tissue hypoxia[29], which can be further aggravated by uterotonics used for induction and augmentation of labour[10-12], resulting in PPH.

A prospective cohort study of about 11,000 pregnant women with moderate and severe anaemia is currently being undertaken in India through the Maternal and perinatal Health Research Collaboration, India (MaatHRI) to examine the safety of induction and augmentation of labour in this group[30]. This study will also investigate the effect of different methods of induction (mechanical using catheters and hygroscopic dilators or pharmacological using intravenous oxytocin and/ or intravaginal prostaglandins or misoprostol) and augmentation of labour (intravenous oxytocin infusion and/ or artificial rupture of membranes) on the risk of PPH and if active management of the third stage of labour modifies the risk. If an increased risk is demonstrated, there could be a potential role of the anti-fibrinolytic drug, tranexamic acid (TXA)[31], in preventing PPH in pregnant women with anaemia who undergo induction and/ or augmentation of labour, but we need further studies to find an effective dose of TXA that could prevent haemorrhage in pregnant women with moderate and severe anaemia.

Conclusion

This review gives no clear evidence regarding management of labour in pregnant women with iron deficiency anaemia. Studies suggest that pregnant women with anaemia are more likely to have sluggish uterine contractions[9 29] leading to prolonged second stage of labour and uterine atony[9 23] and consequently PPH. Despite several interventions such as routine iron supplementation during the antenatal period, there has been the best a very small decline in the prevalence of anaemia among pregnant women globally since 1995 [32]. A large number of pregnant women present with anaemia late in pregnancy or during labour. In such women appropriate peripartum care could prevent severe complications and death. The WHO provides guidance on induction and augmentation of labour and delivery[15 33], but it does not include any information about the safety of induction and/or augmentation of labour

For peer review only - http://bmjopen.bmj.com/site/about/guidelines.xhtml

in pregnant women with anaemia. At present, the decision to induce and/or augment labour in these women is not evidence-based and depends on the clinician's judgement. There is also an additional risk of unnecessary intervention or 'too much too soon' in several LMICs[34]. If induction and/ or augmentation is required, then knowing about the increased risk is important to prepare healthcare providers to proactively manage any imminent complication by augmented active management of the third stage, arranging blood/ blood products for transfusion and drugs such as tranexamic acid, which are usually not readily available in a low-resource setting. Thus, there is an urgent need to conduct further research to investigate the safety of induction and augmentation of labour in pregnant women with anaemia.

REFERENCES

1. de Benoist B, McLean E, Egli I, et al., editors. *Worldwide prevalence of anaemia 1993–2005 : WHO global database on anaemia*. Geneva: World Health Organisation, 2008.
2. Toteja GS, Singh P, Dhillon BS, et al. Prevalence of anemia among pregnant women and adolescent girls in 16 districts of India. Food and nutrition bulletin 2006;**27**(4):311-5.
3. Kavle JA, Stoltzfus RJ, Witter F, et al. Association between Anaemia during Pregnancy and Blood Loss at and after Delivery among Women with Vaginal Births in Pemba Island, Zanzibar, Tanzania. Journal of Health, Population, and Nutrition 2008;**26**(2):232-40.
4. Grewal A. Anaemia and pregnancy: Anaesthetic implications. Indian Journal of Anaesthesia 2010;**54**(5):380-86.
5. Chhabra S, Sirohi R. Trends in maternal mortality due to haemorrhage: two decades of Indian rural observations. Journal of Obstetrics & Gynecology 2004;**24**(1):40-43.
6. Rohilla M, Raveendran A, Dhaliwal L, et al. Severe anaemia in pregnancy: a tertiary hospital experience from northern India. Journal of Obstetrics & Gynaecology 2010;**30**(7):694-96.
7. Begley Cecily M, Gyte Gillian ML, Devane D, et al. Active versus expectant management for women in the third stage of labour. Cochrane Database of Systematic Reviews 2015; (3). http://onlinelibrary.wiley.com/doi/10.1002/14651858.CD007412.pub4/abstract

http://onlinelibrary.wiley.com/store/10.1002/14651858.CD007412.pub4/asset/CD007412.pdf?v=1&t=ihrpfx40&s=7958f2208cd4269e8081aed33c7a9bf798ef1221.
8. Nair M, Choudhury MK, Choudhury SS, et al. The association between maternal anaemia and pregnancy outcomes: a cohort study in Assam, India. BMJ Global Health 2016;**1**:e000026.
9. Malhotra M, Sharma JB, Batra S, et al. Maternal and perinatal outcome in varying degrees of anemia. International Journal of Gynecology & Obstetrics 2002;**79**(2):93-100.
10. Li H, Gudmundsson S, Olofsson P. Uterine artery blood flow velocity waveforms during uterine contractions. Ultrasound in obstetrics & gynecology : the official journal of the International Society of Ultrasound in Obstetrics and Gynecology 2003;**22**(6):578-85.
11. Brotanek V, Hendricks CH, Yoshida T. Changes in uterine blood flow during uterine contractions. American journal of obstetrics and gynecology 1969;**103**(8):1108-16.
12. Stanton CK, Deepak NN, Mallapur AA, et al. Direct observation of uterotonic drug use at public health facility-based deliveries in four districts in India. International journal of gynaecology and obstetrics: the official organ of the International Federation of Gynaecology and Obstetrics 2014;**127**(1):25-30.

For peer review only - http://bmjopen.bmj.com/site/about/guidelines.xhtml

13. WHO, UNICEF, UNU. Iron deficiency anaemia: assessment, prevention and control, a guide for programme managers. 2001. http://www.who.int/nutrition/publications/micronutrients/anaemia_iron_deficiency/WHO_NHD_01.3/en/index.html (accessed 11 April 2014).
14. Eke AC, Okigbo C. Mechanical methods for induction of labour: RHL commentary (last revised: 1 August 2012) Geneva: World Health Organization: The WHO Reproductive Health Library; [cited 2015 02 December]. Available from: http://apps.who.int/rhl/pregnancy_childbirth/induction/cd001233_ekea_com/en/index.html.
15. World Health Organisation. *WHO recommendations for augmentation of labour*. Geneva: World Health Organisation, 2014.
16. World Health Organisation. *WHO recommendations for the prevention and treatment of postpartum haemorrhage*. Geneva, Switzerland World Health Organisation, 2012.
17. World Health Organisation. *The WHO application of ICD-10 to deaths during pregnancy, childbirth and puerperium: ICD MM*. Geneva World Health Organisation.
18. Brown SA, Upchurch SL, Acton GJ. A framework for developing a coding scheme for meta-analysis. Western journal of nursing research 2003;**25**(2):205-22.
19. Altman DG. Measuring agreement. In: Altman DG, ed. Practical statistics for medical research. London: Chapman and Hall, 1991.
20. Tee ES, Kandiah M, Ali J, et al. Nutritional anemia in pregnancy: a study at the maternity hospital, Kuala Lumpur. Malays J Reprod Health 1984;**2**(1):32-50.
21. Tsu VD. Postpartum haemorrhage in Zimbabwe: a risk factor analysis. Br J Obstet Gynaecol 1993;**100**(4):327-33.
22. Phillip H, Fletcher H, Reid M. The impact of induced labour on postpartum blood loss. J Obstet Gynaecol 2004;**24**(1):12-5.
23. Wang X, Tan H, Zhou S, et al. [Incidence and risk factors for postpartum hemorrhage in Liuyang, China]. Zhong Nan Da Xue Xue Bao Yi Xue Ban 2014;**Yi Xue Ban = Journal of Central South University. Medical Sciences. 39**(2):151-6.
24. Donchev V. [The effects of anemia during pregnancy on the course of labor]. Akush Ginekol (Mosk) 1974;**13**(6):421-30.
25. Salwa M. [Effect of anemia in pregnancy on labor complications]. Ginekol Pol 1975;**46**(5):513-7.
26. Daraz B. [Clinical study of labor management in women with anemia]. Ginekol Pol 1978;**49**(9):829-30.
27. Sheldon WR, Blum J, Vogel JP, et al. Postpartum haemorrhage management, risks, and maternal outcomes: findings from the World Health Organization Multicountry Survey on Maternal and Newborn Health. Bjog 2014;**121 Suppl 1**:5-13.
28. Wetta LA, Szychowski JM, Seals S, et al. Risk factors for uterine atony/postpartum hemorrhage requiring treatment after vaginal delivery. Am J Obstet Gynecol 2013;**209**(1):51.e1-6.
29. Soltan MH, Ibrahim EM, Tawfek M, et al. Raised nitric oxide levels may cause atonic postpartum hemorrhage in women with anemia during pregnancy. International journal of gynaecology and obstetrics: the official organ of the International Federation of Gynaecology and Obstetrics 2012;**116**(2):143-7.
30. National Perinatal Epidemiology Unit. MaatHRI: Maternal and perinatal Health Research collaboration, India Oxford: National Perinatal Epidemiology Unit, University of Oxford; 2017 [cited 2017 14 December]. Available from: https://www.npeu.ox.ac.uk/maathri.
31. Shakur H, Roberts I, Fawole B, et al. Effect of early tranexamic acid administration on mortality, hysterectomy, and other morbidities in women with post-partum haemorrhage (WOMAN): an international, randomised, double-blind, placebo-controlled trial. The Lancet 2017;**389**(10084):2105-16.
32. Stevens GA, Finucane MM, De-Regil LM, et al. Global, regional, and national trends in haemoglobin concentration and prevalence of total and severe anaemia in children and

For peer review only - http://bmjopen.bmj.com/site/about/guidelines.xhtml

pregnant and non-pregnant women for 1995-2011: a systematic analysis of population-representative data. The Lancet Global Health 2014;**1**(1):e16-e25.
33. WHO. *WHO recommendations for induction of labour*. Geneva: WHO, 2011.
34. Miller S, Abalos E, Chamillard M, et al. Beyond too little, too late and too much, too soon: a pathway towards evidence-based, respectful maternity care worldwide. The Lancet 2016;**388**(10056):2176-92.

Supplementary file S1: Search strategies

Acknowledgement: We would like to acknowledge the help from Anna Balchan, UKOSS/BAPS-CASS Programme Administrator at the NPEU, in reading and translating the papers published in Polish.

Disclosure of interests: The authors declare that they have no competing interests.

Contribution to authorship: KB reviewed the literature, extracted information from studies and edited the paper; NR conducted the searches and edited the paper; MK was the third reviewer and edited the paper; MN developed the concept for the systematic review, reviewed the literature, extracted information from studies and wrote the first draft of the paper.

Details of ethics approval: Not applicable since this is a systematic review.

Funding: The work was funded by a Medical Research Council (MRC) Career Development Award (https://www.mrc.ac.uk/) for Manisha Nair (Grant Ref: MR/P022030/1). The funder had no role in study design, data collection, data analysis, data interpretation, or writing of the report. The corresponding author had full access to all the data in the study and had final responsibility for the decision to submit for publication.

Data sharing statement: Data sharing is not applicable to this article as no datasets were generated or analysed during the review.

Figure legend

Figure-1: PRISMA flow-chart of the selection process for the systematic review

For peer review only - http://bmjopen.bmj.com/site/about/guidelines.xhtml

Figure-1: PRISMA flow-chart of the selection process for the systematic review

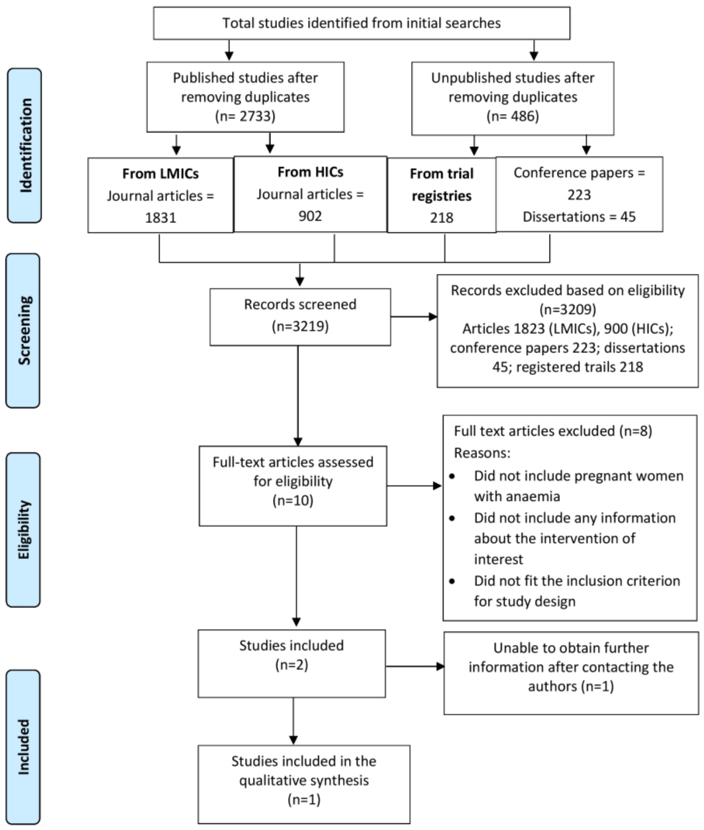

LMICs: Low-to-middle income countries; HICs: High income countries

Figure-1

209x297mm (300 x 300 DPI)

S1 Text

Search strategies

Medline

# ▲	Searches
1	anemia/ or anemia, hypochromic/ or anemia, iron-deficiency/
2	(anaemia or anemia).ti.
3	((iron deficien* or hypochromic or microcytic) adj5 (anaemia or anemia)).ti,ab.
4	((pregnan* or gestation* or maternal) and (anaemia or anemia)).ti,ab.
5	(Pregnancy/ or Pregnant woman/) and (anaemia or anemia).mp.
6	1 or 2 or 3 or 4 or 5
7	exp Labor, Obstetric/
8	exp Parturition/
9	exp Delivery, Obstetric/
10	(labor or labour or birth? or childbirth? or delivery or obstetric*).ti.
11	((induction? or induced or inducing) adj5 (labor or labour or birth? or childbirth? or delivery)).ti,ab.
12	(augment* adj5 (labor or labour or birth? or childbirth? or delivery)).ti,ab.
13	(manage* adj5 (labor or labour or birth? or childbirth? or delivery)).ti,ab.
14	(initiat* adj5 (labor or labour or birth? or childbirth? or delivery)).ti,ab.
15	Oxytocin/
16	exp Prostaglandins E, Synthetic/
17	(uterotonic? or oxytocin or prostaglandin? or misoprostol).ti,ab.
18	7 or 8 or 9 or 10 or 11 or 12 or 13 or 14 or 15 or 16 or 17
19	Developing Countries/
20	(Africa or Caribbean or West Indies or South America or Latin America or Central America).hw,kf,ti,ab,cp.

For peer review only

21	(Afghanistan or Albania or Algeria or Angola or American Samoa or Armenia or Armenian or Azerbaijan or Bangladesh or Benin or Byelarus or Byelorussian or Belarus or Belorussian or Belorussia or Belize or Bhutan or Bolivia or Bosnia or Herzegovina or Hercegovina or Botswana or Brazil or Brasil or Bulgaria or Burkina Faso or Burkina Fasso or Upper Volta or Burundi or Urundi or Cambodia or Khmer Republic or Kampuchea or Cameroon or Cameroons or Cameron or Camerons or Cape Verde or Central African Republic or Chad or China or Colombia or Comoros or Comoro Islands or Comores or Mayotte or Congo or Zaire or Costa Rica or Cote d'Ivoire or Ivory Coast or Cuba or Djibouti or French Somaliland or Dominica or Dominican Republic or East Timor or East Timur or Timor Leste or Ecuador or Egypt or United Arab Republic or El Salvador or Eritrea or Ethiopia or Fiji or Gabon or Gabonese Republic or Gambia or Gaza or Georgia Republic or Georgian Republic or Ghana or Gold Coast or Grenada or Guatemala or Guinea or Guinea-Bisau or Guam or Guiana or Guyana or Haiti or Honduras or India or Maldives or Indonesia or Iran or Iraq or Jamaica or Jordan or Kazakhstan or Kazakh or Kenya or Kiribati or Korea or Kosovo or Kyrgyzstan or Kirghizia or Kyrgyz Republic or Kirghiz or Kirgizstan or Lao PDR or Laos or Lebanon or Lesotho or Basutoland or Liberia or Libya or Macedonia or Madagascar or Malagasy Republic or Malaysia or Malaya or Malay or Sabah or Sarawak or Malawi or Nyasaland or Mali or Marshall Islands or Mauritania or Mauritius or Agalega Islands or Mexico or Micronesia or Middle East or Moldova or Moldovia or Moldovian or Mongolia or Montenegro or Morocco or Ifni or Mozambique or Myanmar or Myanma or Burma or Namibia or Nepal or Netherlands Antilles or Nicaragua or Niger or Nigeria or Pakistan or Palau or Palestine or Panama or Papua New Guinea or Paraguay or Peru or Philippines or Philipines or Phillipines or Phillippines or Romania or Rumania or Roumania or Rwanda or Ruanda or Saint Lucia or St Lucia or Saint Vincent or St Vincent or Grenadines or Samoa or Samoan Islands or Navigator Island or Navigator Islands or Sao Tome or Senegal or Serbia or Sierra Leone or Sri Lanka or Ceylon or Solomon Islands or Somalia or Sudan or Suriname or Surinam or Swaziland or Syria or Principe or South Sudan or Tajikistan or Tadzhikistan or Tadjikistan or Tadzhik or Tanzania or Thailand or Timor-Leste or Togo or Togolese Republic or Tonga or Tunisia or Turkey or Turkmenistan or Turkmen or Tuvalu or Uganda or Ukraine or Uzbekistan or Uzbek or Vanuatu or New Hebrides or Vietnam or Viet Nam or West Bank or Yemen or Zambia or Zimbabwe or Rhodesia).hw,kf,ti,ab,cp.
22	((developing or less* developed or under developed or underdeveloped or middle income or low* income or underserved or under served or deprived or poor*) adj (countr* or nation? or state? or population? or world)).ti,ab.
23	((developing or less* developed or under developed or underdeveloped or middle income or low* income) adj (economy or economies)).ti,ab.
24	(low* adj (gdp or gnp or gross domestic or gross national)).ti,ab.
25	(low adj3 middle adj3 countr*).ti,ab.
26	(lmic or lmics or third world or lami countr*).ti,ab.
27	transitional countr*.ti,ab.
28	19 or 20 or 21 or 22 or 23 or 24 or 25 or 26 or 27
29	6 and 18 and 28

For peer review only - http://bmjopen.bmj.com/site/about/guidelines.xhtml

Embase

# ▲	Searches
1	*anemia/ or iron deficiency anemia/ or microcytic anemia/
2	(anaemia or anemia).ti.
3	((iron deficien* or hypochromic or microcytic) adj5 (anaemia or anemia)).ti,ab.
4	((pregnan* or gestation* or maternal) and (anaemia or anemia)).ti,ab.
5	(Pregnancy/ or Pregnant woman/) and (anaemia or anemia).mp.
6	1 or 2 or 3 or 4 or 5
7	exp labor/
8	childbirth/
9	exp delivery/
10	(labor or labour or birth? or childbirth? or delivery or obstetric*).ti.
11	((induction? or induced or inducing) adj5 (labor or labour or birth? or childbirth? or delivery)).ti,ab.
12	(augment* adj5 (labor or labour or birth? or childbirth? or delivery)).ti,ab.
13	(manage* adj5 (labor or labour or birth? or childbirth? or delivery)).ti,ab.
14	(initiat* adj5 (labor or labour or birth? or childbirth? or delivery)).ti,ab.
15	exp uterotonic agent/
16	(uterotonic? or oxytocin or prostaglandin? or misoprostol).ti,ab.
17	7 or 8 or 9 or 10 or 11 or 12 or 13 or 14 or 15 or 16
18	Developing Country.sh.
19	(Africa or Caribbean or West Indies or South America or Latin America or Central America).hw,ti,ab,cp.

For peer review only

For peer review only - http://bmjopen.bmj.com/site/about/guidelines.xhtml

20	(Afghanistan or Albania or Algeria or Angola or American Samoa or Armenia or Armenian or Azerbaijan or Bangladesh or Benin or Byelarus or Byelorussian or Belarus or Belorussian or Belorussia or Belize or Bhutan or Bolivia or Bosnia or Herzegovina or Hercegovina or Botswana or Brazil or Brasil or Bulgaria or Burkina Faso or Burkina Fasso or Upper Volta or Burundi or Urundi or Cambodia or Khmer Republic or Kampuchea or Cameroon or Cameroons or Cameron or Camerons or Cape Verde or Central African Republic or Chad or China or Colombia or Comoros or Comoro Islands or Comores or Mayotte or Congo or Zaire or Costa Rica or Cote d'Ivoire or Ivory Coast or Cuba or Djibouti or French Somaliland or Dominica or Dominican Republic or East Timor or East Timur or Timor Leste or Ecuador or Egypt or United Arab Republic or El Salvador or Eritrea or Ethiopia or Fiji or Gabon or Gabonese Republic or Gambia or Gaza or Georgia Republic or Georgian Republic or Ghana or Gold Coast or Grenada or Guatemala or Guinea or Guinea-Bisau or Guam or Guiana or Guyana or Haiti or Honduras or India or Maldives or Indonesia or Iran or Iraq or Jamaica or Jordan or Kazakhstan or Kazakh or Kenya or Kiribati or Korea or Kosovo or Kyrgyzstan or Kirghizia or Kyrgyz Republic or Kirghiz or Kirgizstan or Lao PDR or Laos or Lebanon or Lesotho or Basutoland or Liberia or Libya or Macedonia or Madagascar or Malagasy Republic or Malaysia or Malaya or Malay or Sabah or Sarawak or Malawi or Nyasaland or Mali or Marshall Islands or Mauritania or Mauritius or Agalega Islands or Mexico or Micronesia or Middle East or Moldova or Moldovia or Moldovian or Mongolia or Montenegro or Morocco or Ifni or Mozambique or Myanmar or Myanma or Burma or Namibia or Nepal or Netherlands Antilles or Nicaragua or Niger or Nigeria or Pakistan or Palau or Palestine or Panama or Papua New Guinea or Paraguay or Peru or Philippines or Philipines or Phillipines or Phillippines or Romania or Rumania or Roumania or Rwanda or Ruanda or Saint Lucia or St Lucia or Saint Vincent or St Vincent or Grenadines or Samoa or Samoan Islands or Navigator Island or Navigator Islands or Sao Tome or Senegal or Serbia or Sierra Leone or Sri Lanka or Ceylon or Solomon Islands or Somalia or Sudan or Suriname or Surinam or Swaziland or Syria or Principe or South Sudan or Tajikistan or Tadzhikistan or Tadjikistan or Tadzhik or Tanzania or Thailand or Timor-Leste or Togo or Togolese Republic or Tonga or Tunisia or Turkey or Turkmenistan or Turkmen or Tuvalu or Uganda or Ukraine or Uzbekistan or Uzbek or Vanuatu or New Hebrides or Vietnam or Viet Nam or West Bank or Yemen or Zambia or Zimbabwe or Rhodesia).hw,ti,ab,cp.
21	((developing or less* developed or under developed or underdeveloped or middle income or low* income or underserved or under served or deprived or poor*) adj (countr* or nation? or state? or population? or world)).ti,ab.
22	((developing or less* developed or under developed or underdeveloped or middle income or low* income) adj (economy or economies)).ti,ab.
23	(low* adj (gdp or gnp or gross domestic or gross national)).ti,ab.
24	(low adj3 middle adj3 countr*).ti,ab.
25	(lmic or lmics or third world or lami countr*).ti,ab.
26	transitional countr*.ti,ab.
27	18 or 19 or 20 or 21 or 22 or 23 or 24 or 25 or 26
28	6 and 17 and 27

For peer review only

For peer review only - http://bmjopen.bmj.com/site/about/guidelines.xhtml

Global Health

# ▲	Searches
1	anaemia/ or hypochromic anaemia/ or iron deficiency anaemia/ or microcytic anaemia/
2	(anaemia or anemia).ti.
3	((iron deficien* or hypochrmomic or microcytic) adj5 (anaemia or anemia)).ti,ab.
4	((pregnan* or gestation* or maternal) and (anaemia or anemia)).ti,ab.
5	1 or 2 or 3 or 4
6	birth/ or childbirth/ or parturition/
7	(labor or labour or birth? or childbirth? or delivery or obstetric*).ti.
8	((induction? or induced or inducing) adj5 (labor or labour or birth? or childbirth? or delivery)).ti,ab.
9	(augment* adj5 (labor or labour or birth? or childbirth? or delivery)).ti,ab.
10	(manage* adj5 (labor or labour or birth? or childbirth? or delivery)).ti,ab.
11	oxytocin/
12	exp synthetic prostaglandins/
13	(uterotonic? or oxytocin or prostaglandin? or misoprostol).ti,ab.
14	6 or 7 or 8 or 9 or 10 or 11 or 12 or 13
15	exp developing countries/ or exp africa/
16	(Africa or Caribbean or West Indies or South America or Latin America or Central America).hw,ti,ab,cp.
17	(Afghanistan or Albania or Algeria or Angola or American Samoa or Armenia or Armenian or Azerbaijan or Bangladesh or Benin or Byelarus or Byelorussian or Belarus or Belorussian or Belorussia or Belize or Bhutan or Bolivia or Bosnia or Herzegovina or Hercegovina or Botswana or Brazil or Brasil or Bulgaria or Burkina Faso or Burkina Fasso or Upper Volta or Burundi or Urundi or Cambodia or Khmer Republic or Kampuchea or Cameroon or Cameroons or Cameron or Camerons or Cape Verde or Central African Republic or Chad or China or Colombia or Comoros or Comoro Islands or Comores or Mayotte or Congo or Zaire or Costa Rica or Cote d'Ivoire or Ivory Coast or Cuba or Djibouti or French Somaliland or Dominica or Dominican Republic or East Timor or East Timur or Timor Leste or Ecuador or Egypt or United Arab Republic or El Salvador or Eritrea or Ethiopia or Fiji or Gabon or Gabonese Republic or Gambia or Gaza or Georgia Republic or Georgian Republic or Ghana or Gold Coast or Grenada or Guatemala or Guinea or Guinea-Bisau or Guam or Guiana or Guyana or Haiti or Honduras or India or Maldives or Indonesia or Iran or Iraq or Jamaica or Jordan or Kazakhstan or Kazakh or Kenya or Kiribati or Korea or Kosovo or Kyrgyzstan or Kirghizia or Kyrgyz Republic or Kirghiz or Kirgizstan or Lao PDR or Laos or Lebanon or Lesotho or Basutoland or Liberia or Libya or Macedonia or Madagascar or Malagasy Republic or Malaysia or Malaya or Malay or Sabah or Sarawak or Malawi or Nyasaland or Mali or Marshall Islands or Mauritania or Mauritius or Agalega Islands or Mexico or Micronesia or Middle East or Moldova or Moldovia or Moldovian or Mongolia or Montenegro or Morocco or Ifni or Mozambique or Myanmar or Myanma or Burma or Namibia or Nepal or Netherlands Antilles or Nicaragua or Niger or Nigeria or Pakistan or Palau or Palestine or Panama or Papua New Guinea or Paraguay or Peru or Philippines or Philipines or Phillipines or Phillippines or Romania or Rumania or Roumania or Rwanda or Ruanda or Saint Lucia or St Lucia or Saint Vincent or St Vincent or Grenadines or Samoa or Samoan Islands or Navigator Island or Navigator Islands or Sao Tome or Senegal or Serbia or Sierra Leone or Sri Lanka or Ceylon or Solomon Islands or Somalia or Sudan or Suriname or Surinam or Swaziland or Syria or Principe or South Sudan or Tajikistan or Tadzhikistan or Tadjikistan or Tadzhik or Tanzania or Thailand or Timor-Leste or Togo or

For peer review only - http://bmjopen.bmj.com/site/about/guidelines.xhtml

	Togolese Republic or Tonga or Tunisia or Turkey or Turkmenistan or Turkmen or Tuvalu or Uganda or Ukraine or Uzbekistan or Uzbek or Vanuatu or New Hebrides or Vietnam or Viet Nam or West Bank or Yemen or Zambia or Zimbabwe or Rhodesia).hw,ti,ab,cp.
18	((developing or less* developed or under developed or underdeveloped or middle income or low* income or underserved or underserved or deprived or poor*) adj (countr* or nation? or state? or population? or world)).ti,ab.
19	((developing or less* developed or under developed or underdeveloped or middle income or low* income) adj (economy or economies)).ti,ab.
20	(low* adj (gdp or gnp or gross domestic or gross national)).ti,ab.
21	(low adj3 middle adj3 countr*).ti,ab.
22	(lmic or lmics or third world or lami countr*).ti,ab.
23	transitional countr*.ti,ab.
24	15 or 16 or 17 or 18 or 19 or 20 or 21 or 22 or 23
25	5 and 14 and 24

For peer review only

CINAHL

#	Query
1	(MH "Anemia") OR (MH "Anemia, Hypochromic") OR (MH "Anemia, Iron Deficiency")
2	TI (anaemia or anemia) OR AB (((iron deficien* or hypochromic or microcytic) N5 (anaemia or anemia))) OR AB (((pregnan* or gestation* or maternal) and (anaemia or anemia)))
3	((MH "Pregnancy") OR (MH "Expectant Mothers")) AND (anaemia or anemia)
4	S1 OR S2 OR S3
5	(MH "Childbirth+") OR (MH "Labor+")
6	(MH "Delivery, Obstetric+")
7	TI (labor or labour or birth? or childbirth? or delivery or obstetric*) OR AB (((induction? or induced or inducing) N5 (labor or labour or birth? or childbirth? or delivery))) OR AB ((augment* N5 (labor or labour or birth? or childbirth? or delivery))) OR AB ((manage* N5 (labor or labour or birth? or childbirth? or delivery))) OR AB ((initiat* N5 (labor or labour or birth? or childbirth? or delivery)))
8	(MH "Oxytocics+")
9	(MH "Prostaglandins, Synthetic+")
10	TI (uterotonic? or oxytocin or prostaglandin? or misoprostol) OR AB (uterotonic? or oxytocin or prostaglandin? or misoprostol)
11	S5 OR S6 OR S7 OR S8 OR S9 OR S10
12	(MH "Developing Countries")
13	TI (Afghanistan or Albania or Algeria or Angola or American Samoa or Armenia or Armenian or Azerbaijan or Bangladesh or Benin or Byelarus or Byelorussian or Belarus or Belorussian or Belorussia or Belize or Bhutan or Bolivia or Bosnia or Herzegovina or Hercegovina or Botswana or Brazil or Brasil or Bulgaria or Burkina Faso or Burkina Fasso or Upper Volta or Burundi or Urundi or Cambodia or Khmer Republic or Kampuchea or Cameroon or Cameroons or Cameron or Camerons or Cape Verde or Central African Republic or Chad or China or Colombia or Comoros or Comoro Islands or Comores or Mayotte or Congo or Zaire or Costa Rica or Cote d'Ivoire or Ivory Coast or Cuba or Djibouti or French Somaliland or Dominica or Dominican Republic or East Timor or East Timur or Timor Leste or Ecuador or Egypt or United Arab Republic or El Salvador or Eritrea or Ethiopia or Fiji or Gabon or Gabonese Republic or Gambia or Gaza or Georgia Republic or Georgian Republic or Ghana or Gold Coast or Grenada or Guatemala or Guinea or Guinea-Bisau or Guam or Guiana or Guyana or Haiti or Honduras or India or Maldives or Indonesia or Iran or Iraq or Jamaica or Jordan or Kazakhstan or Kazakh or Kenya or Kiribati or Korea or Kosovo or Kyrgyzstan or Kirghizia or Kyrgyz Republic or Kirghiz or Kirgizstan or Lao PDR or Laos or Lebanon or Lesotho or Basutoland or Liberia or Libya or Macedonia or Madagascar or Malagasy Republic or Malaysia or Malaya or Malay or Sabah or Sarawak or Malawi or Nyasaland or Mali or Marshall Islands or Mauritania or Mauritius or Agalega Islands or Mexico or Micronesia or Middle East or Moldova or Moldovia or Moldovian or Mongolia or Montenegro or Morocco or Ifni or Mozambique or Myanmar or Myanma or Burma or Namibia or Nepal or Netherlands Antilles or Nicaragua or Niger or Nigeria or Pakistan or Palau or Palestine or Panama or Papua New Guinea or Paraguay or Peru or Philippines or Philipines or Phillipines or Phillippines or Romania or Rumania or Roumania or Rwanda or Ruanda or Saint Lucia or St Lucia or Saint Vincent or St Vincent or Grenadines or Samoa or Samoan Islands or Navigator Island or Navigator Islands or Sao Tome or Senegal or Serbia or Sierra Leone or Sri Lanka or Ceylon or Solomon Islands or Somalia or Sudan or Suriname or Surinam or Swaziland or Syria or Principe or South

For peer review only - http://bmjopen.bmj.com/site/about/guidelines.xhtml

	Sudan or Tajikistan or Tadzhikistan or Tadjikistan or Tadzhik or Tanzania or Thailand or Timor-Leste or Togo or Togolese Republic or Tonga or Tunisia or Turkey or Turkmenistan or Turkmen or Tuvalu or Uganda or Ukraine or Uzbekistan or Uzbek or Vanuatu or New Hebrides or Vietnam or Viet Nam or West Bank or Yemen or Zambia or Zimbabwe or Rhodesia) OR AB (Afghanistan or Albania or Algeria or Angola or American Samoa or Armenia or Armenian or Azerbaijan or Bangladesh or Benin or Byelarus or Byelorussian or Belarus or Belorussian or Belorussia or Belize or Bhutan or Bolivia or Bosnia or Herzegovina or Hercegovina or Botswana or Brazil or Brasil or Bulgaria or Burkina Faso or Burkina Fasso or Upper Volta or Burundi or Urundi or Cambodia or Khmer Republic or Kampuchea or Cameroon or Cameroons or Cameron or Camerons or Cape Verde or Central African Republic or Chad or China or Colombia or Comoros or Comoro Islands or Comores or Mayotte or Congo or Zaire or Costa Rica or Cote d'Ivoire or Ivory Coast or Cuba or Djibouti or French Somaliland or Dominica or Dominican Republic or East Timor or East Timur or Timor Leste or Ecuador or Egypt or United Arab Republic or El Salvador or Eritrea or Ethiopia or Fiji or Gabon or Gabonese Republic or Gambia or Gaza or Georgia Republic or Georgian Republic or Ghana or Gold Coast or Grenada or Guatemala or Guinea or Guinea-Bisau or Guam or Guiana or Guyana or Haiti or Honduras or India or Maldives or Indonesia or Iran or Iraq or Jamaica or Jordan or Kazakhstan or Kazakh or Kenya or Kiribati or Korea or Kosovo or Kyrgyzstan or Kirghizia or Kyrgyz Republic or Kirghiz or Kirgizstan or Lao PDR or Laos or Lebanon or Lesotho or Basutoland or Liberia or Libya or Macedonia or Madagascar or Malagasy Republic or Malaysia or Malaya or Malay or Sabah or Sarawak or Malawi or Nyasaland or Mali or Marshall Islands or Mauritania or Mauritius or Agalega Islands or Mexico or Micronesia or Middle East or Moldova or Moldovia or Moldovian or Mongolia or Montenegro or Morocco or Ifni or Mozambique or Myanmar or Myanma or Burma or Namibia or Nepal or Netherlands Antilles or Nicaragua or Niger or Nigeria or Pakistan or Palau or Palestine or Panama or Papua New Guinea or Paraguay or Peru or Philippines or Philipines or Phillipines or Phillippines or Romania or Rumania or Roumania or Rwanda or Ruanda or Saint Lucia or St Lucia or Saint Vincent or St Vincent or Grenadines or Samoa or Samoan Islands or Navigator Island or Navigator Islands or Sao Tome or Senegal or Serbia or Sierra Leone or Sri Lanka or Ceylon or Solomon Islands or Somalia or Sudan or Suriname or Surinam or Swaziland or Syria or Principe or South Sudan or Tajikistan or Tadzhikistan or Tadjikistan or Tadzhik or Tanzania or Thailand or Timor-Leste or Togo or Togolese Republic or Tonga or Tunisia or Turkey or Turkmenistan or Turkmen or Tuvalu or Uganda or Ukraine or Uzbekistan or Uzbek or Vanuatu or New Hebrides or Vietnam or Viet Nam or West Bank or Yemen or Zambia or Zimbabwe or Rhodesia) OR MW (Afghanistan or Albania or Algeria or Angola or American Samoa or Armenia or Armenian or Azerbaijan or Bangladesh or Benin or Byelarus or Byelorussian or Belarus or Belorussian or Belorussia or Belize or Bhutan or Bolivia or Bosnia or Herzegovina or Hercegovina or Botswana or Brazil or Brasil or Bulgaria or Burkina Faso or Burkina Fasso or Upper Volta or Burundi or Urundi or Cambodia or Khmer Republic or Kampuchea or Cameroon or Cameroons or Cameron or Camerons or Cape Verde or Central African Republic or Chad or China or Colombia or Comoros or Comoro Islands or Comores or Mayotte or Congo or Zaire or Costa Rica or Cote d'Ivoire or Ivory Coast or Cuba or Djibouti or French Somaliland or Dominica or Dominican Republic or East Timor or East Timur or Timor Leste or Ecuador or Egypt or United Arab Republic or El Salvador or Eritrea or Ethiopia or Fiji or Gabon or Gabonese Republic or Gambia or Gaza or Georgia Republic or Georgian Republic or Ghana or Gold Coast or Grenada or Guatemala or Guinea or Guinea-Bisau or Guam or Guiana or Guyana or Haiti or Honduras or India or Maldives or Indonesia or Iran or Iraq or Jamaica or Jordan or Kazakhstan or Kazakh or Kenya or Kiribati or Korea or Kosovo or Kyrgyzstan or Kirghizia or Kyrgyz Republic or Kirghiz or Kirgizstan or Lao PDR or Laos or Lebanon or Lesotho or Basutoland

For peer review only - http://bmjopen.bmj.com/site/about/guidelines.xhtml

	or Liberia or Libya or Macedonia or Madagascar or Malagasy Republic or Malaysia or Malaya or Malay or Sabah or Sarawak or Malawi or Nyasaland or Mali or Marshall Islands or Mauritania or Mauritius or Agalega Islands or Mexico or Micronesia or Middle East or Moldova or Moldovia or Moldovian or Mongolia or Montenegro or Morocco or Ifni or Mozambique or Myanmar or Myanma or Burma or Namibia or Nepal or Netherlands Antilles or Nicaragua or Niger or Nigeria or Pakistan or Palau or Palestine or Panama or Papua New Guinea or Paraguay or Peru or Philippines or Philipines or Phillipines or Phillippines or Romania or Rumania or Roumania or Rwanda or Ruanda or Saint Lucia or St Lucia or Saint Vincent or St Vincent or Grenadines or Samoa or Samoan Islands or Navigator Island or Navigator Islands or Sao Tome or Senegal or Serbia or Sierra Leone or Sri Lanka or Ceylon or Solomon Islands or Somalia or Sudan or Suriname or Surinam or Swaziland or Syria or Principe or South Sudan or Tajikistan or Tadzhikistan or Tadjikistan or Tadzhik or Tanzania or Thailand or Timor-Leste or Togo or Togolese Republic or Tonga or Tunisia or Turkey or Turkmenistan or Turkmen or Tuvalu or Uganda or Ukraine or Uzbekistan or Uzbek or Vanuatu or New Hebrides or Vietnam or Viet Nam or West Bank or Yemen or Zambia or Zimbabwe or Rhodesia) OR AF (Afghanistan or Albania or Algeria or Angola or American Samoa or Armenia or Armenian or Azerbaijan or Bangladesh or Benin or Byelarus or Byelorussian or Belarus or Belorussian or Belorussia or Belize or Bhutan or Bolivia or Bosnia or Herzegovina or Hercegovina or Botswana or Brazil or Brasil or Bulgaria or Burkina Faso or Burkina Fasso or Upper Volta or Burundi or Urundi or Cambodia or Khmer Republic or Kampuchea or Cameroon or Cameroons or Cameron or Camerons or Cape Verde or Central African Republic or Chad or China or Colombia or Comoros or Comoro Islands or Comores or Mayotte or Congo or Zaire or Costa Rica or Cote d'Ivoire or Ivory Coast or Cuba or Djibouti or French Somaliland or Dominica or Dominican Republic or East Timor or East Timur or Timor Leste or Ecuador or Egypt or United Arab Republic or El Salvador or Eritrea or Ethiopia or Fiji or Gabon or Gabonese Republic or Gambia or Gaza or Georgia Republic or Georgian Republic or Ghana or Gold Coast or Grenada or Guatemala or Guinea or Guinea-Bisau or Guam or Guiana or Guyana or Haiti or Honduras or India or Maldives or Indonesia or Iran or Iraq or Jamaica or Jordan or Kazakhstan or Kazakh or Kenya or Kiribati or Korea or Kosovo or Kyrgyzstan or Kirghizia or Kyrgyz Republic or Kirghiz or Kirgizstan or Lao PDR or Laos or Lebanon or Lesotho or Basutoland or Liberia or Libya or Macedonia or Madagascar or Malagasy Republic or Malaysia or Malaya or Malay or Sabah or Sarawak or Malawi or Nyasaland or Mali or Marshall Islands or Mauritania or Mauritius or Agalega Islands or Mexico or Micronesia or Middle East or Moldova or Moldovia or Moldovian or Mongolia or Montenegro or Morocco or Ifni or Mozambique or Myanmar or Myanma or Burma or Namibia or Nepal or Netherlands Antilles or Nicaragua or Niger or Nigeria or Pakistan or Palau or Palestine or Panama or Papua New Guinea or Paraguay or Peru or Philippines or Philipines or Phillipines or Phillippines or Romania or Rumania or Roumania or Rwanda or Ruanda or Saint Lucia or St Lucia or Saint Vincent or St Vincent or Grenadines or Samoa or Samoan Islands or Navigator Island or Navigator Islands or Sao Tome or Senegal or Serbia or Sierra Leone or Sri Lanka or Ceylon or Solomon Islands or Somalia or Sudan or Suriname or Surinam or Swaziland or Syria or Principe or South Sudan or Tajikistan or Tadzhikistan or Tadjikistan or Tadzhik or Tanzania or Thailand or Timor-Leste or Togo or Togolese Republic or Tonga or Tunisia or Turkey or Turkmenistan or Turkmen or Tuvalu or Uganda or Ukraine or Uzbekistan or Uzbek or Vanuatu or New Hebrides or Vietnam or Viet Nam or West Bank or Yemen or Zambia or Zimbabwe or Rhodesia)

For peer review only - http://bmjopen.bmj.com/site/about/guidelines.xhtml

14	(((developing or less* developed or under developed or underdeveloped or middle income or low* income or underserved or under served or deprived or poor*) N1 (countr* or nation? or state? or population? or world))) OR (((developing or less* developed or under developed or underdeveloped or middle income or low* income) N1 (economy or economies))) OR ((low* N1 (gdp or gnp or gross domestic or gross national))) OR (low N3 middle N3 countr*) OR (lmic or lmics or third world or lami countr*) OR transitional countr*
15	S12 OR S13 OR S14
16	S4 AND S11 AND S15

Cochrane Central Register of Controlled Trials

ID	Search
#1	MeSH descriptor: [Anemia, Iron-Deficiency] explode all trees
#2	MeSH descriptor: [Anemia] this term only
#3	MeSH descriptor: [Anemia, Hypochromic] this term only
#4	anaemia or anemia:ti (Word variations have been searched)
#5	((iron deficien* or hypochromic or microcytic) near (anaemia or anemia)):ti,ab,kw (Word variations have been searched)
#6	((pregnan* or gestation* or maternal) and (anaemia or anemia)):ti,ab,kw (Word variations have been searched)
#7	#1 or #2 or #3 or #4 or #5 or #6
#8	MeSH descriptor: [Labor, Obstetric] explode all trees
#9	MeSH descriptor: [Parturition] explode all trees
#10	MeSH descriptor: [Delivery, Obstetric] explode all trees
#11	labor or labour or birth? or childbirth? or delivery or obstetric*:ti (Word variations have been searched)
#12	((induction? or induced or inducing) near (labor or labour or birth? or childbirth? or delivery)):ti,ab,kw (Word variations have been searched)
#13	(augment* near (labor or labour or birth? or childbirth? or delivery)):ti,ab,kw (Word variations have been searched)
#14	(manage* near (labor or labour or birth? or childbirth? or delivery)):ti,ab,kw (Word variations have been searched)
#15	(initiat* near (labor or labour or birth? or childbirth? or delivery)):ti,ab,kw (Word variations have been searched)
#16	MeSH descriptor: [Oxytocin] explode all trees
#17	MeSH descriptor: [Prostaglandins E, Synthetic] explode all trees
#18	(uterotonic? or oxytocin or prostaglandin? or misoprostol):ti,ab,kw (Word variations have been searched)
#19	#8 or #9 or #10 or #11 or #12 or #13 or #14 or #15 or #16 or #17 or #18
#20	#7 and #19

For peer review only

For peer review only - http://bmjopen.bmj.com/site/about/guidelines.xhtml

Global Health Library

(anaemia OR anemia) AND (labour OR labor OR birth OR childbirth OR delivery OR obstetrics OR obstetric OR uterotonic OR uterotonics OR oxytocin OR prostaglandin OR prostaglandins OR misoprostol)
(iron deficiency anaemia OR iron deficiency anemia) AND (labour OR labor OR birth OR childbirth OR delivery OR obstetrics OR obstetric OR uterotonic OR uterotonics OR oxytocin OR prostaglandin OR prostaglandins OR misoprostol)
(hypochromic anaemia OR hypochromic anemia) AND (labour OR labor OR birth OR childbirth OR delivery OR obstetrics OR obstetric OR uterotonic OR uterotonics OR oxytocin OR prostaglandin OR prostaglandins OR misoprostol)
(microcytic anaemia OR microcytic anemia) AND (labour OR labor OR birth OR childbirth OR delivery OR obstetrics OR obstetric OR uterotonic OR uterotonics OR oxytocin OR prostaglandin OR prostaglandins OR misoprostol)

Dissertations & Theses (Global full text plus UK and Ireland abstracts)

Set	Search
S8	S1 AND S6
S6	ti(pregnancy OR pregnant)
S5	S1 AND S4
S4	S2 OR S3
S3	ti(uterotonic? or oxytocin or prostaglandin? or misoprostol)
S2	ti(labor or labour or birth? or childbirth? or delivery or obstetric*)
S1	ti(anaemia OR anemia)

Trials registers: Clinical trials.gov (https://clinicaltrials.gov/) and WHO ICTRP (http://apps.who.int/trialsearch/)

**Clinicaltrials.gov**
Condition=anaemia OR anemia AND Intervention=labour OR labor OR birth OR childbirth OR delivery OR obstetric OR obstetrics OR oxytocin OR prostaglandin OR prostaglandins OR misoprostol
Condition=anaemia OR anemia AND Title=labour OR labor OR birth OR childbirth OR delivery OR obstetric OR obstetrics OR oxytocin OR prostaglandin OR prostaglandins OR misoprostol
Condition=anaemia OR anemia AND Title=labour OR labor OR birth OR childbirth OR delivery OR obstetric OR obstetrics OR oxytocin OR prostaglandin OR prostaglandins OR misoprostol

**WHO Search:**
Condition=anaemia OR anemia AND Intervention=labour OR labor OR birth OR childbirth OR delivery OR obstetric OR obstetrics OR oxytocin OR prostaglandin OR prostaglandins OR misoprostol
Title=(anaemia OR anemia) AND Intervention=(labour OR labor OR birth OR childbirth OR delivery OR obstetric OR obstetrics OR oxytocin OR prostaglandin OR prostaglandins OR misoprostol)
Condition=anaemia OR anemia AND Title=labour OR labor OR birth OR childbirth OR delivery OR obstetric OR obstetrics OR oxytocin OR prostaglandin OR prostaglandins OR misoprostol

For peer review only - http://bmjopen.bmj.com/site/about/guidelines.xhtml
For peer review only

PRISMA 2009 Checklist

Section/topic	#	Checklist item	Reported on page #
**TITLE**			
Title	1	Identify the report as a systematic review, meta-analysis, or both.	1
**ABSTRACT**			
Structured summary	2	Provide a structured summary including, as applicable: background; objectives; data sources; study eligibility criteria, participants, and interventions; study appraisal and synthesis methods; results; limitations; conclusions and implications of key findings; systematic review registration number.	2
**INTRODUCTION**			
Rationale	3	Describe the rationale for the review in the context of what is already known.	4
Objectives	4	Provide an explicit statement of questions being addressed with reference to participants, interventions, comparisons, outcomes, and study design (PICOS).	4
**METHODS**			
Protocol and registration	5	Indicate if a review protocol exists, if and where it can be accessed (e.g., Web address), and, if available, provide registration information including registration number.	5
Eligibility criteria	6	Specify study characteristics (e.g., PICOS, length of follow-up) and report characteristics (e.g., years considered, language, publication status) used as criteria for eligibility, giving rationale.	5
Information sources	7	Describe all information sources (e.g., databases with dates of coverage, contact with study authors to identify additional studies) in the search and date last searched.	5
Search	8	Present full electronic search strategy for at least one database, including any limits used, such that it could be repeated.	6 and S1
Study selection	9	State the process for selecting studies (i.e., screening, eligibility, included in systematic review, and, if applicable, included in the meta-analysis).	5, 6, 7
Data collection process	10	Describe method of data extraction from reports (e.g., piloted forms, independently, in duplicate) and any processes for obtaining and confirming data from investigators.	7, 8
Data items	11	List and define all variables for which data were sought (e.g., PICOS, funding sources) and any assumptions and simplifications made.	7, 8
Risk of bias in individual studies	12	Describe methods used for assessing risk of bias of individual studies (including specification of whether this was done at the study or outcome level), and how this information is to be used in any data synthesis.	8
Summary measures	13	State the principal summary measures (e.g., risk ratio, difference in means).	8
Synthesis of results	14	Describe the methods of handling data and combining results of studies, if done, including measures of consistency (e.g., I^2) for each meta-analysis.	8

For peer review only - http://bmjopen.bmj.com/site/about/guidelines.xhtml

PRISMA 2009 Checklist

Section/topic	#	Checklist item	Reported on page #
Risk of bias across studies	15	Specify any assessment of risk of bias that may affect the cumulative evidence (e.g., publication bias, selective reporting within studies).	8
Additional analyses	16	Describe methods of additional analyses (e.g., sensitivity or subgroup analyses, meta-regression), if done, indicating which were pre-specified.	8
**RESULTS**			
Study selection	17	Give numbers of studies screened, assessed for eligibility, and included in the review, with reasons for exclusions at each stage, ideally with a flow diagram.	8, 9
Study characteristics	18	For each study, present characteristics for which data were extracted (e.g., study size, PICOS, follow-up period) and provide the citations.	9
Risk of bias within studies	19	Present data on risk of bias of each study and, if available, any outcome level assessment (see item 12).	9
Results of individual studies	20	For all outcomes considered (benefits or harms), present, for each study: (a) simple summary data for each intervention group (b) effect estimates and confidence intervals, ideally with a forest plot.	9 and Table-1 (10)
Synthesis of results	21	Present results of each meta-analysis done, including confidence intervals and measures of consistency.	9
Risk of bias across studies	22	Present results of any assessment of risk of bias across studies (see Item 15).	NA
Additional analysis	23	Give results of additional analyses, if done (e.g., sensitivity or subgroup analyses, meta-regression [see Item 16]).	NA
**DISCUSSION**			
Summary of evidence	24	Summarize the main findings including the strength of evidence for each main outcome; consider their relevance to key groups (e.g., healthcare providers, users, and policy makers).	11
Limitations	25	Discuss limitations at study and outcome level (e.g., risk of bias), and at review-level (e.g., incomplete retrieval of identified research, reporting bias).	11
Conclusions	26	Provide a general interpretation of the results in the context of other evidence, and implications for future research.	12, 13
**FUNDING**			
Funding	27	Describe sources of funding for the systematic review and other support (e.g., supply of data); role of funders for the systematic review.	16

From: Moher D, Liberati A, Tetzlaff J, Altman DG, The PRISMA Group (2009). Preferred Reporting Items for Systematic Reviews and Meta-Analyses: The PRISMA Statement. PLoS Med 6(7): e1000097. doi:10.1371/journal.pmed1000097

For more information, visit: www.prisma-statement.org.

For peer review only - http://bmjopen.bmj.com/site/about/guidelines.xhtml

BMJ Open

A systematic review to investigate the safety of induction and augmentation of labour among pregnant women with iron deficiency anaemia.

Journal:	*BMJ Open*
Manuscript ID	bmjopen-2018-021793.R2
Article Type:	Research
Date Submitted by the Author:	04-Oct-2018
Complete List of Authors:	Bunch, Kathryn; University of Oxford, National Perinatal Epidemiology Unit, Nuffield Department of Population Health<br>Roberts, Nia; University of Oxford, UK, Bodleian Health Care Libraries,<br>Knight, Marian; National Perinatal Epidemiology Unit<br>Nair, Manisha; University of Oxford, NPEU, Nuffield Department of Population Health
**Primary Subject Heading**:	Obstetrics and gynaecology
Secondary Subject Heading:	Public health, Global health
Keywords:	Pregnant women, Anaemia < HAEMATOLOGY, induction of labour, augmentation of labour, post-partum haemorrhage

SCHOLARONE™
Manuscripts

TITLE PAGE

A systematic review to investigate the safety of induction and augmentation of labour among pregnant women with iron deficiency anaemia

AUTHORS

1. Kathryn Bunch
   National Perinatal Epidemiology Unit, Nuffield Department of Population Health, University of Oxford, Richard Doll Building, Old Road Campus, Headington, Oxford, OX3 7LF, UK. Email: kathryn.bunch@npeu.ox.ac.uk
2. Nia Roberts
   Bodleian Libraries, University of Oxford, Knowledge Centre, Old Road Research Building, off Roosevelt Drive, Headington, Oxford, OX3 7DQ, UK.
   Email: nia.roberts@bodleian.ox.ac.uk
3. Marian Knight
   National Perinatal Epidemiology Unit, Nuffield Department of Population Health, University of Oxford, Richard Doll Building, Old Road Campus, Headington, Oxford, OX3 7LF, UK. Email: marian.knight@npeu.ox.ac.uk
4. Manisha Nair
   National Perinatal Epidemiology Unit, Nuffield Department of Population Health, University of Oxford, Richard Doll Building, Old Road Campus, Headington, Oxford, OX3 7LF, UK. Email: manisha.nair@npeu.ox.ac.uk

Corresponding author

Manisha Nair
National Perinatal Epidemiology Unit, Nuffield Department of Population Health,
University of Oxford, Richard Doll Building, Old Road Campus, Headington, Oxford, OX3 7LF, UK.
Email: manisha.nair@npeu.ox.ac.uk
Phone: +44 (0)1865617820

For peer review only

For peer review only - http://bmjopen.bmj.com/site/about/guidelines.xhtml

ABSTRACT

Objective: To conduct a systematic review to investigate the safety of induction and/ or augmentation of labour compared with spontaneous-onset normal labour among pregnant women with iron-deficiency anaemia.

Design: Systematic review.

Setting: Studies from all countries, worldwide.

Population: Pregnant women with iron-deficiency anaemia at labour and delivery.

Intervention: Any intervention related to induction and/ or augmentation of labour.

Outcome measures: Primary: Postpartum haemorrhage (PPH), heart failure and maternal death. Secondary: Emergency caesarean section, hysterectomy, admission to intensive care unit.

Method: We searched 10 databases, including Medline and Embase, from database inception to 01 October 2018. We included all study designs except cross-sectional studies without a comparator group, case reports, case series, ecological studies, and expert opinion. The searches were conducted by a healthcare librarian and two authors independently screened and reviewed the studies. We used the GRADE approach to ascertain risk of bias and conducted a narrative synthesis.

Results: We identified 3217 journal articles, 223 conference papers, 45 dissertations, and 218 registered trials. Ten articles were included for full-text review and only one was found to fulfil the eligibility criteria. This was a retrospective cohort study from India which showed that pregnant women with moderate and severe anaemia could have an increased risk of PPH if they underwent induction and/or augmentation of labour, but the evidence was weak (graded as 'High risk of bias').

Conclusion: The best approach is to prevent anaemia, but a large number of women in low-to-middle-income countries present with severe anaemia during labour. In such women, appropriate peripartum management could prevent complications and death. Our review showed that at present we do not know if induction and augmentation of labour is safe in pregnant women with iron deficiency anaemia and further research is required.

ARTICLE SUMMARY

Strengths and limitations of this study

- This is the first systematic review conducted to examine the evidence of the safety of induction and augmentation of labour in pregnant women with iron-deficiency anaemia.
- A rigorous search method was used to identify published and unpublished studies in any language from all countries, globally, using 10 databases from their inception till date.
- Despite a rigorous search over a period of two years, only one study was found.

For peer review only - http://bmjopen.bmj.com/site/about/guidelines.xhtml

Key words: Pregnant women; anaemia; iron-deficiency; induction of labour; augmentation of labour; post-partum haemorrhage.

Word count: 2706

For peer review only

INTRODUCTION

Iron-deficiency anaemia during pregnancy is defined as a haemoglobin concentration less than 11g/dl (110g/l) and is a major cause of maternal morbidity and mortality in low and middle income countries (LMICs)[1]. An estimated 42% of pregnant women have anaemia, globally[1], but the prevalence is reported to be as high as 70-80% in countries such as India[2]. Pregnant women with iron deficiency anaemia are at a higher risk of postpartum haemorrhage (PPH)[3] and heart failure[4] during pregnancy and childbirth. Clearly the ideal approach would be to prevent anaemia or treat it during the antenatal period, but a large number of pregnant women in LMICs present with severe anaemia either at the point of delivery or in the late third trimester of pregnancy[5 6] when there is little scope for prevention or iron treatment.

A systematic review showed that active management of the third stage of labour reduces the risk of PPH among pregnant women with haemoglobin concentrations <9g/dl, although the quality of evidence was low[7]. There is no clear evidence about the risks and benefits of induction and augmentation of labour during the first and second stages of labour among pregnant women with iron deficiency anaemia. A recent study showed that pregnant women with moderate-severe anaemia (haemoglobin <10g/dl) who underwent induction of labour were at an increased risk of PPH[8]. A few small studies have suggested that women with severe anaemia are more likely to have uterine atony due to impaired transport of oxygen rich haemoglobin to the uterus[3 9], but there is no strong evidence. This impaired transport of oxygen due to low haemoglobin could also result in early fatigue of the uterus following use of uterotonics [10-12] resulting in uterine atony and subsequent PPH. The aim of this systematic review was to investigate the safety of induction and/ or augmentation of labour compared with spontaneous-onset normal labour in pregnant women with anaemia.

For peer review only - http://bmjopen.bmj.com/site/about/guidelines.xhtml

METHOD

Study design: Systematic review of published and unpublished literature. The study was registered in PROSPERO (International prospective register of systematic reviews): CRD42015032421.

Eligibility criteria

Types of studies: We included all comparative study designs. Cross-sectional studies without a comparator group, case reports, case series, ecological studies, and expert opinion pieces were excluded.

Types of participant: Our search was restricted to pregnant women with iron deficiency anaemia >20 weeks gestation at labour and delivery. We excluded pregnant women with anaemia due to haemoglobinopathies.

Setting: We included studies from all countries.

We searched for published and unpublished literature in any language from the inception of the databases until 01 October 2018.

Information sources

The following electronic databases were searched for published literature: Medline, Embase, CINAHL (Cumulative Index to Nursing and Allied Health Literature), Global Health (OvidSP), Global Health Library, and Cochrane Central Register of Controlled Trials. Unpublished or grey literature searches were restricted to dissertations, reports and conference proceedings using two recognized databases: 'Index to theses' and 'Proquest Dissertations & Theses'. In addition, we looked for ongoing unpublished trials by searching trial registers such as www.ClinicalTrials.gov and the World Health Organisation (WHO) International Clinical Trials Registry Platform (www.who.int/trialsearch/). We also planned to hand search the reference lists of included studies.

For peer review only

Search strategy

NR carried out the electronic database searches. An EndNote bibliographic database was used to manage references, identify duplicates and share references among the reviewers. The screening was carried out independently by two reviewers (KB and MN) in two stages – (i) screening of titles and abstracts based on the pre-specified inclusion and exclusion criteria; (ii) screening of full-texts of the papers included during stage-1. Any disagreements on inclusion of studies were discussed and, where possible, resolved by consensus after referring to the review protocol or a third reviewer (MK). A record of decisions made for each article was maintained. The search strategy was piloted by applying the inclusion and exclusion criteria to a sample of papers to check its reliability in correctly classifying the studies. The process helped to refine the selection criteria and the search strategies (provided in the supplementary document: S1 Text). Since the focus of this review was on the safety of induction and/ or augmentation of labour compared with spontaneous-onset normal labour in pregnant women with anaemia, the key components of our search were: (i) population – pregnant women with anaemia and (ii) intervention - induction and/ or augmentation of labour. We did not restrict the breadth of the search by specifying any outcome. We hand-searched key papers and additional reference linking was done by the authors. The PRISMA flow chart was used to summarise the selection process.

Types of interventions

We searched for studies that included any intervention related to induction of labour, augmentation of labour, and combined induction and augmentation of labour; with and without active management of the third stage in women with iron deficiency anaemia. Key definitions of interventions and outcomes are provided in Box-1.

Comparisons

Pregnant women with anaemia who had spontaneous-onset normal labour.

For peer review only - http://bmjopen.bmj.com/site/about/guidelines.xhtml

Types of outcome measures

Adverse maternal outcomes during labour, delivery and within 24 hours of childbirth.

Primary outcomes:

- Primary postpartum haemorrhage (defined in Box-1) or requiring therapeutic uterotonics or transfusion of blood or blood products during labour or delivery up to 24 hours after delivery.
- Heart failure during labour or delivery or up to 24 hours after delivery.
- Maternal death.

Secondary outcomes:

- Emergency caesarean section.
- Hysterectomy.
- Admission to intensive care unit.
- Any other adverse maternal outcome

Box-1: Key definitions

- Iron deficiency anaemia among pregnant women is defined as a haemoglobin concentration of less than 11g/dl and can be categorised as mild anaemia (haemoglobin 10-10.9g/dl), moderate anaemia (haemoglobin 7-9·9 g/dl) and severe anaemia (haemoglobin <7 g/dl)[13].
- Induction of labour is defined as 'the initiation of labour by artificial means prior to its spontaneous onset at a viable gestational age, with the aim of achieving vaginal delivery in a pregnant woman with intact membranes'[14].
- Augmentation of labour is the process of stimulating the uterus to increase the frequency, duration and intensity of contractions after the onset of labour through intravenous oxytocin infusion and/ or artificial rupture of membranes[15].
- Active management of the third stage of labour is 'a prophylactic intervention composed of a package of three components or steps: 1) administration of a uterotonic, preferably oxytocin, immediately after birth of the baby; 2) controlled cord traction to deliver the placenta; and 3) massage of the uterine fundus after the placenta is delivered.' New recommendations clarify that administration of a uterotonic is the key component, the other two being optional[16].
- A maternal death is the death of a woman while pregnant or within 42 days of the end of pregnancy, irrespective of the duration and the site of the pregnancy, from any cause related to or aggravated by the pregnancy or its management, but not from accidental or incidental causes[17].
- Primary postpartum haemorrhage is defined as a pregnant women with a blood

For peer review only - http://bmjopen.bmj.com/site/about/guidelines.xhtml

loss of 500 ml or more from the genital tract within 24 hours of giving birth[16].

Data collection and analysis

The data extraction forms and coding sheets were developed after reviewing a subset of sample papers followed by testing the tools in the remaining sub-set to ensure that the tools captured all relevant information[18]. Data extraction from included studies was performed independently by two reviewers (MN and KB) and we planned to measure the level of inter-reviewer agreement using a Kappa statistic (a measure of chance-corrected agreement)[19]. As with selection of studies, we planned to resolve any disagreement through consensus or through arbitration by a third reviewer (MK). We planned to note the corrections/ amendments in the data extraction forms.

Risk of bias (quality) assessment

We used the GRADE approach to ascertain risk of bias and categorise studies into 'low risk', 'high risk' and 'unclear risk', and judgement was substantiated by a text description. We planned to assess publication bias through visual inspection and by conducting tests for asymmetry of funnel plots if at least five studies were included in the meta-analysis.

Strategy for data synthesis

We proposed to use both quantitative and narrative synthesis. Depending on the presence or absence of statistical, clinical and methodological heterogeneity of the included studies, a random-effects model or a fixed-effects model would be used to generate pooled estimates of the outcome. If clinical and methodological heterogeneity are observed, sensitivity analyses would be conducted to understand their effects on the pooled estimates. We also planned to conduct subgroup analysis to ascertain if the maternal outcomes of induction of labour and/ or augmentation of labour vary according to the severity of iron deficiency anaemia among pregnant women.

Patient and Public Involvement: Not applicable since this is a systematic review.

For peer review only - http://bmjopen.bmj.com/site/about/guidelines.xhtml

RESULTS

The search of published and unpublished studies identified 2071 journal articles from LMICs and 1146 articles from high income countries (HICs), 223 conference papers, 45 dissertations, and 218 registered trials after removing duplicates. Countries were categorised into income groups (HIC and LMIC) based on the World Bank's classification for 2017-18 (https://datahelpdesk.worldbank.org/knowledgebase/articles/906519-world-bank-country-and-lending-groups). After screening the title and abstracts of all articles, conference proceedings and dissertations against inclusion and exclusion criteria, eight journal articles were included for full-text review from LMICs and two from HICs.

Six out of the eight studies from LMICs did not meet the inclusion criteria. The studies either did not include pregnant women with anaemia or did not include any information about the interventions of interest. One study from a hospital in India examined separately the proportion of women who underwent induction of labour and the proportion who had a postpartum haemorrhage among a total of 447 pregnant women with varying degrees of anaemia [9]. The study showed that a higher proportion of women who had severe anaemia underwent induction of labour compared with women who did not have anaemia[9]. The proportion of women who had a postpartum haemorrhage was also higher in the severe anaemia group[9]. We contacted the authors for data on postpartum haemorrhage sub-divided by induction of labour versus spontaneous vaginal delivery for each group of women with varying degrees of anaemia, but were unable to get any response despite three attempts.

The only study found to fulfil the inclusion and exclusion criteria was our previous study that led to the hypothesis for this systematic review. This was a retrospective cohort study among 1,007 pregnant women from five teaching hospitals in India. It showed that compared with women who have a normal haemoglobin concentration, women who have moderate-severe anaemia have a significantly higher odds of postpartum haemorrhage and notably, the odds increased 17-fold among women with moderate and severe anaemia who underwent induction of labour[8]. However, the evidence from the study was not strong due to a 'high risk'

For peer review only - http://bmjopen.bmj.com/site/about/guidelines.xhtml

of bias assessed using the GRADE classification. The two studies from HICs were published in Polish, but none were found to meet the inclusion criteria. The reasons for exclusion are shown in Table-1 and a PRISMA flow-chart showing the selection process is presented in Figure-1.

Table-1: Reasons for exclusion of studies after full-text review

Study reference	Reasons for exclusion
**Studies from LMICs**	
Tee ES, Kandiah M, Ali J, et al. (1984)[20]	This was a descriptive study of the prevalence and outcomes of nutritional anaemia among 309 pregnant women in a maternity hospital in Malaysia. Thus it did not fit the inclusion criteria for study design, interventions and outcomes.
Tsu VD. (1993)[21]	Pregnant women who were induced with oxytocin before delivery were excluded, hence no intervention or comparator.
Phillip H, Fletcher H, Reid M. (2004)[22]	Pregnant women with anaemia were excluded from the study.
Kavle JA, Stoltzfus RJ, Witter F, et al.(2008)[3]	Did not include any information on the intervention of interest – induction and/ or augmentation of labour compared with spontaneous vaginal delivery. The study showed that ergometrine and/ or oxytocins in the third stage of labour were significantly associated with increased odds of postpartum haemorrhage.
Wang X, Tan H, Zhou S, et al (2014)[23]	This was a case-control study that examined the risk factors for PPH. Anaemia was found to be an independent risk factors for PPH, but management of labour and delivery was not examined.
Doncev V (1974)[24]	This study compared the proportions of maternal and fetal complications between 138 pregnant women with anaemia and 300 pregnant women without anaemia. Higher proportions of women with anaemia had preterm delivery, prolonged labour, uterine inertia, and birth asphyxia compared with women with no anaemia. The study did not fit the inclusion criterion for interventions.
**Studies from HICs**	
Salwa M (1975)[25]	This study examined complications during labour and delivery in 309 pregnant women and reported a higher prevalence of PPH in women with anaemia (11-75%) compared with women with no anaemia (8.2%). The study did not fit the inclusion criterion for interventions.
Daraz B (1978)[26]	This cross-sectional study of 2128 pregnant women during labour showed that the average duration of the first and second stages of labour increased with a decrease in the concentration of haemoglobin, but

For peer review only - http://bmjopen.bmj.com/site/about/guidelines.xhtml

	the association was statistically significant for multiparous women only. The study did not fit the inclusion criteria for study design, interventions and outcomes.

LMICs–Low and middle income countries; HICs–High income countries; PPH–Postpartum haemorrhage

DISCUSSION

This systematic search of published and unpublished studies in 10 databases using key words and search criteria found only one study which showed that pregnant women with moderate and severe anaemia had an increased risk of postpartum haemorrhage (PPH) if they underwent induction and/or augmentation of labour, but the evidence was not strong. We did not find any other study that examined the clinical management of anaemic pregnant women during labour and delivery, specifically the association of induction and/or augmentation of labour with adverse maternal outcomes.

This is the first systematic review conducted to examine the evidence of the safety of induction and augmentation of labour compared with spontaneous-onset normal labour in pregnant women with iron-deficiency anaemia. A broad rigorous search of published and unpublished studies in any language from all countries, globally, using 10 databases from their inception till date, identified only one study that fulfilled the eligibility criteria. The results of this review are therefore inconclusive. Despite several attempts we could not obtain any further information for another study from India.

While some studies investigating the risk factors for PPH found that induction of labour[27 28], prolonged second stage of labour[28] and anaemia[28] were independently associated with PPH, others suggest that anaemia is associated with prolonged labour which in turn increases the risk of PPH[3 9]. A recent study from Egypt showed that compared with women who have normal haemoglobin or mild anaemia, pregnant women with moderate to severe anaemia have a higher level of nitric oxide which signals smooth muscle relaxation leading to uterine

For peer review only - http://bmjopen.bmj.com/site/about/guidelines.xhtml

atony in the presence of tissue hypoxia[29], which can be further aggravated by uterotonics used for induction and augmentation of labour[10-12], resulting in PPH.

A prospective cohort study of about 11,000 pregnant women with moderate and severe anaemia is currently being undertaken in India through the Maternal and perinatal Health Research Collaboration, India (MaatHRI) to examine the safety of induction and augmentation of labour in this group[30]. This study will also investigate the effect of different methods of induction (mechanical using catheters and hygroscopic dilators or pharmacological using intravenous oxytocin and/ or intravaginal prostaglandins or misoprostol) and augmentation of labour (intravenous oxytocin infusion and/ or artificial rupture of membranes) on the risk of PPH and if active management of the third stage of labour modifies the risk. If an increased risk is demonstrated, there could be a potential role of the anti-fibrinolytic drug, tranexamic acid (TXA)[31], in preventing PPH in pregnant women with anaemia who undergo induction and/ or augmentation of labour, but we need further studies to find an effective dose of TXA that could prevent haemorrhage in pregnant women with moderate and severe anaemia.

Conclusion

This review gives no clear evidence regarding management of labour in pregnant women with iron deficiency anaemia. Studies suggest that pregnant women with anaemia are more likely to have sluggish uterine contractions[9 29] leading to prolonged second stage of labour and uterine atony[9 23] and consequently PPH. Despite several interventions such as routine iron supplementation during the antenatal period, there has been the best a very small decline in the prevalence of anaemia among pregnant women globally since 1995 [32]. A large number of pregnant women present with anaemia late in pregnancy or during labour. In such women appropriate peripartum care could prevent severe complications and death. The WHO provides guidance on induction and augmentation of labour and delivery[15 33], but it does not include any information about the safety of induction and/or augmentation of labour

For peer review only - http://bmjopen.bmj.com/site/about/guidelines.xhtml

in pregnant women with anaemia. At present, the decision to induce and/or augment labour in these women is not evidence-based and depends on the clinician's judgement. There is also an additional risk of unnecessary intervention or 'too much too soon' in several LMICs[34]. If induction and/ or augmentation is required, then knowing about the increased risk is important to prepare healthcare providers to proactively manage any imminent complication by augmented active management of the third stage, arranging blood/ blood products for transfusion and drugs such as tranexamic acid, which are usually not readily available in a low-resource setting. Thus, there is an urgent need to conduct further research to investigate the safety of induction and augmentation of labour in pregnant women with anaemia.

REFERENCES

1. de Benoist B, McLean E, Egli I, et al., editors. *Worldwide prevalence of anaemia 1993–2005 : WHO global database on anaemia*. Geneva: World Health Organisation, 2008.
2. Toteja GS, Singh P, Dhillon BS, et al. Prevalence of anemia among pregnant women and adolescent girls in 16 districts of India. Food and nutrition bulletin 2006;**27**(4):311-5.
3. Kavle JA, Stoltzfus RJ, Witter F, et al. Association between Anaemia during Pregnancy and Blood Loss at and after Delivery among Women with Vaginal Births in Pemba Island, Zanzibar, Tanzania. Journal of Health, Population, and Nutrition 2008;**26**(2):232-40.
4. Grewal A. Anaemia and pregnancy: Anaesthetic implications. Indian Journal of Anaesthesia 2010;**54**(5):380-86.
5. Chhabra S, Sirohi R. Trends in maternal mortality due to haemorrhage: two decades of Indian rural observations. Journal of Obstetrics & Gynecology 2004;**24**(1):40-43.
6. Rohilla M, Raveendran A, Dhaliwal L, et al. Severe anaemia in pregnancy: a tertiary hospital experience from northern India. Journal of Obstetrics & Gynaecology 2010;**30**(7):694-96.
7. Begley Cecily M, Gyte Gillian ML, Devane D, et al. Active versus expectant management for women in the third stage of labour. Cochrane Database of Systematic Reviews 2015; (3). http://onlinelibrary.wiley.com/doi/10.1002/14651858.CD007412.pub4/abstract

http://onlinelibrary.wiley.com/store/10.1002/14651858.CD007412.pub4/asset/CD007412.pdf?v=1&t=ihrpfx40&s=7958f2208cd4269e8081aed33c7a9bf798ef1221.
8. Nair M, Choudhury MK, Choudhury SS, et al. The association between maternal anaemia and pregnancy outcomes: a cohort study in Assam, India. BMJ Global Health 2016;**1**:e000026.
9. Malhotra M, Sharma JB, Batra S, et al. Maternal and perinatal outcome in varying degrees of anemia. International Journal of Gynecology & Obstetrics 2002;**79**(2):93-100.
10. Li H, Gudmundsson S, Olofsson P. Uterine artery blood flow velocity waveforms during uterine contractions. Ultrasound in obstetrics & gynecology : the official journal of the International Society of Ultrasound in Obstetrics and Gynecology 2003;**22**(6):578-85.
11. Brotanek V, Hendricks CH, Yoshida T. Changes in uterine blood flow during uterine contractions. American journal of obstetrics and gynecology 1969;**103**(8):1108-16.
12. Stanton CK, Deepak NN, Mallapur AA, et al. Direct observation of uterotonic drug use at public health facility-based deliveries in four districts in India. International journal of gynaecology and obstetrics: the official organ of the International Federation of Gynaecology and Obstetrics 2014;**127**(1):25-30.

For peer review only - http://bmjopen.bmj.com/site/about/guidelines.xhtml

13. WHO, UNICEF, UNU. Iron deficiency anaemia: assessment, prevention and control, a guide for programme managers. 2001. http://www.who.int/nutrition/publications/micronutrients/anaemia_iron_deficiency/WHO_NHD_01.3/en/index.html (accessed 11 April 2014).
14. Eke AC, Okigbo C. Mechanical methods for induction of labour: RHL commentary (last revised: 1 August 2012) Geneva: World Health Organization: The WHO Reproductive Health Library; [cited 2015 02 December]. Available from: http://apps.who.int/rhl/pregnancy_childbirth/induction/cd001233_ekea_com/en/index.html.
15. World Health Organisation. *WHO recommendations for augmentation of labour*. Geneva: World Health Organisation, 2014.
16. World Health Organisation. *WHO recommendations for the prevention and treatment of postpartum haemorrhage*. Geneva, Switzerland World Health Organisation, 2012.
17. World Health Organisation. *The WHO application of ICD-10 to deaths during pregnancy, childbirth and puerperium: ICD MM*. Geneva World Health Organisation.
18. Brown SA, Upchurch SL, Acton GJ. A framework for developing a coding scheme for meta-analysis. Western journal of nursing research 2003;**25**(2):205-22.
19. Altman DG. Measuring agreement. In: Altman DG, ed. Practical statistics for medical research. London: Chapman and Hall, 1991.
20. Tee ES, Kandiah M, Ali J, et al. Nutritional anemia in pregnancy: a study at the maternity hospital, Kuala Lumpur. Malays J Reprod Health 1984;**2**(1):32-50.
21. Tsu VD. Postpartum haemorrhage in Zimbabwe: a risk factor analysis. Br J Obstet Gynaecol 1993;**100**(4):327-33.
22. Phillip H, Fletcher H, Reid M. The impact of induced labour on postpartum blood loss. J Obstet Gynaecol 2004;**24**(1):12-5.
23. Wang X, Tan H, Zhou S, et al. [Incidence and risk factors for postpartum hemorrhage in Liuyang, China]. Zhong Nan Da Xue Xue Bao Yi Xue Ban 2014;**Yi Xue Ban = Journal of Central South University. Medical Sciences. 39**(2):151-6.
24. Donchev V. [The effects of anemia during pregnancy on the course of labor]. Akush Ginekol (Mosk) 1974;**13**(6):421-30.
25. Salwa M. [Effect of anemia in pregnancy on labor complications]. Ginekol Pol 1975;**46**(5):513-7.
26. Daraz B. [Clinical study of labor management in women with anemia]. Ginekol Pol 1978;**49**(9):829-30.
27. Sheldon WR, Blum J, Vogel JP, et al. Postpartum haemorrhage management, risks, and maternal outcomes: findings from the World Health Organization Multicountry Survey on Maternal and Newborn Health. Bjog 2014;**121 Suppl 1**:5-13.
28. Wetta LA, Szychowski JM, Seals S, et al. Risk factors for uterine atony/postpartum hemorrhage requiring treatment after vaginal delivery. Am J Obstet Gynecol 2013;**209**(1):51.e1-6.
29. Soltan MH, Ibrahim EM, Tawfek M, et al. Raised nitric oxide levels may cause atonic postpartum hemorrhage in women with anemia during pregnancy. International journal of gynaecology and obstetrics: the official organ of the International Federation of Gynaecology and Obstetrics 2012;**116**(2):143-7.
30. National Perinatal Epidemiology Unit. MaatHRI: Maternal and perinatal Health Research collaboration, India Oxford: National Perinatal Epidemiology Unit, University of Oxford; 2017 [cited 2017 14 December]. Available from: https://www.npeu.ox.ac.uk/maathri.
31. Shakur H, Roberts I, Fawole B, et al. Effect of early tranexamic acid administration on mortality, hysterectomy, and other morbidities in women with post-partum haemorrhage (WOMAN): an international, randomised, double-blind, placebo-controlled trial. The Lancet 2017;**389**(10084):2105-16.
32. Stevens GA, Finucane MM, De-Regil LM, et al. Global, regional, and national trends in haemoglobin concentration and prevalence of total and severe anaemia in children and

For peer review only - http://bmjopen.bmj.com/site/about/guidelines.xhtml

pregnant and non-pregnant women for 1995-2011: a systematic analysis of population-representative data. The Lancet Global Health 2014;**1**(1):e16-e25.
33. WHO. *WHO recommendations for induction of labour*. Geneva: WHO, 2011.
34. Miller S, Abalos E, Chamillard M, et al. Beyond too little, too late and too much, too soon: a pathway towards evidence-based, respectful maternity care worldwide. The Lancet 2016;**388**(10056):2176-92.

Supplementary file S1: Search strategies

Acknowledgement: We would like to acknowledge the help from Anna Balchan, UKOSS/BAPS-CASS Programme Administrator at the NPEU, in reading and translating the papers published in Polish.

Disclosure of interests: The authors declare that they have no competing interests.

Contribution to authorship: KB reviewed the literature, extracted information from studies and edited the paper; NR conducted the searches and edited the paper; MK was the third reviewer and edited the paper; MN developed the concept for the systematic review, reviewed the literature, extracted information from studies and wrote the first draft of the paper.

Details of ethics approval: Not applicable since this is a systematic review.

Funding: The work was funded by a Medical Research Council (MRC) Career Development Award (https://www.mrc.ac.uk/) for Manisha Nair (Grant Ref: MR/P022030/1). The funder had no role in study design, data collection, data analysis, data interpretation, or writing of the report. The corresponding author had full access to all the data in the study and had final responsibility for the decision to submit for publication.

Data sharing statement: Data sharing is not applicable to this article as no datasets were generated or analysed during the review.

Figure legend

Figure-1: PRISMA flow-chart of the selection process for the systematic review

For peer review only - http://bmjopen.bmj.com/site/about/guidelines.xhtml

Figure-1: PRISMA flow-chart of the selection process for the systematic review

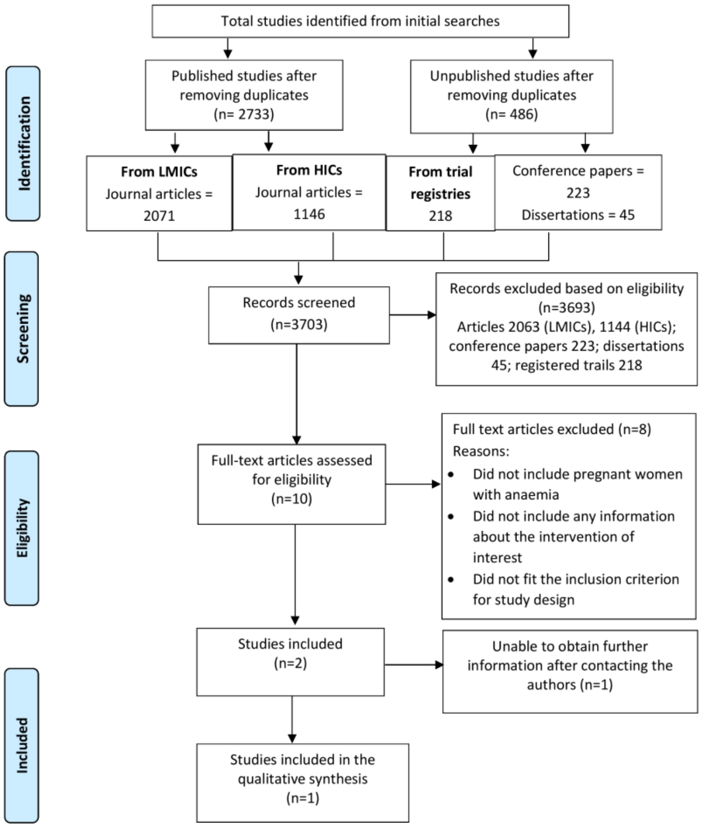

LMICs: Low-to-middle income countries; HICs: High income countries

Figure-1: PRISMA flow-chart of the selection process for the systematic review

209x297mm (300 x 300 DPI)

S1 Text

Search strategies

Medline

# ▲	Searches
1	anemia/ or anemia, hypochromic/ or anemia, iron-deficiency/
2	(anaemia or anemia).ti.
3	((iron deficien* or hypochromic or microcytic) adj5 (anaemia or anemia)).ti,ab.
4	((pregnan* or gestation* or maternal) and (anaemia or anemia)).ti,ab.
5	(Pregnancy/ or Pregnant woman/) and (anaemia or anemia).mp.
6	1 or 2 or 3 or 4 or 5
7	exp Labor, Obstetric/
8	exp Parturition/
9	exp Delivery, Obstetric/
10	(labor or labour or birth? or childbirth? or delivery or obstetric*).ti.
11	((induction? or induced or inducing) adj5 (labor or labour or birth? or childbirth? or delivery)).ti,ab.
12	(augment* adj5 (labor or labour or birth? or childbirth? or delivery)).ti,ab.
13	(manage* adj5 (labor or labour or birth? or childbirth? or delivery)).ti,ab.
14	(initiat* adj5 (labor or labour or birth? or childbirth? or delivery)).ti,ab.
15	Oxytocin/
16	exp Prostaglandins E, Synthetic/
17	(uterotonic? or oxytocin or prostaglandin? or misoprostol).ti,ab.
18	7 or 8 or 9 or 10 or 11 or 12 or 13 or 14 or 15 or 16 or 17
19	Developing Countries/
20	(Africa or Caribbean or West Indies or South America or Latin America or Central America).hw,kf,ti,ab,cp.

For peer review only

21	(Afghanistan or Albania or Algeria or Angola or American Samoa or Armenia or Armenian or Azerbaijan or Bangladesh or Benin or Byelarus or Byelorussian or Belarus or Belorussian or Belorussia or Belize or Bhutan or Bolivia or Bosnia or Herzegovina or Hercegovina or Botswana or Brazil or Brasil or Bulgaria or Burkina Faso or Burkina Fasso or Upper Volta or Burundi or Urundi or Cambodia or Khmer Republic or Kampuchea or Cameroon or Cameroons or Cameron or Camerons or Cape Verde or Central African Republic or Chad or China or Colombia or Comoros or Comoro Islands or Comores or Mayotte or Congo or Zaire or Costa Rica or Cote d'Ivoire or Ivory Coast or Cuba or Djibouti or French Somaliland or Dominica or Dominican Republic or East Timor or East Timur or Timor Leste or Ecuador or Egypt or United Arab Republic or El Salvador or Eritrea or Ethiopia or Fiji or Gabon or Gabonese Republic or Gambia or Gaza or Georgia Republic or Georgian Republic or Ghana or Gold Coast or Grenada or Guatemala or Guinea or Guinea-Bisau or Guam or Guiana or Guyana or Haiti or Honduras or India or Maldives or Indonesia or Iran or Iraq or Jamaica or Jordan or Kazakhstan or Kazakh or Kenya or Kiribati or Korea or Kosovo or Kyrgyzstan or Kirghizia or Kyrgyz Republic or Kirghiz or Kirgizstan or Lao PDR or Laos or Lebanon or Lesotho or Basutoland or Liberia or Libya or Macedonia or Madagascar or Malagasy Republic or Malaysia or Malaya or Malay or Sabah or Sarawak or Malawi or Nyasaland or Mali or Marshall Islands or Mauritania or Mauritius or Agalega Islands or Mexico or Micronesia or Middle East or Moldova or Moldovia or Moldovian or Mongolia or Montenegro or Morocco or Ifni or Mozambique or Myanmar or Myanma or Burma or Namibia or Nepal or Netherlands Antilles or Nicaragua or Niger or Nigeria or Pakistan or Palau or Palestine or Panama or Papua New Guinea or Paraguay or Peru or Philippines or Philipines or Phillipines or Phillippines or Romania or Rumania or Roumania or Rwanda or Ruanda or Saint Lucia or St Lucia or Saint Vincent or St Vincent or Grenadines or Samoa or Samoan Islands or Navigator Island or Navigator Islands or Sao Tome or Senegal or Serbia or Sierra Leone or Sri Lanka or Ceylon or Solomon Islands or Somalia or Sudan or Suriname or Surinam or Swaziland or Syria or Principe or South Sudan or Tajikistan or Tadzhikistan or Tadjikistan or Tadzhik or Tanzania or Thailand or Timor-Leste or Togo or Togolese Republic or Tonga or Tunisia or Turkey or Turkmenistan or Turkmen or Tuvalu or Uganda or Ukraine or Uzbekistan or Uzbek or Vanuatu or New Hebrides or Vietnam or Viet Nam or West Bank or Yemen or Zambia or Zimbabwe or Rhodesia).hw,kf,ti,ab,cp.
22	((developing or less* developed or under developed or underdeveloped or middle income or low* income or underserved or under served or deprived or poor*) adj (countr* or nation? or state? or population? or world)).ti,ab.
23	((developing or less* developed or under developed or underdeveloped or middle income or low* income) adj (economy or economies)).ti,ab.
24	(low* adj (gdp or gnp or gross domestic or gross national)).ti,ab.
25	(low adj3 middle adj3 countr*).ti,ab.
26	(lmic or lmics or third world or lami countr*).ti,ab.
27	transitional countr*.ti,ab.
28	19 or 20 or 21 or 22 or 23 or 24 or 25 or 26 or 27
29	6 and 18 and 28

For peer review only

Embase

# ▲	Searches
1	*anemia/ or iron deficiency anemia/ or microcytic anemia/
2	(anaemia or anemia).ti.
3	((iron deficien* or hypochromic or microcytic) adj5 (anaemia or anemia)).ti,ab.
4	((pregnan* or gestation* or maternal) and (anaemia or anemia)).ti,ab.
5	(Pregnancy/ or Pregnant woman/) and (anaemia or anemia).mp.
6	1 or 2 or 3 or 4 or 5
7	exp labor/
8	childbirth/
9	exp delivery/
10	(labor or labour or birth? or childbirth? or delivery or obstetric*).ti.
11	((induction? or induced or inducing) adj5 (labor or labour or birth? or childbirth? or delivery)).ti,ab.
12	(augment* adj5 (labor or labour or birth? or childbirth? or delivery)).ti,ab.
13	(manage* adj5 (labor or labour or birth? or childbirth? or delivery)).ti,ab.
14	(initiat* adj5 (labor or labour or birth? or childbirth? or delivery)).ti,ab.
15	exp uterotonic agent/
16	(uterotonic? or oxytocin or prostaglandin? or misoprostol).ti,ab.
17	7 or 8 or 9 or 10 or 11 or 12 or 13 or 14 or 15 or 16
18	Developing Country.sh.
19	(Africa or Caribbean or West Indies or South America or Latin America or Central America).hw,ti,ab,cp.

For peer review only

20	(Afghanistan or Albania or Algeria or Angola or American Samoa or Armenia or Armenian or Azerbaijan or Bangladesh or Benin or Byelarus or Byelorussian or Belarus or Belorussian or Belorussia or Belize or Bhutan or Bolivia or Bosnia or Herzegovina or Hercegovina or Botswana or Brazil or Brasil or Bulgaria or Burkina Faso or Burkina Fasso or Upper Volta or Burundi or Urundi or Cambodia or Khmer Republic or Kampuchea or Cameroon or Cameroons or Cameron or Camerons or Cape Verde or Central African Republic or Chad or China or Colombia or Comoros or Comoro Islands or Comores or Mayotte or Congo or Zaire or Costa Rica or Cote d'Ivoire or Ivory Coast or Cuba or Djibouti or French Somaliland or Dominica or Dominican Republic or East Timor or East Timur or Timor Leste or Ecuador or Egypt or United Arab Republic or El Salvador or Eritrea or Ethiopia or Fiji or Gabon or Gabonese Republic or Gambia or Gaza or Georgia Republic or Georgian Republic or Ghana or Gold Coast or Grenada or Guatemala or Guinea or Guinea-Bisau or Guam or Guiana or Guyana or Haiti or Honduras or India or Maldives or Indonesia or Iran or Iraq or Jamaica or Jordan or Kazakhstan or Kazakh or Kenya or Kiribati or Korea or Kosovo or Kyrgyzstan or Kirghizia or Kyrgyz Republic or Kirghiz or Kirgizstan or Lao PDR or Laos or Lebanon or Lesotho or Basutoland or Liberia or Libya or Macedonia or Madagascar or Malagasy Republic or Malaysia or Malaya or Malay or Sabah or Sarawak or Malawi or Nyasaland or Mali or Marshall Islands or Mauritania or Mauritius or Agalega Islands or Mexico or Micronesia or Middle East or Moldova or Moldovia or Moldovian or Mongolia or Montenegro or Morocco or Ifni or Mozambique or Myanmar or Myanma or Burma or Namibia or Nepal or Netherlands Antilles or Nicaragua or Niger or Nigeria or Pakistan or Palau or Palestine or Panama or Papua New Guinea or Paraguay or Peru or Philippines or Philipines or Phillipines or Phillippines or Romania or Rumania or Roumania or Rwanda or Ruanda or Saint Lucia or St Lucia or Saint Vincent or St Vincent or Grenadines or Samoa or Samoan Islands or Navigator Island or Navigator Islands or Sao Tome or Senegal or Serbia or Sierra Leone or Sri Lanka or Ceylon or Solomon Islands or Somalia or Sudan or Suriname or Surinam or Swaziland or Syria or Principe or South Sudan or Tajikistan or Tadzhikistan or Tadjikistan or Tadzhik or Tanzania or Thailand or Timor-Leste or Togo or Togolese Republic or Tonga or Tunisia or Turkey or Turkmenistan or Turkmen or Tuvalu or Uganda or Ukraine or Uzbekistan or Uzbek or Vanuatu or New Hebrides or Vietnam or Viet Nam or West Bank or Yemen or Zambia or Zimbabwe or Rhodesia).hw,ti,ab,cp.
21	((developing or less* developed or under developed or underdeveloped or middle income or low* income or underserved or under served or deprived or poor*) adj (countr* or nation? or state? or population? or world)).ti,ab.
22	((developing or less* developed or under developed or underdeveloped or middle income or low* income) adj (economy or economies)).ti,ab.
23	(low* adj (gdp or gnp or gross domestic or gross national)).ti,ab.
24	(low adj3 middle adj3 countr*).ti,ab.
25	(lmic or lmics or third world or lami countr*).ti,ab.
26	transitional countr*.ti,ab.
27	18 or 19 or 20 or 21 or 22 or 23 or 24 or 25 or 26
28	6 and 17 and 27

For peer review only - http://bmjopen.bmj.com/site/about/guidelines.xhtml

Global Health

# ▲	Searches
1	anaemia/ or hypochromic anaemia/ or iron deficiency anaemia/ or microcytic anaemia/
2	(anaemia or anemia).ti.
3	((iron deficien* or hypochrmomic or microcytic) adj5 (anaemia or anemia)).ti,ab.
4	((pregnan* or gestation* or maternal) and (anaemia or anemia)).ti,ab.
5	1 or 2 or 3 or 4
6	birth/ or childbirth/ or parturition/
7	(labor or labour or birth? or childbirth? or delivery or obstetric*).ti.
8	((induction? or induced or inducing) adj5 (labor or labour or birth? or childbirth? or delivery)).ti,ab.
9	(augment* adj5 (labor or labour or birth? or childbirth? or delivery)).ti,ab.
10	(manage* adj5 (labor or labour or birth? or childbirth? or delivery)).ti,ab.
11	oxytocin/
12	exp synthetic prostaglandins/
13	(uterotonic? or oxytocin or prostaglandin? or misoprostol).ti,ab.
14	6 or 7 or 8 or 9 or 10 or 11 or 12 or 13
15	exp developing countries/ or exp africa/
16	(Africa or Caribbean or West Indies or South America or Latin America or Central America).hw,ti,ab,cp.
17	(Afghanistan or Albania or Algeria or Angola or American Samoa or Armenia or Armenian or Azerbaijan or Bangladesh or Benin or Byelarus or Byelorussian or Belarus or Belorussian or Belorussia or Belize or Bhutan or Bolivia or Bosnia or Herzegovina or Hercegovina or Botswana or Brazil or Brasil or Bulgaria or Burkina Faso or Burkina Fasso or Upper Volta or Burundi or Urundi or Cambodia or Khmer Republic or Kampuchea or Cameroon or Cameroons or Cameron or Camerons or Cape Verde or Central African Republic or Chad or China or Colombia or Comoros or Comoro Islands or Comores or Mayotte or Congo or Zaire or Costa Rica or Cote d'Ivoire or Ivory Coast or Cuba or Djibouti or French Somaliland or Dominica or Dominican Republic or East Timor or East Timur or Timor Leste or Ecuador or Egypt or United Arab Republic or El Salvador or Eritrea or Ethiopia or Fiji or Gabon or Gabonese Republic or Gambia or Gaza or Georgia Republic or Georgian Republic or Ghana or Gold Coast or Grenada or Guatemala or Guinea or Guinea-Bisau or Guam or Guiana or Guyana or Haiti or Honduras or India or Maldives or Indonesia or Iran or Iraq or Jamaica or Jordan or Kazakhstan or Kazakh or Kenya or Kiribati or Korea or Kosovo or Kyrgyzstan or Kirghizia or Kyrgyz Republic or Kirghiz or Kirgizstan or Lao PDR or Laos or Lebanon or Lesotho or Basutoland or Liberia or Libya or Macedonia or Madagascar or Malagasy Republic or Malaysia or Malaya or Malay or Sabah or Sarawak or Malawi or Nyasaland or Mali or Marshall Islands or Mauritania or Mauritius or Agalega Islands or Mexico or Micronesia or Middle East or Moldova or Moldovia or Moldovian or Mongolia or Montenegro or Morocco or Ifni or Mozambique or Myanmar or Myanma or Burma or Namibia or Nepal or Netherlands Antilles or Nicaragua or Niger or Nigeria or Pakistan or Palau or Palestine or Panama or Papua New Guinea or Paraguay or Peru or Philippines or Philipines or Phillipines or Phillippines or Romania or Rumania or Roumania or Rwanda or Ruanda or Saint Lucia or St Lucia or Saint Vincent or St Vincent or Grenadines or Samoa or Samoan Islands or Navigator Island or Navigator Islands or Sao Tome or Senegal or Serbia or Sierra Leone or Sri Lanka or Ceylon or Solomon Islands or Somalia or Sudan or Suriname or Surinam or Swaziland or Syria or Principe or South Sudan or Tajikistan or Tadzhikistan or Tadjikistan or Tadzhik or Tanzania or Thailand or Timor-Leste or Togo or

For peer review only - http://bmjopen.bmj.com/site/about/guidelines.xhtml

	Togolese Republic or Tonga or Tunisia or Turkey or Turkmenistan or Turkmen or Tuvalu or Uganda or Ukraine or Uzbekistan or Uzbek or Vanuatu or New Hebrides or Vietnam or Viet Nam or West Bank or Yemen or Zambia or Zimbabwe or Rhodesia).hw,ti,ab,cp.
18	((developing or less* developed or under developed or underdeveloped or middle income or low* income or underserved or underserved or deprived or poor*) adj (countr* or nation? or state? or population? or world)).ti,ab.
19	((developing or less* developed or under developed or underdeveloped or middle income or low* income) adj (economy or economies)).ti,ab.
20	(low* adj (gdp or gnp or gross domestic or gross national)).ti,ab.
21	(low adj3 middle adj3 countr*).ti,ab.
22	(lmic or lmics or third world or lami countr*).ti,ab.
23	transitional countr*.ti,ab.
24	15 or 16 or 17 or 18 or 19 or 20 or 21 or 22 or 23
25	5 and 14 and 24

For peer review only

For peer review only - http://bmjopen.bmj.com/site/about/guidelines.xhtml

CINAHL

#	Query
1	(MH "Anemia") OR (MH "Anemia, Hypochromic") OR (MH "Anemia, Iron Deficiency")
2	TI (anaemia or anemia) OR AB (((iron deficien* or hypochromic or microcytic) N5 (anaemia or anemia))) OR AB (((pregnan* or gestation* or maternal) and (anaemia or anemia)))
3	((MH "Pregnancy") OR (MH "Expectant Mothers")) AND (anaemia or anemia)
4	S1 OR S2 OR S3
5	(MH "Childbirth+") OR (MH "Labor+")
6	(MH "Delivery, Obstetric+")
7	TI (labor or labour or birth? or childbirth? or delivery or obstetric*) OR AB (((induction? or induced or inducing) N5 (labor or labour or birth? or childbirth? or delivery))) OR AB ((augment* N5 (labor or labour or birth? or childbirth? or delivery))) OR AB ((manage* N5 (labor or labour or birth? or childbirth? or delivery))) OR AB ((initiat* N5 (labor or labour or birth? or childbirth? or delivery)))
8	(MH "Oxytocics+")
9	(MH "Prostaglandins, Synthetic+")
10	TI (uterotonic? or oxytocin or prostaglandin? or misoprostol) OR AB (uterotonic? or oxytocin or prostaglandin? or misoprostol)
11	S5 OR S6 OR S7 OR S8 OR S9 OR S10
12	(MH "Developing Countries")
13	TI (Afghanistan or Albania or Algeria or Angola or American Samoa or Armenia or Armenian or Azerbaijan or Bangladesh or Benin or Byelarus or Byelorussian or Belarus or Belorussian or Belorussia or Belize or Bhutan or Bolivia or Bosnia or Herzegovina or Hercegovina or Botswana or Brazil or Brasil or Bulgaria or Burkina Faso or Burkina Fasso or Upper Volta or Burundi or Urundi or Cambodia or Khmer Republic or Kampuchea or Cameroon or Cameroons or Cameron or Camerons or Cape Verde or Central African Republic or Chad or China or Colombia or Comoros or Comoro Islands or Comores or Mayotte or Congo or Zaire or Costa Rica or Cote d'Ivoire or Ivory Coast or Cuba or Djibouti or French Somaliland or Dominica or Dominican Republic or East Timor or East Timur or Timor Leste or Ecuador or Egypt or United Arab Republic or El Salvador or Eritrea or Ethiopia or Fiji or Gabon or Gabonese Republic or Gambia or Gaza or Georgia Republic or Georgian Republic or Ghana or Gold Coast or Grenada or Guatemala or Guinea or Guinea-Bisau or Guam or Guiana or Guyana or Haiti or Honduras or India or Maldives or Indonesia or Iran or Iraq or Jamaica or Jordan or Kazakhstan or Kazakh or Kenya or Kiribati or Korea or Kosovo or Kyrgyzstan or Kirghizia or Kyrgyz Republic or Kirghiz or Kirgizstan or Lao PDR or Laos or Lebanon or Lesotho or Basutoland or Liberia or Libya or Macedonia or Madagascar or Malagasy Republic or Malaysia or Malaya or Malay or Sabah or Sarawak or Malawi or Nyasaland or Mali or Marshall Islands or Mauritania or Mauritius or Agalega Islands or Mexico or Micronesia or Middle East or Moldova or Moldovia or Moldovian or Mongolia or Montenegro or Morocco or Ifni or Mozambique or Myanmar or Myanma or Burma or Namibia or Nepal or Netherlands Antilles or Nicaragua or Niger or Nigeria or Pakistan or Palau or Palestine or Panama or Papua New Guinea or Paraguay or Peru or Philippines or Philipines or Phillipines or Phillippines or Romania or Rumania or Roumania or Rwanda or Ruanda or Saint Lucia or St Lucia or Saint Vincent or St Vincent or Grenadines or Samoa or Samoan Islands or Navigator Island or Navigator Islands or Sao Tome or Senegal or Serbia or Sierra Leone or Sri Lanka or Ceylon or Solomon Islands or Somalia or Sudan or Suriname or Surinam or Swaziland or Syria or Principe or South

For peer review only - http://bmjopen.bmj.com/site/about/guidelines.xhtml

	Sudan or Tajikistan or Tadzhikistan or Tadjikistan or Tadzhik or Tanzania or Thailand or Timor-Leste or Togo or Togolese Republic or Tonga or Tunisia or Turkey or Turkmenistan or Turkmen or Tuvalu or Uganda or Ukraine or Uzbekistan or Uzbek or Vanuatu or New Hebrides or Vietnam or Viet Nam or West Bank or Yemen or Zambia or Zimbabwe or Rhodesia) OR AB (Afghanistan or Albania or Algeria or Angola or American Samoa or Armenia or Armenian or Azerbaijan or Bangladesh or Benin or Byelarus or Byelorussian or Belarus or Belorussian or Belorussia or Belize or Bhutan or Bolivia or Bosnia or Herzegovina or Hercegovina or Botswana or Brazil or Brasil or Bulgaria or Burkina Faso or Burkina Fasso or Upper Volta or Burundi or Urundi or Cambodia or Khmer Republic or Kampuchea or Cameroon or Cameroons or Cameron or Camerons or Cape Verde or Central African Republic or Chad or China or Colombia or Comoros or Comoro Islands or Comores or Mayotte or Congo or Zaire or Costa Rica or Cote d'Ivoire or Ivory Coast or Cuba or Djibouti or French Somaliland or Dominica or Dominican Republic or East Timor or East Timur or Timor Leste or Ecuador or Egypt or United Arab Republic or El Salvador or Eritrea or Ethiopia or Fiji or Gabon or Gabonese Republic or Gambia or Gaza or Georgia Republic or Georgian Republic or Ghana or Gold Coast or Grenada or Guatemala or Guinea or Guinea-Bisau or Guam or Guiana or Guyana or Haiti or Honduras or India or Maldives or Indonesia or Iran or Iraq or Jamaica or Jordan or Kazakhstan or Kazakh or Kenya or Kiribati or Korea or Kosovo or Kyrgyzstan or Kirghizia or Kyrgyz Republic or Kirghiz or Kirgizstan or Lao PDR or Laos or Lebanon or Lesotho or Basutoland or Liberia or Libya or Macedonia or Madagascar or Malagasy Republic or Malaysia or Malaya or Malay or Sabah or Sarawak or Malawi or Nyasaland or Mali or Marshall Islands or Mauritania or Mauritius or Agalega Islands or Mexico or Micronesia or Middle East or Moldova or Moldovia or Moldovian or Mongolia or Montenegro or Morocco or Ifni or Mozambique or Myanmar or Myanma or Burma or Namibia or Nepal or Netherlands Antilles or Nicaragua or Niger or Nigeria or Pakistan or Palau or Palestine or Panama or Papua New Guinea or Paraguay or Peru or Philippines or Philipines or Phillipines or Phillippines or Romania or Rumania or Roumania or Rwanda or Ruanda or Saint Lucia or St Lucia or Saint Vincent or St Vincent or Grenadines or Samoa or Samoan Islands or Navigator Island or Navigator Islands or Sao Tome or Senegal or Serbia or Sierra Leone or Sri Lanka or Ceylon or Solomon Islands or Somalia or Sudan or Suriname or Surinam or Swaziland or Syria or Principe or South Sudan or Tajikistan or Tadzhikistan or Tadjikistan or Tadzhik or Tanzania or Thailand or Timor-Leste or Togo or Togolese Republic or Tonga or Tunisia or Turkey or Turkmenistan or Turkmen or Tuvalu or Uganda or Ukraine or Uzbekistan or Uzbek or Vanuatu or New Hebrides or Vietnam or Viet Nam or West Bank or Yemen or Zambia or Zimbabwe or Rhodesia) OR MW (Afghanistan or Albania or Algeria or Angola or American Samoa or Armenia or Armenian or Azerbaijan or Bangladesh or Benin or Byelarus or Byelorussian or Belarus or Belorussian or Belorussia or Belize or Bhutan or Bolivia or Bosnia or Herzegovina or Hercegovina or Botswana or Brazil or Brasil or Bulgaria or Burkina Faso or Burkina Fasso or Upper Volta or Burundi or Urundi or Cambodia or Khmer Republic or Kampuchea or Cameroon or Cameroons or Cameron or Camerons or Cape Verde or Central African Republic or Chad or China or Colombia or Comoros or Comoro Islands or Comores or Mayotte or Congo or Zaire or Costa Rica or Cote d'Ivoire or Ivory Coast or Cuba or Djibouti or French Somaliland or Dominica or Dominican Republic or East Timor or East Timur or Timor Leste or Ecuador or Egypt or United Arab Republic or El Salvador or Eritrea or Ethiopia or Fiji or Gabon or Gabonese Republic or Gambia or Gaza or Georgia Republic or Georgian Republic or Ghana or Gold Coast or Grenada or Guatemala or Guinea or Guinea-Bisau or Guam or Guiana or Guyana or Haiti or Honduras or India or Maldives or Indonesia or Iran or Iraq or Jamaica or Jordan or Kazakhstan or Kazakh or Kenya or Kiribati or Korea or Kosovo or Kyrgyzstan or Kirghizia or Kyrgyz Republic or Kirghiz or Kirgizstan or Lao PDR or Laos or Lebanon or Lesotho or Basutoland

For peer review only - http://bmjopen.bmj.com/site/about/guidelines.xhtml

	or Liberia or Libya or Macedonia or Madagascar or Malagasy Republic or Malaysia or Malaya or Malay or Sabah or Sarawak or Malawi or Nyasaland or Mali or Marshall Islands or Mauritania or Mauritius or Agalega Islands or Mexico or Micronesia or Middle East or Moldova or Moldovia or Moldovian or Mongolia or Montenegro or Morocco or Ifni or Mozambique or Myanmar or Myanma or Burma or Namibia or Nepal or Netherlands Antilles or Nicaragua or Niger or Nigeria or Pakistan or Palau or Palestine or Panama or Papua New Guinea or Paraguay or Peru or Philippines or Philipines or Phillipines or Phillippines or Romania or Rumania or Roumania or Rwanda or Ruanda or Saint Lucia or St Lucia or Saint Vincent or St Vincent or Grenadines or Samoa or Samoan Islands or Navigator Island or Navigator Islands or Sao Tome or Senegal or Serbia or Sierra Leone or Sri Lanka or Ceylon or Solomon Islands or Somalia or Sudan or Suriname or Surinam or Swaziland or Syria or Principe or South Sudan or Tajikistan or Tadzhikistan or Tadjikistan or Tadzhik or Tanzania or Thailand or Timor-Leste or Togo or Togolese Republic or Tonga or Tunisia or Turkey or Turkmenistan or Turkmen or Tuvalu or Uganda or Ukraine or Uzbekistan or Uzbek or Vanuatu or New Hebrides or Vietnam or Viet Nam or West Bank or Yemen or Zambia or Zimbabwe or Rhodesia) OR AF (Afghanistan or Albania or Algeria or Angola or American Samoa or Armenia or Armenian or Azerbaijan or Bangladesh or Benin or Byelarus or Byelorussian or Belarus or Belorussian or Belorussia or Belize or Bhutan or Bolivia or Bosnia or Herzegovina or Hercegovina or Botswana or Brazil or Brasil or Bulgaria or Burkina Faso or Burkina Fasso or Upper Volta or Burundi or Urundi or Cambodia or Khmer Republic or Kampuchea or Cameroon or Cameroons or Cameron or Camerons or Cape Verde or Central African Republic or Chad or China or Colombia or Comoros or Comoro Islands or Comores or Mayotte or Congo or Zaire or Costa Rica or Cote d'Ivoire or Ivory Coast or Cuba or Djibouti or French Somaliland or Dominica or Dominican Republic or East Timor or East Timur or Timor Leste or Ecuador or Egypt or United Arab Republic or El Salvador or Eritrea or Ethiopia or Fiji or Gabon or Gabonese Republic or Gambia or Gaza or Georgia Republic or Georgian Republic or Ghana or Gold Coast or Grenada or Guatemala or Guinea or Guinea-Bisau or Guam or Guiana or Guyana or Haiti or Honduras or India or Maldives or Indonesia or Iran or Iraq or Jamaica or Jordan or Kazakhstan or Kazakh or Kenya or Kiribati or Korea or Kosovo or Kyrgyzstan or Kirghizia or Kyrgyz Republic or Kirghiz or Kirgizstan or Lao PDR or Laos or Lebanon or Lesotho or Basutoland or Liberia or Libya or Macedonia or Madagascar or Malagasy Republic or Malaysia or Malaya or Malay or Sabah or Sarawak or Malawi or Nyasaland or Mali or Marshall Islands or Mauritania or Mauritius or Agalega Islands or Mexico or Micronesia or Middle East or Moldova or Moldovia or Moldovian or Mongolia or Montenegro or Morocco or Ifni or Mozambique or Myanmar or Myanma or Burma or Namibia or Nepal or Netherlands Antilles or Nicaragua or Niger or Nigeria or Pakistan or Palau or Palestine or Panama or Papua New Guinea or Paraguay or Peru or Philippines or Philipines or Phillipines or Phillippines or Romania or Rumania or Roumania or Rwanda or Ruanda or Saint Lucia or St Lucia or Saint Vincent or St Vincent or Grenadines or Samoa or Samoan Islands or Navigator Island or Navigator Islands or Sao Tome or Senegal or Serbia or Sierra Leone or Sri Lanka or Ceylon or Solomon Islands or Somalia or Sudan or Suriname or Surinam or Swaziland or Syria or Principe or South Sudan or Tajikistan or Tadzhikistan or Tadjikistan or Tadzhik or Tanzania or Thailand or Timor-Leste or Togo or Togolese Republic or Tonga or Tunisia or Turkey or Turkmenistan or Turkmen or Tuvalu or Uganda or Ukraine or Uzbekistan or Uzbek or Vanuatu or New Hebrides or Vietnam or Viet Nam or West Bank or Yemen or Zambia or Zimbabwe or Rhodesia)

For peer review only - http://bmjopen.bmj.com/site/about/guidelines.xhtml

14	(((developing or less* developed or under developed or underdeveloped or middle income or low* income or underserved or under served or deprived or poor*) N1 (countr* or nation? or state? or population? or world))) OR (((developing or less* developed or under developed or underdeveloped or middle income or low* income) N1 (economy or economies))) OR ((low* N1 (gdp or gnp or gross domestic or gross national))) OR (low N3 middle N3 countr*) OR (lmic or lmics or third world or lami countr*) OR transitional countr*
15	S12 OR S13 OR S14
16	S4 AND S11 AND S15

Cochrane Central Register of Controlled Trials

ID	Search
#1	MeSH descriptor: [Anemia, Iron-Deficiency] explode all trees
#2	MeSH descriptor: [Anemia] this term only
#3	MeSH descriptor: [Anemia, Hypochromic] this term only
#4	anaemia or anemia:ti (Word variations have been searched)
#5	((iron deficien* or hypochromic or microcytic) near (anaemia or anemia)):ti,ab,kw (Word variations have been searched)
#6	((pregnan* or gestation* or maternal) and (anaemia or anemia)):ti,ab,kw (Word variations have been searched)
#7	#1 or #2 or #3 or #4 or #5 or #6
#8	MeSH descriptor: [Labor, Obstetric] explode all trees
#9	MeSH descriptor: [Parturition] explode all trees
#10	MeSH descriptor: [Delivery, Obstetric] explode all trees
#11	labor or labour or birth? or childbirth? or delivery or obstetric*:ti (Word variations have been searched)
#12	((induction? or induced or inducing) near (labor or labour or birth? or childbirth? or delivery)):ti,ab,kw (Word variations have been searched)
#13	(augment* near (labor or labour or birth? or childbirth? or delivery)):ti,ab,kw (Word variations have been searched)
#14	(manage* near (labor or labour or birth? or childbirth? or delivery)):ti,ab,kw (Word variations have been searched)
#15	(initiat* near (labor or labour or birth? or childbirth? or delivery)):ti,ab,kw (Word variations have been searched)
#16	MeSH descriptor: [Oxytocin] explode all trees
#17	MeSH descriptor: [Prostaglandins E, Synthetic] explode all trees
#18	(uterotonic? or oxytocin or prostaglandin? or misoprostol):ti,ab,kw (Word variations have been searched)
#19	#8 or #9 or #10 or #11 or #12 or #13 or #14 or #15 or #16 or #17 or #18
#20	#7 and #19

For peer review only

For peer review only - http://bmjopen.bmj.com/site/about/guidelines.xhtml

Global Health Library

(anaemia OR anemia) AND (labour OR labor OR birth OR childbirth OR delivery OR obstetrics OR obstetric OR uterotonic OR uterotonics OR oxytocin OR prostaglandin OR prostaglandins OR misoprostol)
(iron deficiency anaemia OR iron deficiency anemia) AND (labour OR labor OR birth OR childbirth OR delivery OR obstetrics OR obstetric OR uterotonic OR uterotonics OR oxytocin OR prostaglandin OR prostaglandins OR misoprostol)
(hypochromic anaemia OR hypochromic anemia) AND (labour OR labor OR birth OR childbirth OR delivery OR obstetrics OR obstetric OR uterotonic OR uterotonics OR oxytocin OR prostaglandin OR prostaglandins OR misoprostol)
(microcytic anaemia OR microcytic anemia) AND (labour OR labor OR birth OR childbirth OR delivery OR obstetrics OR obstetric OR uterotonic OR uterotonics OR oxytocin OR prostaglandin OR prostaglandins OR misoprostol)

Dissertations & Theses (Global full text plus UK and Ireland abstracts)

Set	Search
S8	S1 AND S6
S6	ti(pregnancy OR pregnant)
S5	S1 AND S4
S4	S2 OR S3
S3	ti(uterotonic? or oxytocin or prostaglandin? or misoprostol)
S2	ti(labor or labour or birth? or childbirth? or delivery or obstetric*)
S1	ti(anaemia OR anemia)

Trials registers: Clinical trials.gov (https://clinicaltrials.gov/) and WHO ICTRP (http://apps.who.int/trialsearch/)

**Clinicaltrials.gov**
Condition=anaemia OR anemia AND Intervention=labour OR labor OR birth OR childbirth OR delivery OR obstetric OR obstetrics OR oxytocin OR prostaglandin OR prostaglandins OR misoprostol
Condition=anaemia OR anemia AND Title=labour OR labor OR birth OR childbirth OR delivery OR obstetric OR obstetrics OR oxytocin OR prostaglandin OR prostaglandins OR misoprostol
Condition=anaemia OR anemia AND Title=labour OR labor OR birth OR childbirth OR delivery OR obstetric OR obstetrics OR oxytocin OR prostaglandin OR prostaglandins OR misoprostol

**WHO Search:**
Condition=anaemia OR anemia AND Intervention=labour OR labor OR birth OR childbirth OR delivery OR obstetric OR obstetrics OR oxytocin OR prostaglandin OR prostaglandins OR misoprostol
Title=(anaemia OR anemia) AND Intervention=(labour OR labor OR birth OR childbirth OR delivery OR obstetric OR obstetrics OR oxytocin OR prostaglandin OR prostaglandins OR misoprostol)
Condition=anaemia OR anemia AND Title=labour OR labor OR birth OR childbirth OR delivery OR obstetric OR obstetrics OR oxytocin OR prostaglandin OR prostaglandins OR misoprostol

For peer review only - http://bmjopen.bmj.com/site/about/guidelines.xhtml

PRISMA 2009 Checklist

Section/topic	#	Checklist item	Reported on page #
**TITLE**			
Title	1	Identify the report as a systematic review, meta-analysis, or both.	1
**ABSTRACT**			
Structured summary	2	Provide a structured summary including, as applicable: background; objectives; data sources; study eligibility criteria, participants, and interventions; study appraisal and synthesis methods; results; limitations; conclusions and implications of key findings; systematic review registration number.	2
**INTRODUCTION**			
Rationale	3	Describe the rationale for the review in the context of what is already known.	4
Objectives	4	Provide an explicit statement of questions being addressed with reference to participants, interventions, comparisons, outcomes, and study design (PICOS).	4
**METHODS**			
Protocol and registration	5	Indicate if a review protocol exists, if and where it can be accessed (e.g., Web address), and, if available, provide registration information including registration number.	5
Eligibility criteria	6	Specify study characteristics (e.g., PICOS, length of follow-up) and report characteristics (e.g., years considered, language, publication status) used as criteria for eligibility, giving rationale.	5
Information sources	7	Describe all information sources (e.g., databases with dates of coverage, contact with study authors to identify additional studies) in the search and date last searched.	5
Search	8	Present full electronic search strategy for at least one database, including any limits used, such that it could be repeated.	6 and S1
Study selection	9	State the process for selecting studies (i.e., screening, eligibility, included in systematic review, and, if applicable, included in the meta-analysis).	5, 6, 7
Data collection process	10	Describe method of data extraction from reports (e.g., piloted forms, independently, in duplicate) and any processes for obtaining and confirming data from investigators.	7, 8
Data items	11	List and define all variables for which data were sought (e.g., PICOS, funding sources) and any assumptions and simplifications made.	7, 8
Risk of bias in individual studies	12	Describe methods used for assessing risk of bias of individual studies (including specification of whether this was done at the study or outcome level), and how this information is to be used in any data synthesis.	8
Summary measures	13	State the principal summary measures (e.g., risk ratio, difference in means).	8
Synthesis of results	14	Describe the methods of handling data and combining results of studies, if done, including measures of consistency (e.g., I^2) for each meta-analysis.	8

For peer review only - http://bmjopen.bmj.com/site/about/guidelines.xhtml

PRISMA 2009 Checklist

Section/topic	#	Checklist item	Reported on page #
Risk of bias across studies	15	Specify any assessment of risk of bias that may affect the cumulative evidence (e.g., publication bias, selective reporting within studies).	8
Additional analyses	16	Describe methods of additional analyses (e.g., sensitivity or subgroup analyses, meta-regression), if done, indicating which were pre-specified.	8
**RESULTS**			
Study selection	17	Give numbers of studies screened, assessed for eligibility, and included in the review, with reasons for exclusions at each stage, ideally with a flow diagram.	8, 9
Study characteristics	18	For each study, present characteristics for which data were extracted (e.g., study size, PICOS, follow-up period) and provide the citations.	9
Risk of bias within studies	19	Present data on risk of bias of each study and, if available, any outcome level assessment (see item 12).	9
Results of individual studies	20	For all outcomes considered (benefits or harms), present, for each study: (a) simple summary data for each intervention group (b) effect estimates and confidence intervals, ideally with a forest plot.	9 and Table-1 (10)
Synthesis of results	21	Present results of each meta-analysis done, including confidence intervals and measures of consistency.	9
Risk of bias across studies	22	Present results of any assessment of risk of bias across studies (see Item 15).	NA
Additional analysis	23	Give results of additional analyses, if done (e.g., sensitivity or subgroup analyses, meta-regression [see Item 16]).	NA
**DISCUSSION**			
Summary of evidence	24	Summarize the main findings including the strength of evidence for each main outcome; consider their relevance to key groups (e.g., healthcare providers, users, and policy makers).	11
Limitations	25	Discuss limitations at study and outcome level (e.g., risk of bias), and at review-level (e.g., incomplete retrieval of identified research, reporting bias).	11
Conclusions	26	Provide a general interpretation of the results in the context of other evidence, and implications for future research.	12, 13
**FUNDING**			
Funding	27	Describe sources of funding for the systematic review and other support (e.g., supply of data); role of funders for the systematic review.	16

From: Moher D, Liberati A, Tetzlaff J, Altman DG, The PRISMA Group (2009). Preferred Reporting Items for Systematic Reviews and Meta-Analyses: The PRISMA Statement. PLoS Med 6(7): e1000097. doi:10.1371/journal.pmed1000097

For more information, visit: www.prisma-statement.org.

For peer review only - http://bmjopen.bmj.com/site/about/guidelines.xhtml
